# Efficient synthesis of dipeptide analogues of α-fluorinated β-aminophosphonates

**DOI:** 10.3762/bjoc.16.69

**Published:** 2020-04-16

**Authors:** Marcin Kaźmierczak, Henryk Koroniak

**Affiliations:** 1 Faculty of Chemistry, Adam Mickiewicz University in Poznań, Uniwersytetu Poznańskiego 8, 61-614 Poznań, Poland; 2 Centre for Advanced Technologies, Adam Mickiewicz University in Poznań, Uniwersytetu Poznańskiego 10, 61-614 Poznań, Poland

**Keywords:** dipeptide analogues, fluorinated aminophosphonates, fluorine, nucleophilic fluorination, phosphorus

## Abstract

Herein, we present an efficient synthesis of dipeptide analogues of α-fluorinated β-aminophosphonates. Each step of the synthesis was optimized to provide excellent yields. Moreover, the absolute configuration of the obtained compounds was determined by X-ray analysis, which proved the stereochemistry that was proposed based on NMR studies.

## Introduction

The chemistry of fluorinated aminophosphonates is constantly being developed, mainly due to their wide spectra of applications. What is more, they are valuable targets for biomedical investigations. Due to the strong interest in this type of compounds, several marvelous reviews about the synthesis and application of fluorinated aminophosphonates have been published in recent years [1–3]. To the best of our knowledge, among the many applications of fluorinated aminophosphonates and aminophosphonic acid derivatives, they exhibit antiviral [4–5], antibacterial [6] and antifungal [7] activities. Moreover, α-fluorinated phosphonates can be considered as hydrolytically stable mimics of naturally occurring phosphates [8]. Due to the fact that enzyme binding may depend on the C–F stereochemistry, the synthesis of such compounds with a specific configuration is very important [9–11]. α-Fluorinated phosphonates can be prepared by many different protocols [12–16].

**Figure 1:**
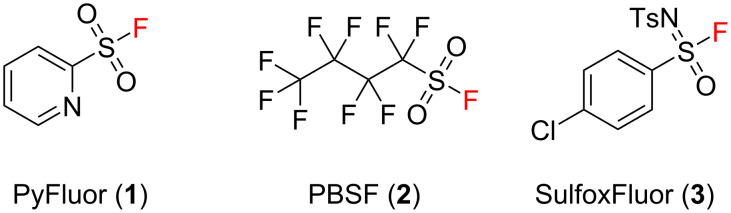
Chemical structure of PyFluor, PBSF and SulfoxFluor.

Nucleophilic fluorination is one of the fundamental reactions in organic chemistry in the field of the synthesis of building blocks with a potential biological activity. To date, scientists have developed many nucleophilic fluorinating reagents [17–19]. Among others, we can distinguish a family of such chemicals having in their structure a sulfonyl fluoride system. For example 2-pyridinesulfonyl fluoride (PyFluor, **1**) [20–21] or perfluorobutanesulfonyl fluoride (PBSF, **2**) [22–23] (Figure 1). On the other hand, Hu just recently presented a novel deoxyfluorination reagent with a similar structure to **1** and **2**, containing a sulfonimidoyl instead of sulfonyl group. 4-Chloro-*N*-tosylbenzene-1-sulfonimidoyl fluoride (SulfoxFluor, **3**, Figure 1) may be an interesting alternative to **1** or **2**. It is not commercially available yet, however, it can be obtained from inexpensive materials [24].

Very often small organic molecules containing a fluorine atom are transformed into biologically active compounds. As an example, we can consider peptide bond formation between an amine and a carboxylic acid. This transformation is a very important reaction in organic synthesis and therefore, many coupling reagents are available on the market [25–29].

Our previous studies have shown that the regioselectivity of the nucleophilic fluorination of amino alcohols can be controlled depending on the fluorinating reagent used [30–31]. This work presents the optimization of nucleophilic fluorination conditions. What is more, we discuss the effect of the base used on the regioselectivity of the fluorination reaction. The absolute configuration of the obtained compounds was determined and confirmed by X-ray analysis. Furthermore, we present the use of α-fluorinated β-aminophosphonates as building blocks in the synthesis of their dipeptide analogues. In addition, we show the results of the use of several coupling reagents in the synthesis of amide bonds.

## Results and Discussion

**Scheme 1:**
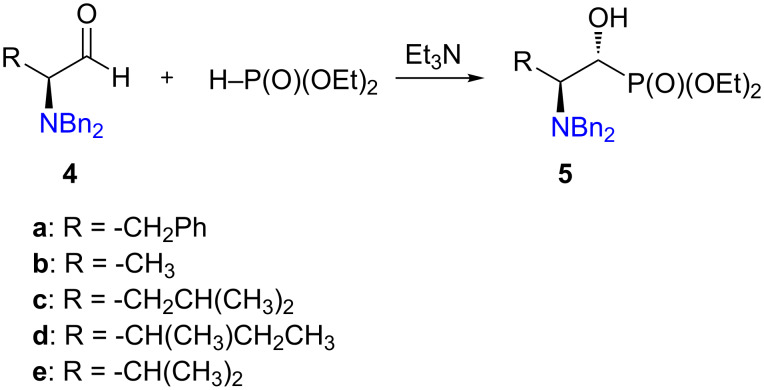
Synthesis of **5**.

Our goal was to increase the efficiency of the synthesis of α-fluorinated β-aminophosphonate dipeptide analogues **15**. In the first stage, we optimized Pudovik's reaction (Scheme 1) [32].

In our previous studies, the Pudovik reaction worked with satisfactory yield and diastereoselectivity [30]. Unfortunately, we were not able to isolate the pure *anti*-isomer in every case. The latest research shows that this reaction can successfully be carried out under milder conditions, without any solvent and in the presence of a weak base [33]. After testing many modifications, it turned out that the use of triethylamine (1 equiv) as a base, and without a solvent, gave the best results with aldehydes **4** (1 equiv) and diethyl phosphite (1 equiv). These conditions not only increased the diastereoselectivity of the Pudovik reaction, but also improved its yield drastically (Table 1). The implementation of this allowed us to obtain the *anti*-isomer (1*R*,2*S*) in very good yields.

**Table 1:** Optimization of reaction conditions.

entry	product	*anti*/*syn*^a^	*anti*/*syn*^b^	yield^c^

1	**5a**	91:9	99:1	86%
2	**5b**	86:14	99:1	71%
3	**5c**	90:10	99:1	75%
4	**5d**	92:8	99:1	88%
5	**5e**	90:10	99:1	79%

^a^Crude reaction mixture ratio based on ^31^P NMR. ^b^Ratio after isolation based on ^31^P NMR. ^c^Yield of the amino alcohol **5** after isolation.

**Figure 2:**
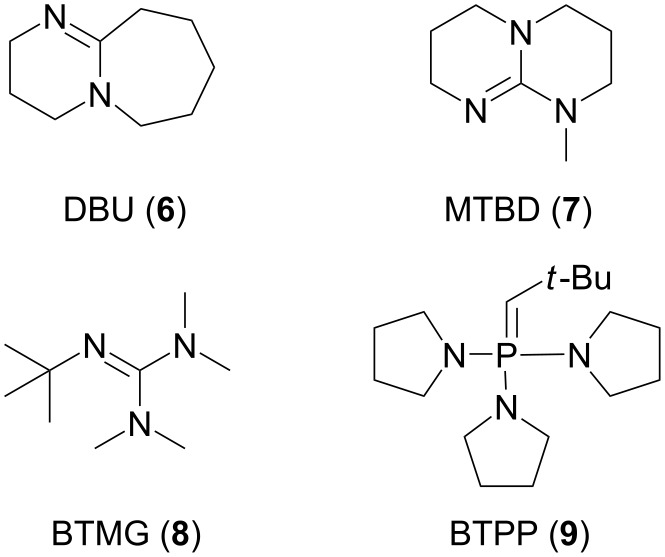
Chemical structure bases.

**Scheme 2:**
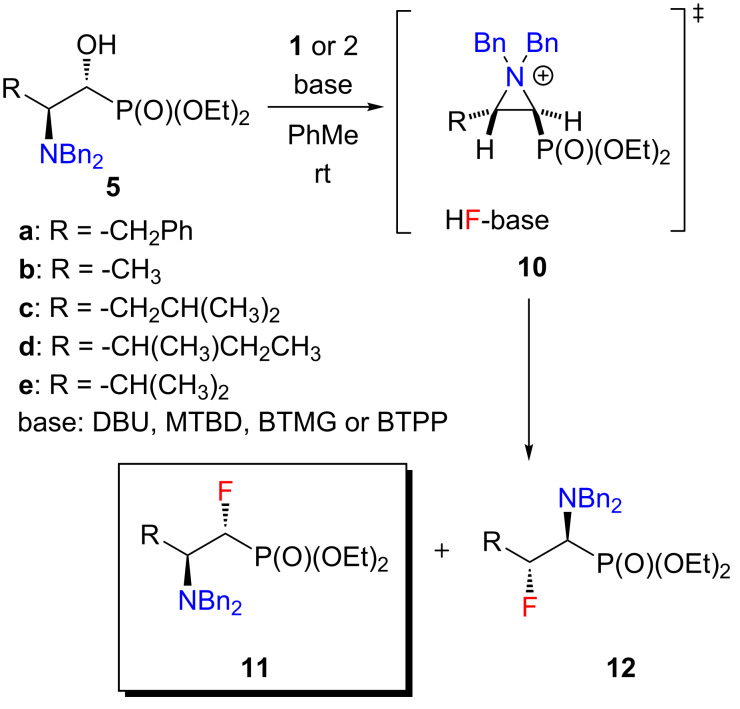
Synthesis of **11**.

There are not many literature examples of the regioselective fluorination of amino alcohols using by PyFluor (**1**) or PBSF (**2**). In general, the fluorination reactions with these reagents require the use of a base as an activator [20]. Doyle demonstrated the combination between PyFluor or PBSF and bases such as 1,8-diazabicyclo[5.4.0]undec-7-ene (DBU, **6**), 7-methyl-1,5,7-triazabicyclo[4.4.0]dec-5-ene (MTBD, **7**), 2-*tert*-butyl-1,1,3,3-tetramethylguanidine (BTMG) **8**, *tert*-butylimino-tri(pyrrolidino)phosphorane (BTPP, **9**, Figure 2) [21]. We screened the alcohols **5** against sulfonyl fluoride reagents **1** and **2** and a combination of selected bases **6–9** (Scheme 2). All reactions during the optimization step were carried out on a 0.1 mmol scale under an argon atmosphere while the reaction medium was toluene.

In the first stage of the optimization three representative amino alcohols were chosen. Each bearing a different group in the side chain: an aromatic (**5a**), a small (**5b**) and a large aliphatic substituent (**5c**). Unfortunately, under the tested conditions, we did not observe a complete regioselectivity of the fluorination reaction. Nevertheless, in each case, the use of PyFluor as a nucleophilic fluorinating reagent and MTBD as an activator improved the regioselectivity of this reaction, as well as, the yield of the α-isomer **11** (best results are shown in Table 2, for all tested conditions see Table S1 in Supporting Information File 1). In our experience, there was no significant correlation between steric bulkiness of the base (from DBU to BTPP) used, and the yield of α-isomer **11** formation. It is worth mentioning that application of PBSF gives slightly worse results in each case. We assume it is associated with a much greater reactivity of this reagent in relation to PyFluor.

**Table 2:** Optimization of reaction conditions.

entry	product	reagent	base	**11**/**12** ratio^a^	yield^b^

1	**11a**	PyFluor	MTBD	73:27	61%
2	**11b**	PyFluor	MTBD	60:40	47%
3	**11c**	PyFluor	MTBD	87:13	70%
4	**11d**	PyFluor	MTBD	68:32	59%
5	**11e**	PyFluor	MTBD	82:18	69%

^a^Crude reaction mixture ratio based on ^31^P NMR and ^19^F NMR; ^b^Yield of α-fluorides **11** after isolation.

The mechanism of the PyFluor-mediated deoxyfluorination of the α-hydroxy-β-aminophosphonates **5** was previously proposed. Based on spectroscopic studies (^19^F,^1^H-HOESY, ^1^H,^1^H-NOESY, as well as *J*-couplings) a relative configuration of *N*,*N*-dibenzyl-protected α-fluoro-β-aminophosphonates **11** was established as (1*R*,2*S*), but we failed to crystallize these compounds [31]. That is why the removal of the benzyl protecting group from fluorides **11** and the transfer of the free amines **13** into the salts **14** was applied. The salts **14** could be then crystallized and subjected to X-ray studies.

**Scheme 3:**
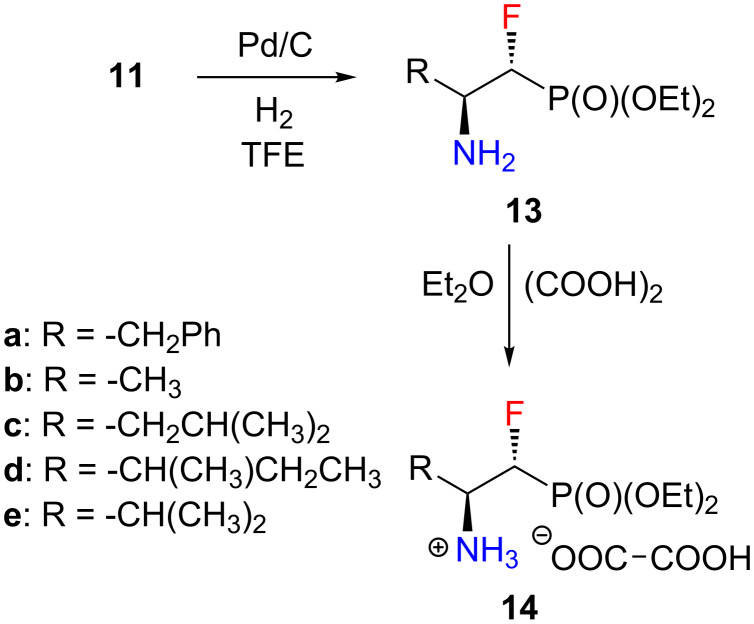
Synthesis of **13** and **14**.

A standard *N*−debenzylation protocol was employed to remove the benzyl protecting group. The hydrogenolysis reaction was catalyzed by palladium on carbon (Pd/C), and was carried out in trifluoroethanol (TFE) as a solvent [34–35]. The free amines **13** were converted into stable oxalate salts **14** with quantitative yields. The precipitation reactions proceeded in the presence of 1 equiv of oxalic acid in diethyl ether (Scheme 3) [36]. Unlike amines **13**, salts **14** are very stable and can be stored for months at room temperature.

**Figure 3:**
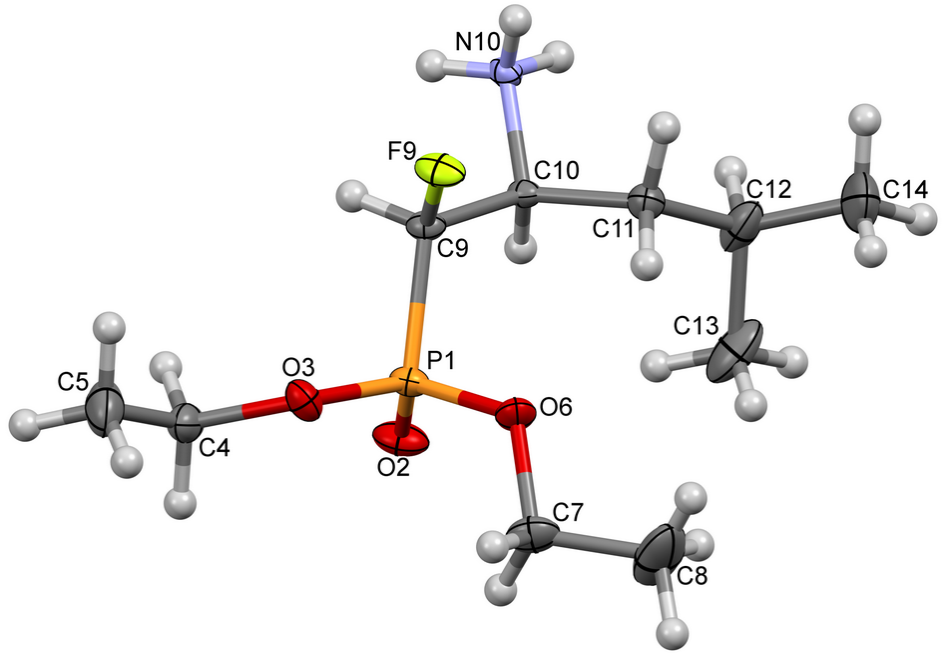
Molecular structure of compound (1*R*,2*S*)-**14c** (ORTEP image).

What is more, we attempted to crystallize at least one derivative to confirm the absolute stereochemistry of the obtained compounds. In order to obtain a single crystal suitable for X-ray diffraction studies, various crystallization techniques were tested [37–38]. Crystallization of derivative **14c** from D_2_O brought the expected results. The analysis confirmed the structure of the resulting compound **14c**. The absolute configuration of **14c** is consistent with the stereochemistry we proposed based on NMR studies. The crystals of **14c** contained the (1*R*,2*S*)-diastereoisomer (Figure 3). The correct absolute configuration was determined on the basis of Flack and Parson’s parameters, as well as of slightly better R factors for the correct model.

**Scheme 4:**
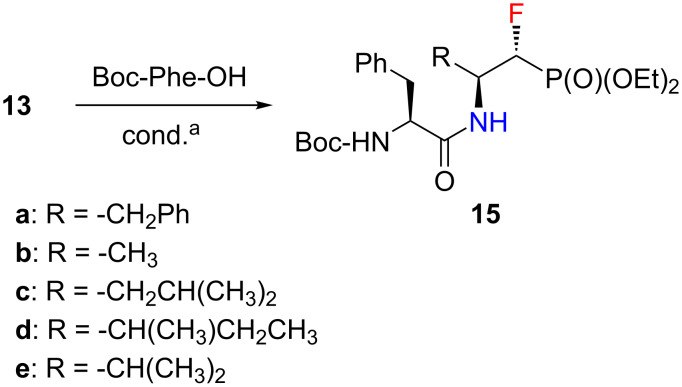
Synthesis of **15**. ^a^Conditions are given in the Experimental section.

Moreover, amines **13** were used as substrates in the formation of peptide bonds with *N-*Boc-phenylalanine (Boc-Phe-OH, Scheme 4). For this purpose we examined several coupling reagents available on the market. We chose amine **13a** as a model substrate. In each case, the amine–acid coupling reaction proceeded with good to excellent yields (Table 3). At the beginning a mixture of *N*,*N*′-dicyclohexylcarbodiimide (DCC) [39] and 1-hydroxybenzotriazole (HOBt) [40] was used. The reaction took place “only” in good yield (63%). Crude products required very careful purification due to the byproduct formed during the reaction – *N*,*N*′-dicyclohexylurea (DCU), which is insoluble in the solvents used in the synthesis (CH_2_Cl_2_, DMF). Replacing DCC with *N*-(3-dimethylaminopropyl)-*N*′-ethylcarbodiimide hydrochloride (EDCl) [41] significantly increased the reaction yield (86%). What is more, the reaction byproduct is water-soluble and easy to remove by extraction. Nevertheless, HOBt was still used as an additive. This reagent has recently been reported to exhibit explosive properties [42]. Fortunately, stable 1-hydroxybenzotriazole substitutes are available on the market, and can be used in the reaction of peptide bond formation [43]. One of them is OxymaPure [44] which was successfully employed as a replacement for HOBt. Using EDCI/OxymaPure conditions, an even greater yield of the product formation (88%) was observed, however, the reaction still required a long reation time. Finally, COMU [45] as a coupling reagent was applied. The model reaction took place with a yield of 92% after just two hours. COMU allowed to obtain the dipeptide analogue **15a** not only with an excellent yield, but also in a very short time. Under these conditions, the remaining amines **13** were reacted, to receive a series of dipeptide analogues of α-fluorinated β-aminophosphonates **15**.

**Table 3:** Optimization of reaction conditions.

entry	product	reagent	additive	base	time	yield^a^

1	15a	DCC	HOBt	Et_3_N	24 h	63%
2	15a	EDCI	HOBt	Et_3_N	18 h	86%
3	15a	EDCI	OxymaPure	DIPEA	18 h	88%
4	15a	COMU	–	DIPEA	2 h	92%
5	15b	COMU	–	DIPEA	3 h	91%
6	15c	COMU	–	DIPEA	3 h	94%
7	15d	COMU	–	DIPEA	3 h	92%
8	15e	COMU	–	DIPEA	3 h	92%

^a^Yield of dipeptide analogues **15** after isolation.

## Conclusion

In summary, an efficient approach to the synthesis of dipeptide analogues of α-fluorinated β-aminophosphonates **15** was presented. These compounds were prepared in a four-step sequence starting from *N*,*N*-dibenzylamino aldehydes **5**. Each step of the synthesis has been optimized to provide very good yields (Pudovik, deoxyfluorination and amine–acid coupling reactions). Furthermore, the absolute configuration of the obtained compounds has been confirmed by X-ray analysis, which is consistent with the stereochemistry proposed based on NMR studies. Dipeptide analogues of α-fluorinated β-aminophosphonates **15** presented in this publication, after deprotection of the amine function appear to be attractive compounds with potential biological activity.

## Experimental

### General methods

^1^H NMR, ^13^C NMR, ^19^F NMR and ^31^P NMR spectra were obtained on a Bruker ASCEND 400 (400 MHz) and a Bruker ASCEND 600 (600 MHz) spectrometer. All 2D spectra were recorded on a Bruker ASCEND 600 (600 MHz) spectrometer. ^1^H NMR chemical shifts were expressed in parts per million downfield from tetramethylsilane (TMS) as an internal standard (δ = 0) in CDCl_3_ or CD_3_CN. ^13^C NMR chemical shifts were expressed in parts per million downfield from CDCl_3_ (δ = 77.0) or CD_3_CN (δ = 1.39) as internal standards. ^19^F NMR chemical shifts were expressed in parts per million upfield from CFCl_3_ as an internal standard (δ = 0) in CDCl_3_ or CD_3_CN. ^31^P NMR Chemical shifts were expressed in parts per million in CDCl_3_ or CD_3_CN. High-resolution mass spectra were recorded by electron spray (ESIMS) techniques using a QToF Impact HD Bruker spectrometer. Reagent grade chemicals were used. Solvents were dried with CaH_2_ (CH_2_Cl_2_), NaH (Et_2_O), P_2_O_5_ (PhMe), and distilled under argon atmosphere. All moisture sensitive reactions were carried out under an argon atmosphere using ovendried glassware. TLC was performed on Merck Kieselgel 60-F254 with EtOAc/hexane and MeOH/CHCl_3_ as developing systems, and products were detected by inspection under UV light (254 nm) and with a solution of potassium permanganate. Merck Kieselgel 60 (0.063–0.200 μm), Merck Kieselgel 60 (0.040–0.063 μm), and Merck Kieselgel 60 (0.015–0.004 μm), were used for column chromatography.

X-ray diffraction data for **14c** were collected at 100(1) K by the ω-scan technique on Rigaku four-circle Xcalibur diffractometer (Eos detector) with graphite-monochromatized Mo Kα radiation (λ = 0.71073 Å). The data were corrected for Lorentz-polarization and absorption effects [46]. Accurate unit cell parameters were determined by a least-squares fit of 4300 reflections of highest intensity, chosen from the whole experiment. The structure was solved with SHELXT and refined with the full-matrix least-squares procedure on F2 by SHELXL [47]. All non-hydrogen atoms were refined anisotropically, hydrogen atoms were placed in the calculated positions and refined as ‘riding model’ with the isotropic displacement parameters set at 1.2 (1.5 for methyl and hydroxy groups) times the U_eq_ value for appropriate non-hydrogen atom. As the crystals were obtained from D_2_O solution, this solvent was used for final model (although, no difference between refinements with H_2_O and D_2_O could be found). The correct absolute configuration was determined on the basis of Flack and Parson’s parameters, as well as of slightly better R factors for the correct model. The crystallographic data for the structural analysis has been deposited with the Cambridge Crystallographic Data Centre, CCDC No. 1977323 (**14c**). Copies of this information may be obtained free of charge from http://www.ccdc.cam.ac.uk.

### Procedure for the synthesis of amino alcohols **5**

TEA (1,0 equiv) was added under an argon atmosphere to a mixture of a stirred solution of diethyl phosphite (1,0 equiv) and the appropriate aldehyde **4** (1,0 equiv). The reaction mixture was stirred at room temperature overnight, diluted with 20 mL of water and extracted with ethyl acetate (3 × 15 mL). The organic layers were washed with NaCl_sat._, dried over MgSO_4_ or Na_2_SO_4_, filtrated and concentrated under reduced pressure. The crude products were isolated using column chromatography (chloroform → chloroform/methanol 100:0.5 v/v). The NMR spectroscopic data for amino alcohols **5** were in good agreement with our previous research [30–31,48].

### Procedure for the synthesis of fluorides **11**

PyFluor (1,2 equiv) or PBSF (1,2 equiv) and base (2 equiv) were added under an argon atmosphere to a stirred solution of amino alcohol **5** (1mmol) in 2,5 mL PhMe, under an argon atmosphere. The reaction mixture was stirred at room temperature until completion of the reaction monitored by TLC (3–18 h). The reaction mixture was then diluted with 20 mL of water and extracted with ethyl acetate (3 × 15 mL). The organic layers were dried over MgSO_4_ or Na_2_SO_4_, filtrated and concentrated under reduced pressure. The crude products were isolated using column chromatography (*n*-hexane/ethyl acetate 90:10, v/v → *n*-hexane/ethyl acetate 60:40, v/v). The NMR spectroscopic data for fluorides **11** were in good agreement with our previous research [31].

### Procedure for the synthesis of amines **13**

The amines **13** were prepared in a similar manner as described in [35]. Fluoride **11** (1 equiv) was dissolved in 5 mL of TFE and then 10% Pd/C (20% v/v) was added. The solution was stirred under an atmosphere of hydrogen at room temperature for 3 days. After this time the catalyst was filtered off (Celite), the solvent was evaporated and the crude product was purified using flash chromatography (chloroform/methanol 100:0, v/v chloroform/methanol → 50:0.5 v/v).

### Procedure for the synthesis of oxalates **14**

The oxalates **14** were prepared in a similar manner as described in [35]. A solution of amine **13** (1 equiv) was dissolved in anhydrous diethyl ether and was added dropwise to a vigorously stirred solution of oxalic acid (1 equiv) in diethyl ether under an argon atmosphere. The mixture was left overnight in a freezer. The next day, the precipitate was filtered off to give a white solid.

### Procedure for the synthesis of dipeptide analogues **15**

**DCC/HOBt conditions:** The analogue **15a** was prepared in a similar manner as described in [35]. To a cooled (0 °C) solution of amine **13** (1.05 equiv) in methylene chloride a solution of a Boc-Phe-OH (1 equiv), HOBt (1.25 equiv) in a 3:7 CH_2_Cl_2_/DMF solution and DCC (1.25 equiv) under an argon atmosphere were added. Stirring was continued at this temperature for 30 min and then the solution was stirred for 24 h at room temperature. The DCU was filtered off, and the filtrate was washed with cold solutions of 1 M NaOH, water, 1 M citric acid, water, 1 M NaHCO_3_, and water. The organic extract was dried over anhydrous MgSO_4_. The drying agent was filtered off and the solvent was evaporated. The crude product was purified by flash chromatography (*n*-hexane/ethyl acetate 60:40 → 50:50).

**EDCI/HOBt conditions:** To a cooled (0 °C) solution of amine **13** (1.05 equiv) in DMF, Boc-Phe-OH (1 equiv), TEA (1 equiv), HOBt (1.3 equiv) and EDCI (1.25 equiv) were added. Stirring was continued at this temperature for 30 min and then the solution was stirred for 18 h at room temperature. The reaction mixture was then diluted with 20 mL of ethyl acetate and washed with NaHCO_3sat_, 1 M HCl, and NaCl_sat_. The organic layer was dried over MgSO_4_ or Na_2_SO_4_, filtrated and concentrated under reduced pressure. The crude product was purified by flash chromatography (*n*-hexane/ethyl acetate 60:40 → 50:50).

**EDCI/OxymaPure conditions:** Boc-Phe-OH (1 equiv), OxymaPure (1.1 equiv), and EDCI (1.1 equiv) were mixed in DMF at 0 °C under an argon atmosphere. The reaction mixture was stirred for 5 min at 0 °C to preactivate the acid and generate the active ester, and then DIPEA (1 equiv) followed by amine **13** (1 equiv) were added. Stirring was continued at this temperature for 30 min and then the solution was stirred for 18 h at room temperature. The reaction mixture was then diluted with 20 mL of ethyl acetate and washed with NaHCO_3sat_, 1 M HCl, and NaCl_sat_. The organic layer was dried over MgSO_4_ or Na_2_SO_4_, filtrated and concentrated under reduced pressure. The crude product was purified by flash chromatography (*n*-hexane/ethyl acetate 60:40 → 50:50).

**COMU conditions:** COMU (1 equiv) was added at 0 °C to a mixture of Boc-Phe-OH (1 equiv) and DIPEA (2 equiv) in DMF under an argon atmosphere. The reaction mixture was activated for 5 min, followed by the addition of amine **13** (1 equiv) in DMF. Stirring was continued at this temperature for 30 min and then the solution was stirred for 2–3 h at room temperature (monitored by ^31^P or ^19^F NMR). The reaction mixture was then diluted with 20 mL of ethyl acetate and washed with NaHCO_3sat_, 1 M HCl, and NaCl_sat_. The organic layer was dried over MgSO_4_ or Na_2_SO_4_, filtrated and concentrated under reduced pressure. The crude product was purified by flash chromatography (*n*-hexane/ethyl acetate 60:40 → 50:50).

## Supporting Information

File 1 Optimization of reaction conditions data, characterization data, copies of NMR spectra for compounds **13–15**, single crystal X-ray data for compound **14c**.

File 2 CIF file of **14c**.
