# Supplementary material for: Efficient synthesis of dipeptide analogues of α-fluorinated β-aminophosphonates
Source: Beilstein J Org Chem. 2020 Apr 16;16:756–62. doi: 10.3762/bjoc.16.69 (PMC7176923; doi:10.3762/bjoc.16.69)
Supplement: File 1 — Optimization of reaction conditions data, characterization data, copies of NMR spectra for compounds 13–15, single crystal X-ray data for compound 14c. [file Beilstein_J_Org_Chem-16-756-s001.pdf]

BEILSTEIN JOURNAL OF ORGANIC CHEMISTRY

Supporting Information

for

Efficient synthesis of dipeptide analogues of α-fluorinated β-aminophosphonates

Marcin Kaźmierczak and Henryk Koroniak

Beilstein J. Org. Chem. **2020,** *16,* 756–762. doi:10.3762/bjoc.16.69

Optimization of reaction conditions data, characterization data, copies of NMR spectra for compounds 13–15, single crystal X-ray data for compound 14c

License and Terms: This is a supporting information file under the terms of the Creative Commons Attribution License (http://creativecommons.org/licenses/by/4.0). Please note that the reuse, redistribution and reproduction in particular requires that the authors and source are credited.
The license is subject to the *Beilstein Journal of Organic Chemistry* terms and conditions: (https://www.beilstein-journals.org/bjoc)

Table of Contents

1. Optimization of reaction conditions. S3
2. Characterization data S5
    a) Characterization of amines 13 .... S5
    b) Characterization of oxalates 14 .... S7
    c) Characterization of dipeptide analogues 15 .... S10
3. H, ^{13}C, ^{19}F, ^{31}P and 2D NMR spectra of compounds 13–15 .... S14
4. Crystallographic data S54

1. Optimization of reaction conditions

Table S1: Optimization of reaction conditions.

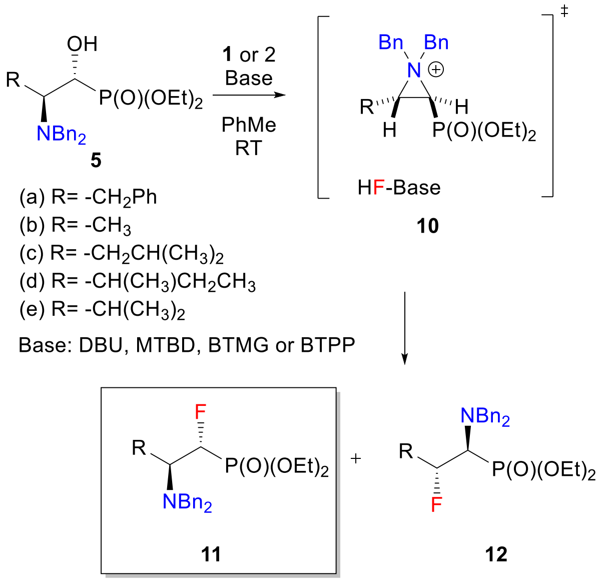

Entry	Product	Reagent	Base	**11/12** ratio[a]	Yield[b]
1	11a	PyFluor	DBU	67:33	58%
**2**	**11a**	**PyFluor**	**MTBD**	**73:27**	**61%**
3	11a	PyFluor	BTMG	66:34	-
4	11a	PyFluor	BTPP	65:35	-
5	11a	PBSF	DBU	59:41	-
6	11a	PBSF	MTBD	63:37	-
7	11a	PBSF	BTMG	66:34	-
8	11a	PBSF	BTPP	63:27	-
9	11b	PyFluor	DBU	55:45	42%
**10**	**11b**	**PyFluor**	**MTBD**	**60:40**	**47%**
11	11b	PyFluor	BTMG	53:47	-
12	11b	PyFluor	BTPP	56:44	-
13	11b	PBSF	DBU	34:66	-
14	11b	PBSF	MTBD	49:51	-

15	11b	PBSF	BTMG	48:52	-
16	11b	PBSF	BTPP	58:42	-
17	11c	PyFluor	DBU	82:18	68%
**18**	**11c**	**PyFluor**	**MTBD**	**87:13**	**70%**
19	11c	PyFluor	BTMG	74:26	-
20	11c	PyFluor	BTPP	80:20	-
21	11c	PBSF	DBU	68:32	-
22	11c	PBSF	MTBD	77:23	-
23	11c	PBSF	BTMG	74:26	-
24	11c	PBSF	BTPP	77:23	-
25	11d	PyFluor	DBU	62:38	51%
**26**	**11d**	**PyFluor**	**MTBD**	**68:32**	**59%**
27	11e	PyFluor	DBU	76:24	61%
**28**	**11e**	**PyFluor**	**MTBD**	**82:18**	**69%**

[a]Crude reaction mixture ratio based on ^{31}P NMR and ^{19}F NMR; [b]Yield of α-fluorides **11** after isolation.

2. Characterization data

a. Characterization of amines 13

Diethyl ((1*R*,2*S*)-2-amino-1-fluoro-3-phenylpropyl)phosphonate (13a): Colorless oil (61 %): **^{1}H NMR** (600 MHz, $CDCl_3$) δ = 7.24 (t, *J* = 7.5 Hz, 2H, Ar*H*), 7.17 (d, *J* = 6.5 Hz, 3H, Ar*H*), 4.43 (ddd, *J* = 45.5, 7.5, 2.7 Hz, 1H, C*H*FP), 4.18 (q, *J* = 7.3 Hz, 2H, OCH_2CH_3), 4.13 (q, *J* = 7.3 Hz, 2H, OCH_2CH_3), 3.50 – 3.39 (m, 1H, PhCHHC*H*N), 3.08 (dd, *J* = 13.7, 2.3 Hz, 1H, PhC*H*HCHN), 2.61 (dd, *J* = 13.7, 8.7 Hz, 1H, PhCH*H*CHN), 1.44 (br.s, 2H, NH_2), 1.29 (td, *J* = 7.1, 5.6 Hz, 6H, 2 x OCH_2CH_3). **^{13}C{/^{1}H} NMR** (151 MHz, $CDCl_3$) δ = 137.24, 129.45, 128.44, 126.52 (4 x s, Ar), 90.75 (dd, *J* = 186.2, 165.2 Hz, *C*HFP), 63.26 (d, *J* = 6.8 Hz, OCH_2CH_3), 62.61 (d, *J* = 6.6 Hz, OCH_2CH_3), 52.41 (dd, *J* = 20.4, 1.8 Hz, $PhCH_2CH$), 38.69 (dd, *J* = 8.8, 5.0 Hz, $PhCH_2$), 16.33 (d, *J* = 5.9 Hz, OCH_2CH_3), 16.27 (d, *J* = 5.6 Hz, OCH_2CH_3). **^{19}F NMR** (565 MHz, $CDCl_3$) = δ -208.56 (ddd, *J* = 75.1, 45.4, 11.1 Hz). **^{19}F{/^{1}H} NMR** (565 MHz, $CDCl_3$) δ = -208.56 (d, *J* = 75.1 Hz). **^{31}P{/^{1}H} NMR** (243 MHz, $CDCl_3$) δ = 17.35 (d, *J* = 74.9 Hz). **HRMS (ESI)** calcd for $C_{13}H_{22}FNO_3P$ (SM+H]$^+$): 290.1321, found: 290.1318.

Diethyl ((1*R*,2*S*)-2-amino-1-fluoropropyl)phosphonate (13b): Colorless oil (47 %): **^{1}H NMR** (600 MHz, $CDCl_3$) δ = 4.37 (ddd, *J* = 45.7, 6.6, 2.9 Hz, 1H, C*H*FP), 4.22 – 4.09 (m, 4H, 2 x OCH_2CH_3), 3.33 (tp, *J* = 13.1, 6.6 Hz, 1H, CH_3CH), 1.83 (br.s, 2H, NH_2), 1.30 (t, *J* = 7.1 Hz, 6H, 2 x OCH_2CH_3), 1.20 (dd, *J* = 6.6, 2.1 Hz, 3H, CH_3). **^{13}C{/^{1}H} NMR** (151 MHz, $CDCl_3$) δ = 92.42 (dd, *J* = 185.7, 163.9 Hz, *C*HFP), 63.18 (d, *J* = 7.0 Hz, OCH_2CH_3), 62.54 (d, *J* = 6.7 Hz, OCH_2CH_3), 47.23 (dd, *J* = 20.6, 2.1 Hz, CH_3CH), 18.80 (dd, *J* = 7.8, 5.7 Hz, CH_3), 16.26 (d, *J* = 5.9 Hz, OCH_2CH_3), 16.22 (d, *J* = 5.7 Hz, OCH_2CH_3). **^{19}F NMR** (565 MHz, $CDCl_3$) δ = -210.42 (ddd, *J* = 76.2, 45.7, 13.3 Hz). **^{19}F{/^{1}H} NMR** (565 MHz, $CDCl_3$) δ = -210.42 (d, *J* = 75.9 Hz). **^{31}P{/^{1}H}**

NMR (243 MHz, $CDCl_3$) δ = 16.93 (d, *J* = 76.3 Hz). **HRMS (ESI)** calcd for $C_7H_{18}FNO_3P$ ($[M+H]^+$): 214.1008, found: 214.1009.

Diethyl ((1*R*,2*S*)-2-amino-1-fluoro-4-methylpentyl)phosphonate (13c): Colorless oil (70 %): **^{1}H NMR** (600 MHz, $CDCl_3$) = δ 4.38 (ddd, *J* = 45.6, 6.6, 3.0 Hz, 1H, C*HF*P), 4.24 – 4.14 (m, 4H, 2 x OC*H*$_2$CH$_3$), 3.22 (ddddd, *J* = 16.5, 13.4, 10.1, 6.5, 3.7 Hz, 1H (CH$_3$)$_2$CHCHHC*H*N), 1.82 – 1.71 (m, 1H, (CH$_3$)$_2$C*H*), 1.51 (br. s, 2H, N*H*$_2$), 1.46 (dddd, *J* = 13.6, 9.7, 3.8, 1.5 Hz, 1H, (CH$_3$)$_2$CHC*H*H), 1.29 (td, *J* = 7.1, 1.4 Hz, 6H, 2 x OCH$_2$C*H*$_3$), 1.27 – 1.23 (m, 1H, (CH$_3$)$_2$CHCH*H*), 0.89 (d, *J* = 6.7 Hz, 3H, C*H*$_3$), 0.84 (d, *J* = 6.6 Hz, 3H, C*H*$_3$). **^{13}C{/^{1}H} NMR** (151 MHz, $CDCl_3$) δ = 92.13 (dd, *J* = 184.8, 164.1 Hz, *C*HFP), 63.08 (d, *J* = 6.9 Hz, O*C*H$_2$CH$_3$), 62.31 (d, *J* = 6.7 Hz, O*C*H$_2$CH$_3$), 49.76 (d, *J* = 20.1 Hz, (CH$_3$)$_2$CHCH$_2$*C*H), 41.64 (dd, *J* = 6.7, 4.3 Hz, (CH$_3$)$_2$CH*C*H$_2$), 24.15 (s, (CH$_3$)$_2$*C*H), 23.46 (s, *C*H$_3$), 21.20 (s, *C*H$_3$), 16.25 (d, *J* = 5.7 Hz, OCH$_2$*C*H$_3$), 16.19 (d, *J* = 5.8 Hz, OCH$_2$*C*H$_3$). **^{19}F NMR** (565 MHz, $CDCl_3$) δ = -208.55 (ddd, *J* = 77.7, 45.8, 13.4 Hz). **^{19}F{/^{1}H} NMR** (565 MHz, $CDCl_3$) δ = -209.06 (d, *J* = 77.6 Hz). **^{31}P{/^{1}H} NMR** (243 MHz, $CDCl_3$) δ = 17.20 (d, *J* = 77.9 Hz). **HRMS (ESI)** calcd for $C_{10}H_{24}FNO_3P$ ($[M+H]^+$): 256.1478, found: 256.1472.

Diethyl ((1*R*,2*S*)-2-amino-1-fluoro-3-methylpentyl)phosphonate (13d): Colorless oil (59 %): **^{1}H NMR** (600 MHz, $CDCl_3$) δ = 4.53 (ddt, *J* = 45.8, 9.0, 2.3 Hz, 1H, C*H*FP), 4.15 (tdd, *J* = 14.5, 7.2, 5.4 Hz, 4H, 2 x OC*H*$_2$CH$_3$), 3.16 – 3.05 (m, 1H, CH$_3$CH$_2$CH(CH$_3$)C*H*N), 1.73 – 1.63 (m, 1H, CH$_3$CH$_2$C*H*(CH$_3$)), 1.56 (br. s, 2H, N*H*$_2$), 1.52 – 1.43 (m, 1H, CH$_3$C*H*H), 1.30 (td, *J* = 7.1, 1.8 Hz, 6H, 2 x OCH$_2$C*H*$_3$), 1.15 – 1.00 (m, 1H, CH$_3$CH*H*), 0.93 (dd, *J* = 6.9, 1.8 Hz, 3H, CH(C*H*$_3$)), 0.85 (td, *J* = 7.4, 1.8 Hz, 3H, CH$_2$C*H*$_3$). **^{13}C{/^{1}H} NMR** (151 MHz, $CDCl_3$) δ = 89.48 (dd, *J* = 186.3, 165.3 Hz, *C*HFP), 63.16 (d, *J* = 7.0 Hz, O*C*H$_2$CH$_3$), 62.48 (d, *J* = 6.7 Hz, O*C*H$_2$CH$_3$), 55.43 (d, *J* = 19.6 Hz, CH$_3$CH$_2$CH(CH$_3$)*C*H), 35.65 (dd, *J* = 9.3, 3.2 Hz, CH$_3$CH$_2$*C*H(CH$_3$)), 22.70 (s, CH$_3$*C*HH), 16.28 (d, *J* = 5.6 Hz, OCH$_2$*C*H$_3$), 16.22 (d, *J* = 6.0 Hz,

OCH_2CH_3), 15.63 (s, CH(CH_3)), 11.58 (s, CH_2CH_3). **^{19}F NMR** (565 MHz, $CDCl_3$) δ = -206.79 (ddd, *J* = 75.4, 45.9, 7.8 Hz). **^{19}F{/^{1}H} NMR** (565 MHz, $CDCl_3$) δ = -206.78 (d, *J* = 75.4 Hz). **^{31}P{/^{1}H} NMR** (243 MHz, $CDCl_3$) δ = 18.49 (d, *J* = 76.1 Hz). **HRMS (ESI)** calcd for $C_{10}H_{24}FNO_3P$ ($[M+H]^+$): 256.1478, found: 256.1479.

Diethyl ((1*R*,2*S*)-2-amino-1-fluoro-3-methylbutyl)phosphonate (13e): Colorless oil (69 %): **^{1}H NMR** (400 MHz, $CDCl_3$) δ = 4.45 (ddd, *J* = 45.8, 9.0, 2.7 Hz, 1H, C*H*FP), 4.25 – 4.04 (m, 4H, 2 x OC$H_2$$CH_3$), 3.13 – 2.99 (m, 1H, $(CH_3)_2$CHC*H*N), 2.08 – 1.88 (m, 1H, $(CH_3)_2$C*H*), 1.53 (br. s, 2H, NH_2), 1.30 (t, *J* = 7.1 Hz, 6H, 2 x OCH_2CH_3), 0.95 (d, *J* = 7.0 Hz, 3H, CH_3), 0.84 (d, *J* = 6.8 Hz, 3H, CH_3). **^{13}C{/^{1}H} NMR** (101 MHz, $CDCl_3$) δ = 89.58 (dd, *J* = 186.6, 165.6 Hz, *C*HFP), 63.14 (d, *J* = 7.1 Hz, O*C*H_2CH_3), 62.50 (d, *J* = 6.7 Hz, O*C*H_2CH_3), 55.46 (d, *J* = 19.6 Hz, $(CH_3)_2$CH*C*H), 28.14 (dd, *J* = 9.7, 3.8 Hz, $(CH_3)_2$*C*H), 19.44 (s, *C*H_3), 16.27 (d, *J* = 5.4 Hz, OCH_2*C*H_3), 16.21 (d, *J* = 5.7 Hz, OCH_2*C*H_3), 15.09 (s, *C*H_3). **^{19}F NMR** (376 MHz, $CDCl_3$) δ = -207.62 (ddd, *J* = 75.2, 45.8, 7.8 Hz). **^{19}F{/^{1}H} NMR** (376 MHz, $CDCl_3$) δ = -207.62 (d, *J* = 75.3 Hz). **^{31}P{/^{1}H} NMR** 162 MHz, $CDCl_3$) δ = 18.44 (d, *J* = 75.5 Hz). **HRMS (ESI)** calcd for $C_9H_{22}FNO_3P$ ($[M+H]^+$): 242.1321, found: 242.1319.

b. Characterization of oxalates 14

(1*R*,2*S*)-1-(Diethoxyphosphoryl)-1-fluoro-3-phenylpropan-2-aminium carboxyformate (14a): White solid (99 %): **^{1}H NMR** (600 MHz, D_2O) δ 7.45 (ddd, *J* = 8.2, 4.9, 1.9 Hz, 2H, Ar*H*), 7.42 – 7.38 (m, 1H, Ar*H*), 7.36 (d, *J* = 7.5 Hz, 2H, Ar*H*), 5.45 – 5.32 (m, 1H, C*H*FP), 4.36 (dq, *J* = 14.9, 7.1 Hz, 4H, 2 x OC$H_2$$CH_3$), 4.27 – 4.12 (m, 1H, PhCHHC*H*N) 3.40 (dd, *J* = 14.8, 4.6 Hz, 1H, PhCH*H*CHN), 3.05 (dd, *J* = 14.8, 10.7 Hz, 1H, PhC*H*HCHN), 1.41 (td, *J* = 7.1, 5.5 Hz, 6H, 2 x OCH_2CH_3). **^{13}C{/^{1}H} NMR** (151 MHz, D_2O) δ = 166.08 (s, *C*=O), 134.29, 129.30, 129.27, 127.99 (4 x s, Ar), 87.00 (dd, *J* = 184.1, 171.3 Hz, *C*HFP), 65.84 (d, *J* = 7.2 Hz, O*C*H_2CH_3), 65.61 (d, *J* = 7.1 Hz, O*C*H_2CH_3), 53.34 (dd, *J* = 19.5, 5.8 Hz, PhCH_2*C*H), 33.04 (d, *J*

= 5.0 Hz, Ph*C*H_2), 15.66 (d, *J* = 3.4 Hz, OCH_2*C*H_3), 15.63 (d, *J* = 3.0 Hz, OCH_2*C*H_3). **^{19}F NMR** (565 MHz, D_2O) δ = -219.44 (ddd, *J* = 73.2, 44.6, 25.4 Hz). **^{19}F{/^{1}H} NMR** (565 MHz, D_2O) δ = -219.42 (d, *J* = 76.2 Hz). **^{31}P{/^{1}H} NMR** (243 MHz, D_2O) δ = 14.20 (d, *J* = 76.3 Hz). **HRMS (ESI)** calcd for $C_{13}H_{22}FNO_3P$ ($[M]^+$): 290.1321, found: 290.1313.

(1*R*,2*S*)-1-(Diethoxyphosphoryl)-1-fluoropropan-2-aminium carboxyformate (14b): White solid (99 %): **^{1}H NMR** (600 MHz, D_2O) δ = 5.23 (ddd, *J* = 45.4, 8.8, 2.4 Hz, 1H, C*H*FP), 4.32 – 4.17 (m, 4H, 2 x OC*H*$_2$$CH_3$), 3.91 (dt, *J* = 27.2, 8.0 Hz, 1H, CH_3C*H*), 1.40 (d, *J* = 6.9 Hz, 3H, C*H*$_3$), 1.30 (t, *J* = 7.0 Hz, 6H, 2 x OCH_2C*H*$_3$). ^{13}C**{/^{1}H} NMR** (151 MHz, D_2O) δ = 166.43 (s, *C*=O), 87.45 (dd, *J* = 184.2, 172.0 Hz, *C*HFP), 65.74 (d, *J* = 7.0 Hz, O*C*$H_2$$CH_3$), 65.48 (d, *J* = 7.2 Hz, O*C*$H_2$$CH_3$), 47.73 (dd, *J* = 20.1, 7.3 Hz, CH_3*C*H), 15.63 (s, OCH_2*C*H_3), 15.59 (s, OCH_2*C*H_3), 12.36 (d, *J* = 5.3 Hz, *C*H_3). **^{19}F NMR** (565 MHz, D_2O) δ = -222.63 (ddd, *J* = 74.1, 45.5, 27.4 Hz). **^{19}F{/^{1}H} NMR** (565 MHz, D_2O) δ = -222.61 (d, *J* = 75.6 Hz). **^{31}P{/^{1}H} NM**R (243 MHz, D_2O) δ = 14.36 (d, *J* = 75.2 Hz). **HRMS (ESI) calcd** for $C_7H_{18}FNO_3P$ ($[M]^+$): 214.1008, found: 214.1002.

(1*R*,2*S*)-1-(Diethoxyphosphoryl)-1-fluoro-4-methylpentan-2-aminium carboxyformate (14c): White solid (99 %): **^{1}H NMR** (600 MHz, D_2O) δ = 5.35 (ddd, *J* = 44.3, 8.5, 2.8 Hz, 1H, C*H*FP), 4.45 – 4.25 (m, 4H, 2 x OC*H*$_2$$CH_3$), 4.04 – 3.87 (m, 1H, $(CH_3)_2$CHCHHC*H*N), 1.82 – 1.60 (m, 3H, $(CH_3)_2$C*H*, $(CH_3)_2$CHC*H*$_2$), 1.38 (t, *J* = 7.1 Hz, 6H, 2 x OCH_2C*H*$_3$), 0.98 (d, *J* = 4.9 Hz, 3H, C*H*$_3$), 0.94 (d, *J* = 5.2 Hz, 3H, C*H*$_3$). ^{13}C**{/^{1}H} NMR** (151 MHz, D_2O) δ = 167.32 (s, *C*=O), 90.22 (dd, *J* = 183.5, 171.1 Hz, *C*HFP), 68.43 (d, *J* = 7.3 Hz, O*C*$H_2$$CH_3$), 68.11 (d, *J* = 7.0 Hz, O*C*$H_2$$CH_3$), 53.03 (dd, *J* = 18.9, 5.0 Hz, $(CH_3)_2$CHCH_2*C*H), 38.65 (d, *J* = 4.3 Hz, $(CH_3)_2$CH*C*H_2), 26.35 (s, $(CH_3)_2$*C*H), 24.78 (s, *C*H_3), 23.07 (s, *C*H_3), 18.36 (d, *J* = 1.5 Hz, OCH_2*C*H_3), 18.32 (d, *J* = 1.5 Hz, OCH_2*C*H_3). **^{19}F NMR** (565 MHz, D_2O) δ = -218.32 (ddd, *J* =

77.9, 44.5, 24.2 Hz). **^{19}F{/^{1}H} NMR** (565 MHz, D_2O) δ = -218.31 (d, *J* = 77.2 Hz). **^{31}P{/^{1}H} NMR** (243 MHz, D_2O) δ = 14.57 (d, *J* = 77.7 Hz). **HRMS (ESI)** calcd for $C_{10}H_{24}FNO_3P$ ($[M]^+$): 256.1478, found: 256.1471. **Crystal data:** $(C_{10}H_{24}FNO_3P)^+\cdot(C_2HO_4)^-\cdot D_2O$, M_r = 365.33, orthorhombic, $P2_12_12_1$, a = 6.34660(18) Å, b = 12.0072(3) Å, c = 24.5409(7) Å, V = 1870.14(9) Å^3, Z = 4, d_x = 1.30 g·cm^{-3}, F(000) = 776, μ = 0.192 cm^{-1}, 10638 reflection collected, 3600 symmetry independent (R_{int} = 2.43%), 3313 with I>2σ(I). Final R[I>2σ(I)] = 0.0406, wR2[I>2σ(I)] = 0.0727, R[all reflections] = 0.0484, wR2[all reflections] = 0.0753, S = 1.056, ($\Delta\rho_{max}$/ $\Delta\rho_{min}$) = 0.25/-0.29 e·Å^{-3}.

(1*R*,2*S*)-1-(Diethoxyphosphoryl)-1-fluoro-3-methylpentan-2-aminium carboxyformate (14d): White solid (99 %): **^{1}H NMR** (600 MHz, D_2O) δ = 5.45 (ddd, *J* = 42.4, 7.3, 4.4 Hz, 1H, C*H*FP), 4.45 – 4.19 (m, 4H, 2 x OC*H2*CH3), 3.78 (dddd, *J* = 23.1, 18.9, 8.2, 4.4 Hz, 1H, $CH_3CH_2CH(CH_3)C$*H*N), 2.08 – 1.97 (m, 1H, CH_3CH_2C*H*(CH_3)), 1.72 – 1.61 (m, 1H, CH_3C*H*H), 1.38 (t, *J* = 7.0 Hz, 6H, 2 x OCH_2C*H*3), 1.29 – 1.16 (m, 1H, CH_3CH*H*), 1.07 (d, *J* = 6.8 Hz, 3H, CH(C*H*3)), 0.94 (t, *J* = 7.4 Hz, 3H, CH_2C*H*3). **^{13}C{/^{1}H} NMR** (101 MHz, D_2O) δ = 167.73 (s, *C*=O), 89.28 (dd, *J* = 181.9, 169.2 Hz, *C*HFP), 68.43 (d, *J* = 7.3 Hz, O*C*H_2CH_3), 68.18 (d, *J* = 7.1 Hz, O*C*H_2CH_3), 59.17 (d, *J* = 17.7 Hz, $CH_3CH_2CH(CH_3)$*C*H), 36.29 (d, *J* = 3.9 Hz, CH_3CH_2*C*H(CH_3)), 26.77 (d, *J* = 2.1 Hz, CH_3*C*HH), 18.34 (s, OCH_2C*H*3), 18.31 (s, OCH_2C*H*3), 17.61 (s, CH(*C*H_3)), 12.40 (s, CH_2*C*H_3). **^{19}F NMR** (565 MHz, D_2O) δ = -212.83 (ddd, *J* = 78.4, 42.4, 18.8 Hz). **^{19}F{/^{1}H} NMR** (565 MHz, D_2O) δ = -212.83 (d, *J* = 78.4 Hz). **^{31}P{/^{1}H} NMR** (243 MHz, D_2O) δ = 14.99 (d, *J* = 78.2 Hz). **HRMS (ESI)** calcd for $C_{10}H_{24}FNO_3P$ ($[M]^+$): 256.1478, found: 256.1482.

(1*R*,2*S*)-1-(Diethoxyphosphoryl)-1-fluoro-3-methylbutan-2-aminium carboxyformate phosphonate (14e): White solid (99 %): **^{1}H NMR** (600 MHz, D_2O) δ = 5.44 (ddd, *J* = 42.5, 7.5, 4.2 Hz, 1H, C*H*FP), 4.42 – 4.27 (m, 4H, 2 x OC*H*2CH3),

3.70 (dddd, *J* = 23.0, 19.2, 8.1, 4.2 Hz, 1H, $(CH_3)_2CHCHN$), 2.26 (h, *J* = 6.8 Hz, 1H, $(CH_3)_2CH$), 1.38 (td, *J* = 7.1, 1.3 Hz, 6H, 2 x OCH_2CH_3), 1.10 (d, *J* = 2.8 Hz, 3H, CH_3), 1.09 (d, *J* = 3.6 Hz, 3H, CH_3). **^{13}C{/^{1}H} NMR** (151 MHz, D_2O) δ = 168.24 (s, *C*=O), 89.23 (dd, *J* = 181.7, 169.5 Hz, *C*HFP), 68.42 (d, *J* = 7.2 Hz, OCH_2CH_3), 68.18 (d, *J* = 7.1 Hz, OCH_2CH_3), 60.36 (d, *J* = 17.6 Hz, $(CH_3)_2CHCH$), 30.12 (d, *J* = 4.5 Hz, $(CH_3)_2CH$), 21.81 (s, CH_3), 20.52 (d, *J* = 2.1 Hz, CH_3), 18.34 (s, OCH_2CH_3), 18.31 (s, OCH_2CH_3). **^{19}F NMR** (565 MHz, D_2O) δ = -213.48 (ddd, *J* = 77.7, 42.4, 19.2 Hz). **^{19}F{/^{1}H} NMR** (565 MHz, D_2O) δ = -213.47 (d, *J* = 78.2 Hz). **^{31}P{/^{1}H} NMR** (243 MHz, D_2O) δ = 14.89 (d, *J* = 77.8 Hz). **HRMS (ESI)** calcd for $C_9H_{22}FNO_3P$ ($[M]^+$): 242.1321, found: 242.1330.

c. Characterization of dipeptide analogues 15

***tert*-Butyl ((*S*)-1-(((1*R*,2*S*)-1-(diethoxyphosphoryl)-1-fluoro-3-phenylpropan-2-yl)amino)-1-oxo-3-phenylpropan-2-yl)carbamate (15a):** White solid (92 %): **^{1}H NMR** (600 MHz, $CDCl_3$) δ= 7.29 – 6.97 (m, 10H, Ar*H*), 6.75 (br.d, *J* = 8.4 Hz, 1H, N*H*), 4.87 (br.d, *J* = 7.9 Hz, 1H, N*H*), 4.81 – 4.42 (m, 2H, C*H*FP, PhCHHC*H*N), 4.28 – 4.20 (m, 1H, PhCHHC*H*CO), 4.14 (dp, *J* = 22.2, 7.6 Hz, 4H, 2 x OCH_2CH_3), 3.05 (dd, *J* = 14.4, 6.2 Hz, 1H, PhC*H*HCHN), 2.94 (dd, *J* = 14.1, 6.3 Hz, 1H, PhC*H*HCHCO), 2.89 – 2.74 (m, 2H, PhCH*H*CHN, PhCH*H*CHCO), 1.34 – 1.25 (m, 15H, $C(CH_3)_3$, 2 x OCH_2CH_3). **^{13}C{/^{1}H} NMR** (151 MHz, $CDCl_3$) δ= 170.96 (s, *C*=O), 155.08 (s, *C*=O), 136.75, 136.45, 129.20, 129.01, 128.43, 126.68 (6 x s, Ar), 88.05 (dd, *J* = 184.6, 165.7 Hz, *C*HFP), 79.79 (s, $C(CH_3)_3$), 63.84 (d, *J* = 6.5 Hz, OCH_2CH_3), 62.84 (d, *J* = 6.9 Hz, OCH_2CH_3), 55.58 (s, $PhCH_2CHCO$), 51.39 (d, *J* = 20.3 Hz, $PhCH_2CHNH$), 38.20 (s, $PhCH_2CHCO$), 35.42 (d, *J* = 5.7 Hz, $PhCH_2CHNH$), 28.11 (s, $C(CH_3)_3$), 16.34 (s, OCH_2CH_3), 16.30 (s, OCH_2CH_3). **^{19}F NMR** (565 MHz, $CDCl_3$) δ= -215.54 (ddd, *J* = 80.1, 45.1, 22.4 Hz). **^{19}F{/^{1}H} NMR** (565 MHz, $CDCl_3$) δ= -215.54 (d, *J* =

79.6 Hz). ^{31}**P{/^{1}H} NMR** (243 MHz, $CDCl_3$) δ= 15.75 (d, *J* = 80.2 Hz). **HRMS (ESI)** calcd for $C_{27}H_{38}FN_2O_6PNa$ ([M+Na]$^+$): 559.2349, found: 559.2343.

***tert*-Butyl ((*S*)-1-(((1*R*,2*S*)-1-(diethoxyphosphoryl)-1-fluoropropan-2-yl)amino)-1-oxo-3-phenylpropan-2-yl)carbamate (15b):** White solid (91 %): ^{1}**H NMR** (600 MHz, $CDCl_3$) δ = 7.30 – 7.23 (m, 2H, Ar*H*), 7.24 – 7.17 (m, 3H, Ar*H*), 6.73 (br.d, *J* = 8.3 Hz, 1H, N*H*), 5.18 (br. s, 1H, N*H*), 4.63 (br. d, *J* = 45.8 Hz, 1H, C*H*FP), 4.51 – 4.40 (m, 1H, CH_3C*H*N), 4.40 – 4.33 (m, 1H, PhCHHC*H*CO), 4.25 – 4.13 (m, 4H, 2 x OC$H_2$$CH_3$), 3.12 – 2.96 (m, 2H, PhC*HH*), 1.38 (s, 9H, C(CH_3)$_3$), 1.37 – 1.29 (m, 6H, 2 x OCH_2CH_3), 1.25 (d, *J* = 7.0 Hz, 3H, CH_3). ^{13}**C{/^{1}H} NMR** (151 MHz, $CDCl_3$) δ = 170.69 (s, C=O), 155.24 (s, C=O), 136.46, 129.21, 128.45, 126.77 (4 x s, Ar), 89.56 (dd, *J* = 184.9, 166.0 Hz, *C*HFP), 79.90 (s, *C*(CH_3)$_3$), 63.53 (d, *J* = 6.7 Hz, O*C*$H_2$$CH_3$), 62.81 (d, *J* = 6.8 Hz, O*C*$H_2$$CH_3$), 55.56 (s, PhCHH*C*H), 45.66 (d, *J* = 19.9 Hz, CH_3C*H*NH), 38.48 (s, Ph*C*H_2CHCO), 28.12 (s, C(*C*H_3)$_3$), 16.30 (d, *J* = 3.8 Hz, OCH_2*C*H_3), 16.26 (d, *J* = 4.3 Hz, OCH_2*C*H_3), 14.73 (d, *J* = 6.1 Hz, *C*H_3). ^{19}**F NMR** (565 MHz, $CDCl_3$) δ = -220.48 (ddd, *J* = 76.1, 46.1, 26.5 Hz). ^{19}**F{/^{1}H} NMR** (565 MHz, $CDCl_3$) δ =-220.47 (d, *J* = 79.0 Hz). ^{31}**P{/^{1}H} NMR** (243 MHz, $CDCl_3$) δ = 15.18 (d, *J* = 79.0 Hz). **HRMS (ESI)** calcd for $C_{21}H_{35}FN_2O_6P$ ([M+H]$^+$): 461.2217, found: 461.2199.

***tert*-Butyl ((*S*)-1-(((1*R*,2*S*)-1-(diethoxyphosphoryl)-1-fluoro-4-methylpentan-2-yl)amino)-1-oxo-3-phenylpropan-2-yl)carbamate (15c):** White solid (94 %): ^{1}**H NMR** (600 MHz, CD_3CN) δ = 7.43 – 7.14 (m, 5H), 7.03 (br. d, *J* = 9.0 Hz, 1H, N*H*), 5.69 (br. d, *J* = 8.5 Hz, 1H, N*H*), 4.68 (ddd, *J* = 45.5, 5.5, 3.2 Hz, 1H, C*H*FP), 4.56 – 4.36 (m, 1H, (CH_3)$_2$CHCHHC*H*N), 4.28 (dt, *J* = 14.3, 6.8 Hz, 1H, PhCHHC*H*CO), 4.17 (“p”, *J* = 7.3 Hz, 4H, 2 x OC$H_2$$CH_3$), 3.11 (dd, *J* = 13.9, 5.7 Hz, 1H, PhC*H*H), 2.88 (dd, *J* = 13.9, 8.8 Hz, 1H, PhCH*H*), 1.64 (dtt, *J* = 13.2, 10.0, 5.4 Hz, 1H, (CH_3)$_2$C*H*), 1.55 (dq, *J* = 13.9, 10.2 Hz, 2H, (CH_3)$_2$CHC*HH*), 1.37 (s, 9H, C(CH_3)$_3$),

1.33 (q, J = 6.8 Hz, 6H, 2 x OCH_2CH_3), 0.93 (d, J = 6.7 Hz, 3H, CH_3), 0.89 (d, J = 6.5 Hz, 3H, CH_3). **^{13}C{/^{1}H} NMR** (151 MHz, CD_3CN) δ = 172.31 (s, *C*=O), 156.34 (s, *C*=O), 138.65, 130.27, 129.23, 127.47 (4 x s, Ar), 91.33 (dd, J = 184.3, 164.0 Hz, *C*HFP), 79.89 (s, $C(CH_3)_3$), 64.18 (d, J = 6.8 Hz, OCH_2CH_3), 63.75 (d, J = 6.7 Hz, OCH_2CH_3), 56.87 (s, PhCHH*C*H), 48.62 (dd, J = 19.9, 5.7 Hz, $(CH_3)_2CHCH_2CH$), 38.48 (s, $PhCH_2CHCO$), 38.40 (d, J = 4.4 Hz, $(CH_3)_2CHCH_2$), 28.50 (s, $C(CH_3)_3$), 25.11 (s, $(CH_3)_2CH$), 23.84 (s, CH_3), 21.47 (s, CH_3), 16.80 (d, J = 0.9 Hz, OCH_2CH_3), 16.76 (d, J = 1.0 Hz, OCH_2CH_3). **^{19}F NMR** (565 MHz, CD_3CN) δ = -217.97 (ddd, J = 77.0, 45.5, 25.6 Hz). **^{19}F{/^{1}H}** NMR (565 MHz, CD_3CN) δ = -217.97 (d, J = 76.7 Hz). **^{31}P{/^{1}H} NMR** (243 MHz, CD_3CN) δ = 15.27 (d, J = 76.4 Hz). HRMS (ESI) calcd for $C_{24}H_{40}FN_2O_6PNa$ ($[M+Na]^+$): 525.2506, found: 525.2516.

***tert*-Butyl ((*S*)-1-(((1*R*,2*S*,3*S*)-1-(diethoxyphosphoryl)-1-fluoro-3-methylpentan-2-yl)amino)-1-oxo-3-phenylpropan-2-yl)carbamate (15d):** White solid (92 %): **^{1}H NMR** (600 MHz, CD_3CN) δ = 7.33 – 7.18 (m, 5H, Ar*H*), 7.11 (br. d, J = 10.1 Hz, 1H, N*H*), 5.70 (br. d, J = 8.7 Hz, 1H, N*H*), 4.93 (dt, J = 44.5, 5.3 Hz, 1H, C*H*FP), 4.46 – 4.33 (m, 1H, $CH_3CH_2CH(CH_3)CHN$), 4.34 – 4.24 (m, 1H, PhCHHC*H*CO), 4.20 – 4.12 (m, 4H, 2 x OCH_2CH_3), 3.22 (dd, J = 14.1, 4.8 Hz, 1H, PhC*H*H), 2.84 (dd, J = 14.1, 9.6 Hz, 1H, PhCH*H*), 1.84 – 1.75 (m, 1H, $CH_3CH_2CH(CH_3)$), 1.68 – 1.55 (m, 1H, CH_3CHH), 1.40 – 1.27 (m, 15H, $C(CH_3)_3$, 2 x OCH_2CH_3), 1.18 – 1.05 (m, 1H, CH_3CHH) 0.96 (d, J = 6.8 Hz, 3H, $CH(CH_3)$), 0.89 (t, J = 7.5 Hz, 3H, CH_2CH_3). **^{13}C{/^{1}H} NMR** (151 MHz, CD_3CN) δ = 172.54 (s, *C*=O), 156.48 (s, *C*=O), 139.00, 130.25, 129.21, 127.37 (4 x s, Ar), 88.89 (dd, J = 183.2, 165.6 Hz, *C*HFP), 79.87 (s, $C(CH_3)_3$), 64.37 (d, J = 6.7 Hz, OCH_2CH_3), 63.60 (d, J = 6.7 Hz, OCH_2CH_3), 57.02 (s, PhCHH*C*H), 54.45 (d, J = 19.9 Hz, $CH_3CH_2CH(CH_3)CH$), 38.13 (s, $PhCH_2CHCO$), 36.54 (s, $CH_3CH_2CH(CH_3)$), 28.51 (s, $C(CH_3)_3$), 24.97 (s, CH_3CHH), 16.81 (d, J = 5.5 Hz, OCH_2CH_3), 16.73 (d, J = 5.6 Hz, OCH_2CH_3), 16.32 (s, $CH(CH_3)$), 11.43 (s,

CH_2CH_3). **^{19}F NMR** (565 MHz, CD_3CN) δ = -210.36 (ddd, *J* = 77.0, 44.6, 16.0 Hz). **^{19}F{/^{1}H} NMR** (565 MHz, CD_3CN) δ = -210.36 (d, *J* = 77.0 Hz). **^{31}P{/^{1}H} NMR** (243 MHz, CD_3CN) δ = 16.40 (d, *J* = 76.5 Hz). **HRMS (ESI)** calcd for $C_{24}H_{40}FN_2O_6PNa$ ($[M+Na]^+$): 525.2506, found: 525.2498.

***tert*-Butyl ((*S*)-1-(((1*R*,2*S*)-1-(diethoxyphosphoryl)-1-fluoro-3-methylbutan-2-yl)amino)-1-oxo-3-phenylpropan-2-yl)carbamate (15e):** White solid (92 %): **^{1}H NMR** (600 MHz, CD_3CN) δ = 7.38 – 7.10 (m, 5H, Ar*H*), 6.98 (br. d, *J* = 10.1 Hz, 1H, N*H*), 5.61 (br. d, *J* = 8.7 Hz, 1H, N*H*), 4.82 (dt, *J* = 44.7, 5.4 Hz, 1H, C*H*FP), 4.37 – 4.25 (m, 1H, $(CH_3)_2CHCHN$), 4.28 – 4.21 (m, 1H, PhCHHC*H*CO), 4.17 – 4.08 (m, 4H, 2 x OCH_2CH_3), 3.18 (dd, *J* = 14.1, 4.9 Hz, 1H, PhC*H*H), 2.86 – 2.69 (m, 1H, PhCH*H*), 2.03 (h, *J* = 6.7 Hz, 1H, $(CH_3)_2CH$), 1.31 (s, 9H, $C(CH_3)_3$), 1.32 – 1.25 (m, 6H, 2 x OCH_2CH_3) 0.92 (d, *J* = 6.9 Hz, 3H, C*H*$_3$), 0.90 (d, *J* = 6.9 Hz, 3H, C*H*$_3$). **^{13}C{/^{1}H} NMR** (151 MHz, CD_3CN) δ = 172.60 (s, C=O), 156.52 (s, *C*=O), 139.01, 130.24, 129.24, 127.40 (4 x s, Ar), 88.97 (dd, *J* = 183.2, 165.6 Hz, *C*HFP), 79.90 (s, $C(CH_3)_3$), 64.34 (d, *J* = 6.7 Hz, OCH_2CH_3), 63.64 (d, *J* = 6.8 Hz, OCH_2CH_3), 57.07 (s, PhCHH*C*H), 54.77 (d, *J* = 19.8 Hz, $(CH_3)_2CHCHN$), 38.14 (s, Ph*C*H$_2$CHCO), 30.01 (s, $(CH_3)_2CH$), 28.49 (s, $C(CH_3)_3$), 20.33 (s, *C*H$_3$), 18.02 (s, *C*H$_3$), 16.79 (d, *J* = 5.5 Hz, OCH_2CH_3), 16.73 (d, *J* = 5.7 Hz, OCH_2CH_3). **^{19}F NMR** (565 MHz, CD_3CN) δ = -211.45 (ddd, *J* = 75.9, 44.7, 16.5 Hz). **^{19}F{/^{1}H} NMR** (565 MHz, CD_3CN) δ = -211.45 (d, *J* = 75.9 Hz). **^{31}P{/^{1}H} NMR** (243 MHz, CD_3CN) δ = 16.21 (d, *J* = 75.9 Hz). **HRMS (ESI)** calcd for $C_{23}H_{38}FN_2O_6PNa$ ($[M+Na]^+$): 511.2349, found: 511.2331.

3. ^{1}H, ^{13}C, ^{19}F, ^{31}P and 2D NMR Spectra of compounds 13-15

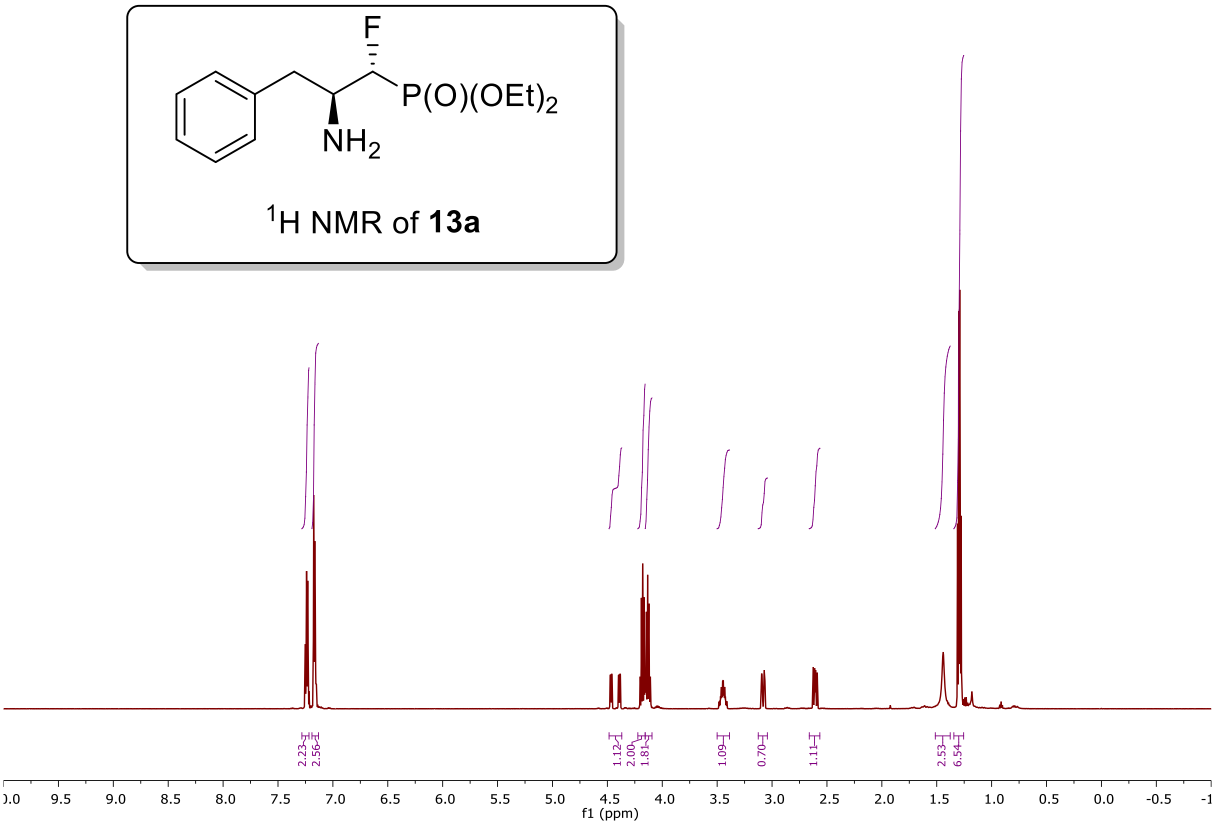

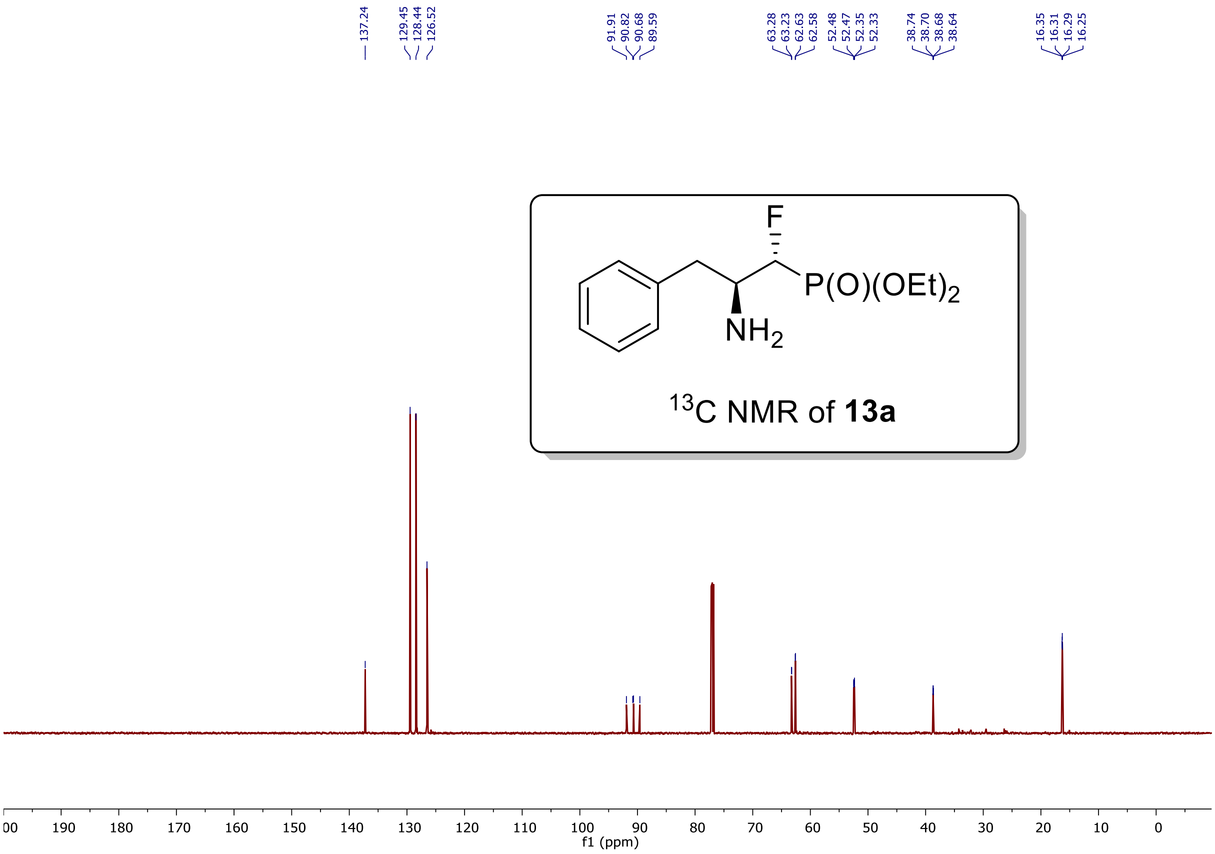

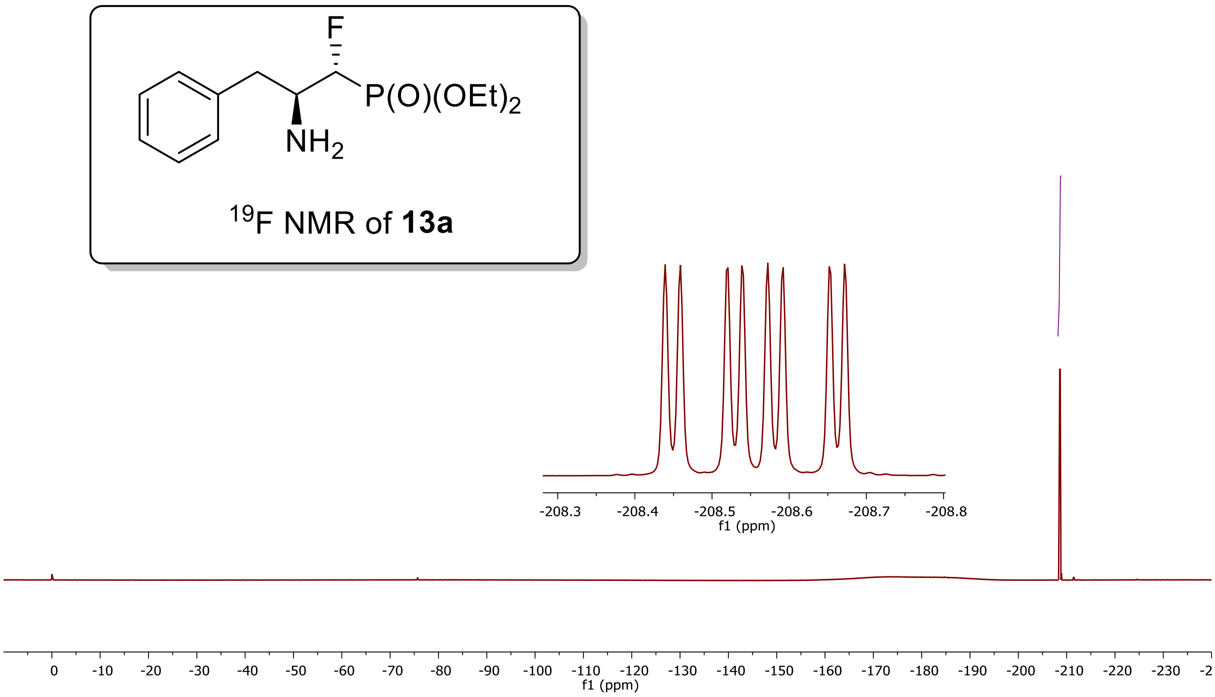
F
P(O)(OEt)2
NH2
19F NMR of 13a
-208.3
-208.4
-208.5
-208.6
-208.7
-208.8
f1 (ppm)
0
-10
-20
-30
-40
-50
-60
-70
-80
-90
-100
-110
-120
-130
-140
-150
-160
-170
-180
-190
-200
-210
-220
-230
-2
f1 (ppm)

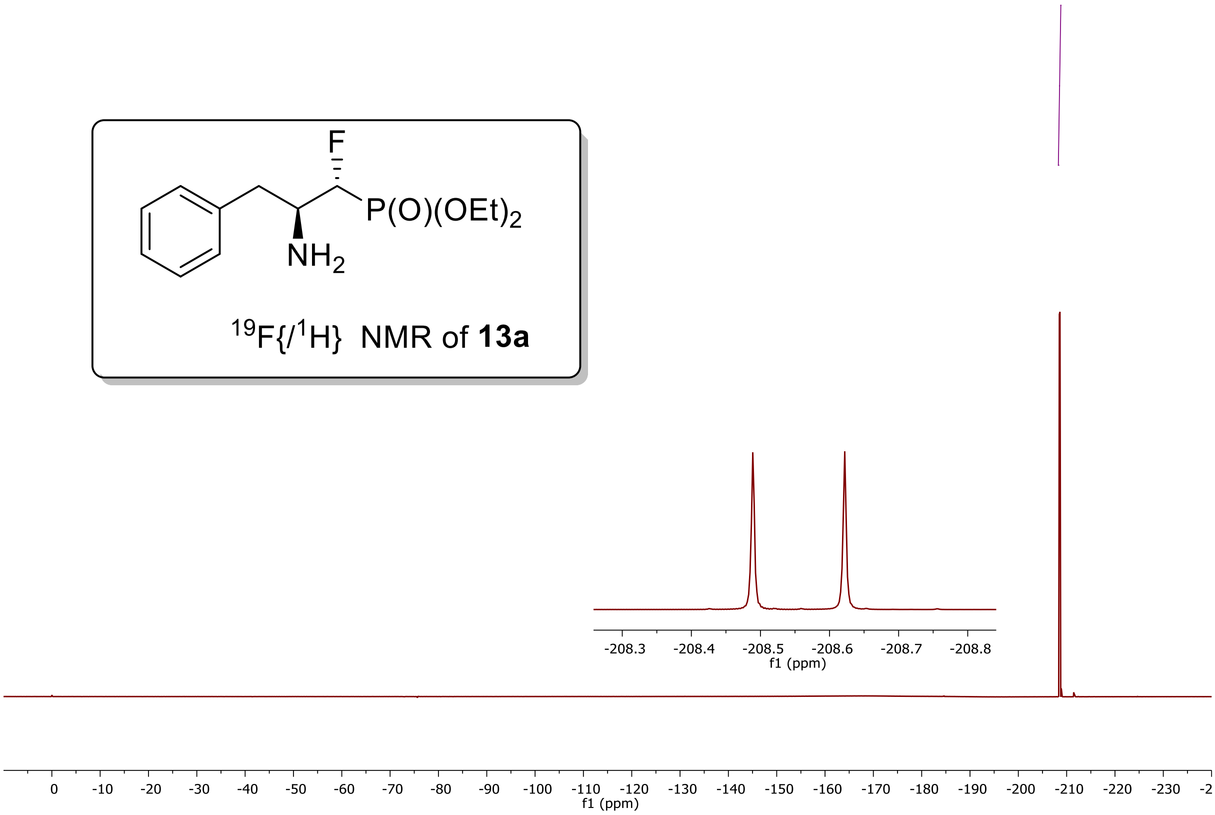
F
P(O)(OEt)2
NH2
19F{/1H} NMR of 13a
-208.3
-208.4
-208.5
-208.6
-208.7
-208.8
f1 (ppm)
0
-10
-20
-30
-40
-50
-60
-70
-80
-90
-100
-110
-120
-130
-140
-150
-160
-170
-180
-190
-200
-210
-220
-230
-2
f1 (ppm)

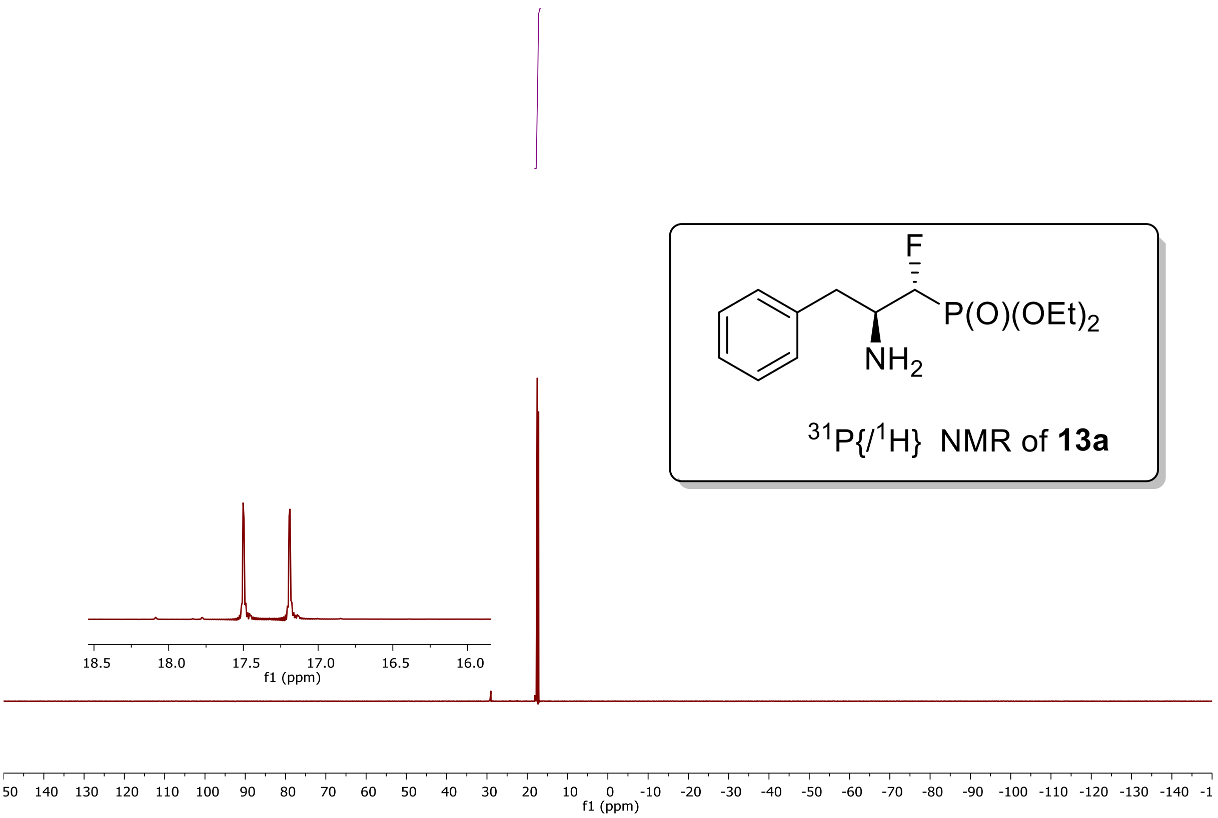
F
P(O)(OEt)2
NH2
31P{1H} NMR of 13a
18.5 18.0 17.5 17.0 16.5 16.0
f1 (ppm)
50 140 130 120 110 100 90 80 70 60 50 40 30 20 10 0 -10 -20 -30 -40 -50 -60 -70 -80 -90 -100 -110 -120 -130 -140
f1 (ppm)

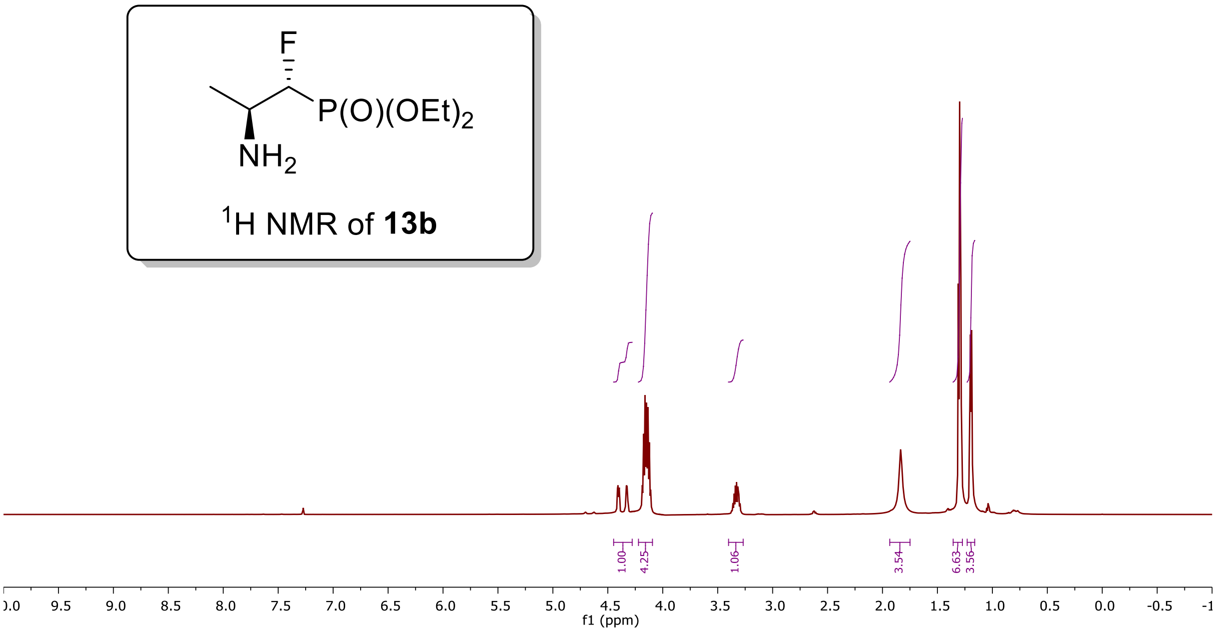
F
P(O)(OEt)2
NH2
1H NMR of 13b
1.00
4.25
1.06
3.54
6.63
3.56
9.5 9.0 8.5 8.0 7.5 7.0 6.5 6.0 5.5 5.0 4.5 4.0 3.5 3.0 2.5 2.0 1.5 1.0 0.5 0.0 -0.5
f1 (ppm)

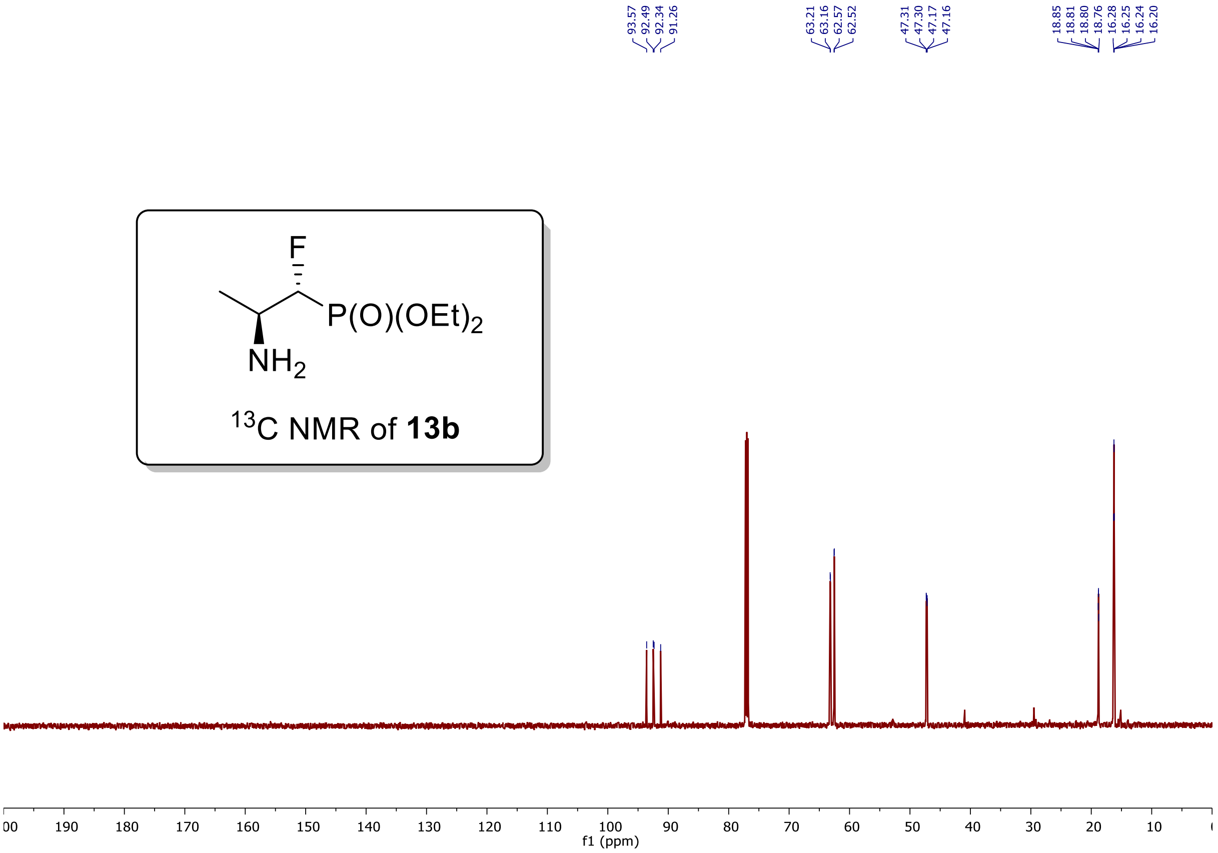
93.57
92.49
92.34
91.26
63.21
63.16
62.57
62.52
47.31
47.30
47.17
47.16
18.85
18.81
18.80
18.76
16.28
16.25
16.24
16.20
F
P(O)(OEt)2
NH2
13C NMR of 13b
f1 (ppm)

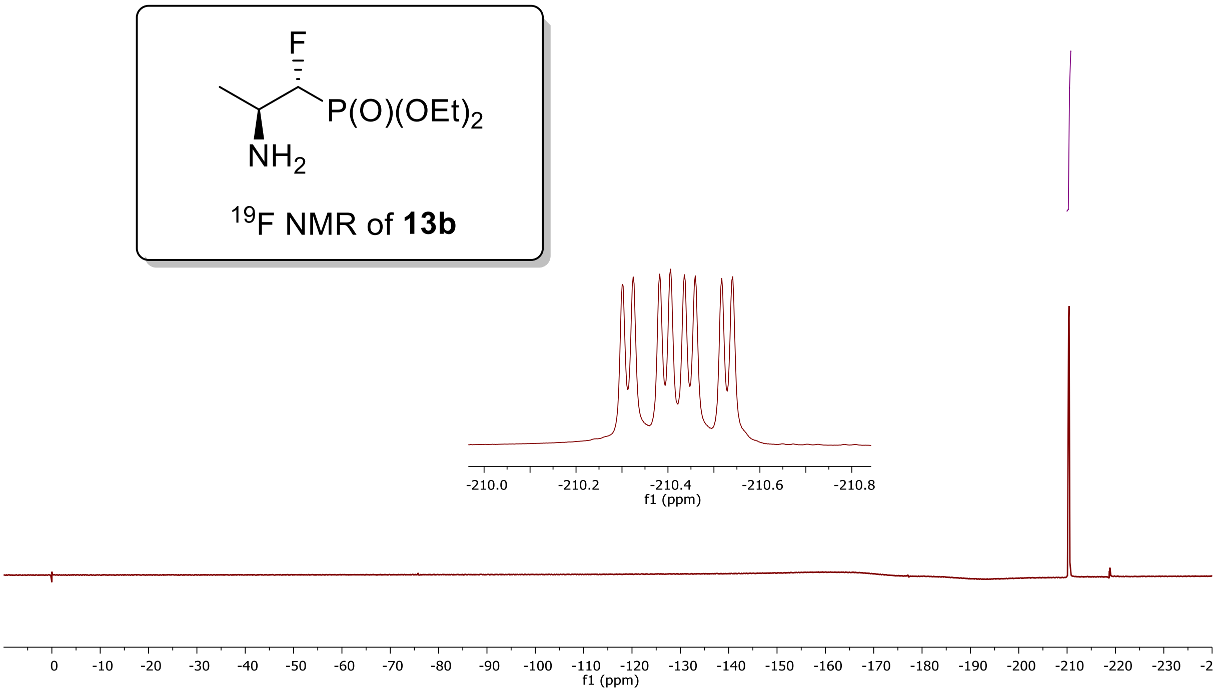
F
P(O)(OEt)2
NH2
19F NMR of 13b
-210.0
-210.2
-210.4
-210.6
-210.8
f1 (ppm)
f1 (ppm)

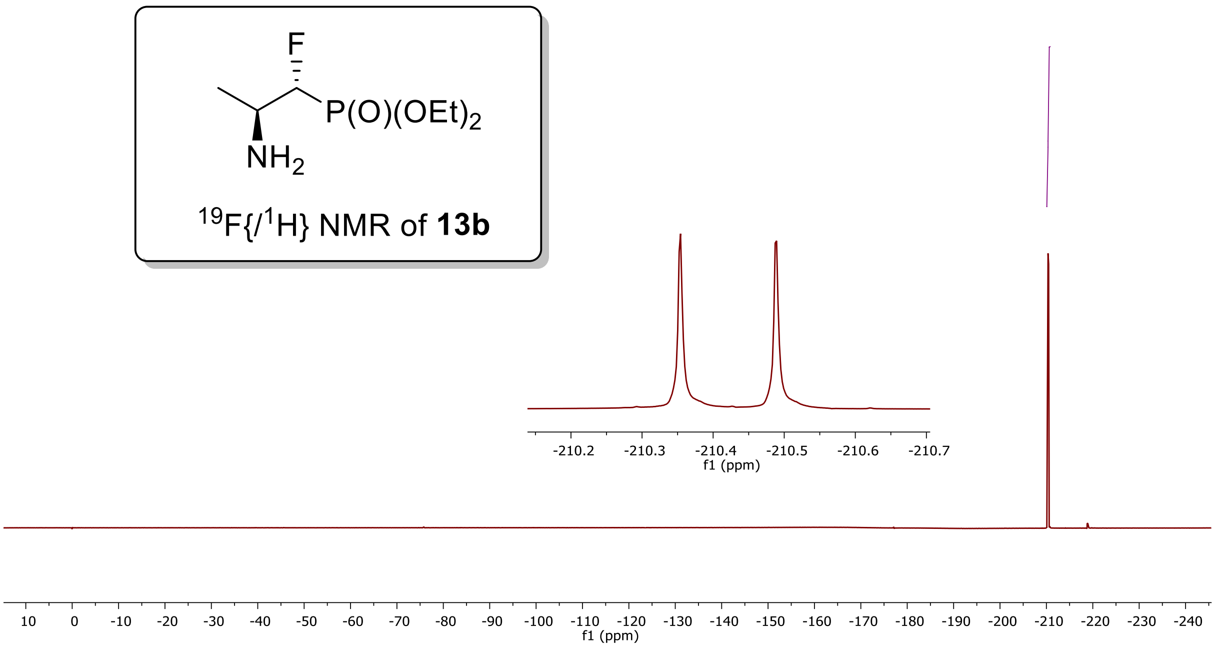

F
P(O)(OEt)2
NH2
19F{/1H} NMR of 13b
-210.2 -210.3 -210.4 -210.5 -210.6 -210.7
f1 (ppm)
10 0 -10 -20 -30 -40 -50 -60 -70 -80 -90 -100 -110 -120 -130 -140 -150 -160 -170 -180 -190 -200 -210 -220 -230 -240
f1 (ppm)

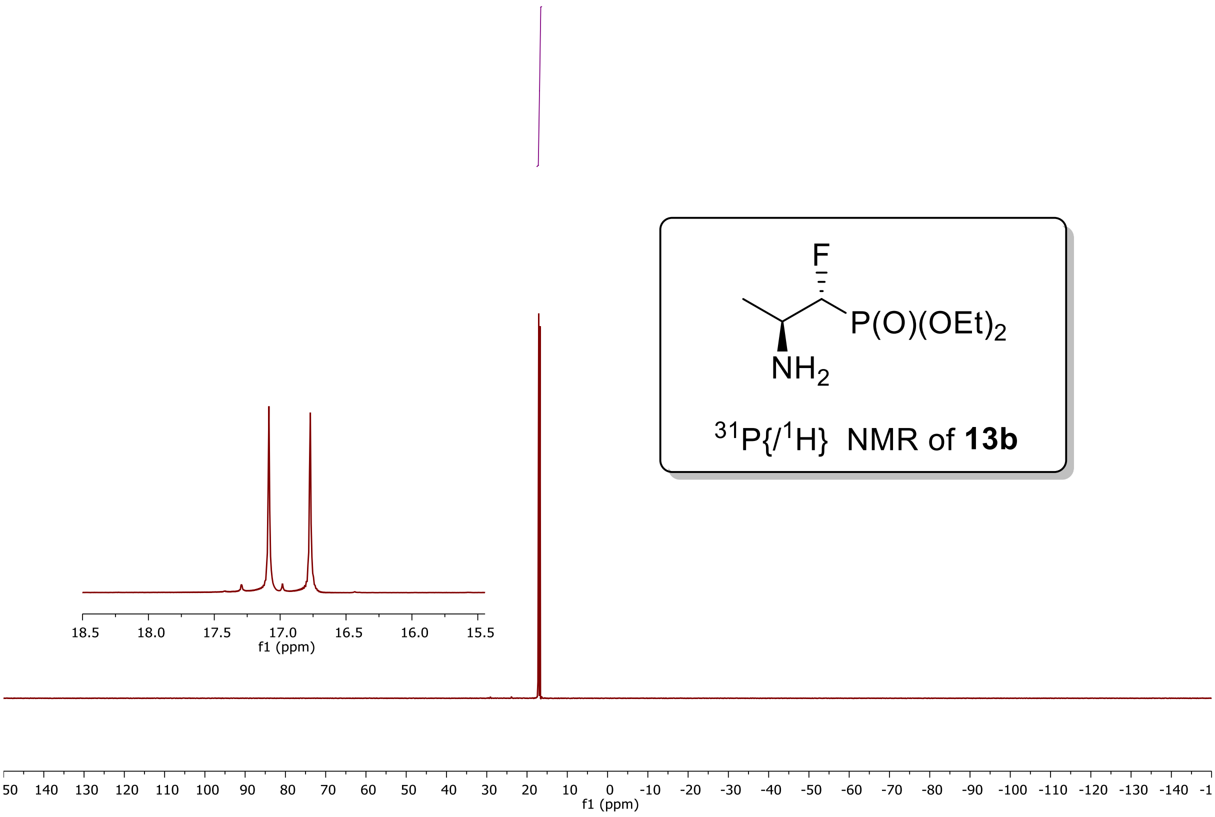

F
P(O)(OEt)2
NH2
31P{/1H} NMR of 13b
18.5 18.0 17.5 17.0 16.5 16.0 15.5
f1 (ppm)
140 130 120 110 100 90 80 70 60 50 40 30 20 10 0 -10 -20 -30 -40 -50 -60 -70 -80 -90 -100 -110 -120 -130 -140
f1 (ppm)

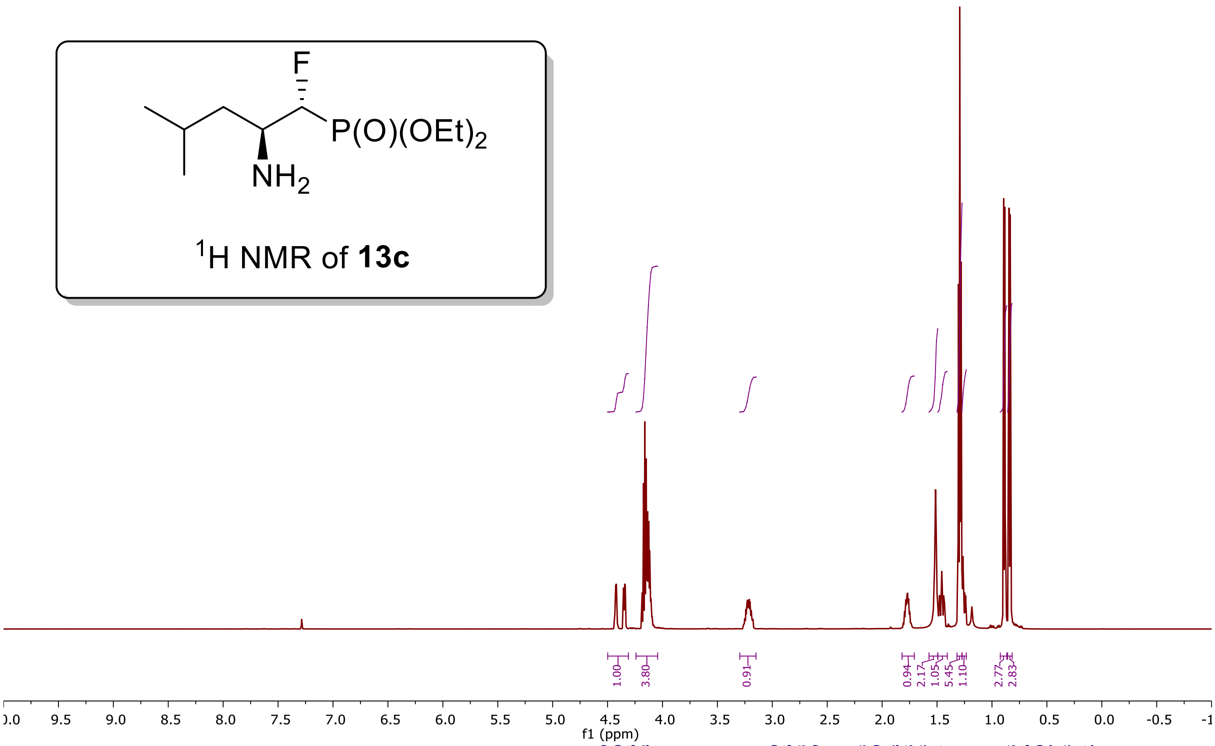
F
P(O)(OEt)2
NH2
1H NMR of 13c
1.00
3.80
0.91
0.94
2.17
1.05
5.45
1.10
2.77
2.83
f1 (ppm)

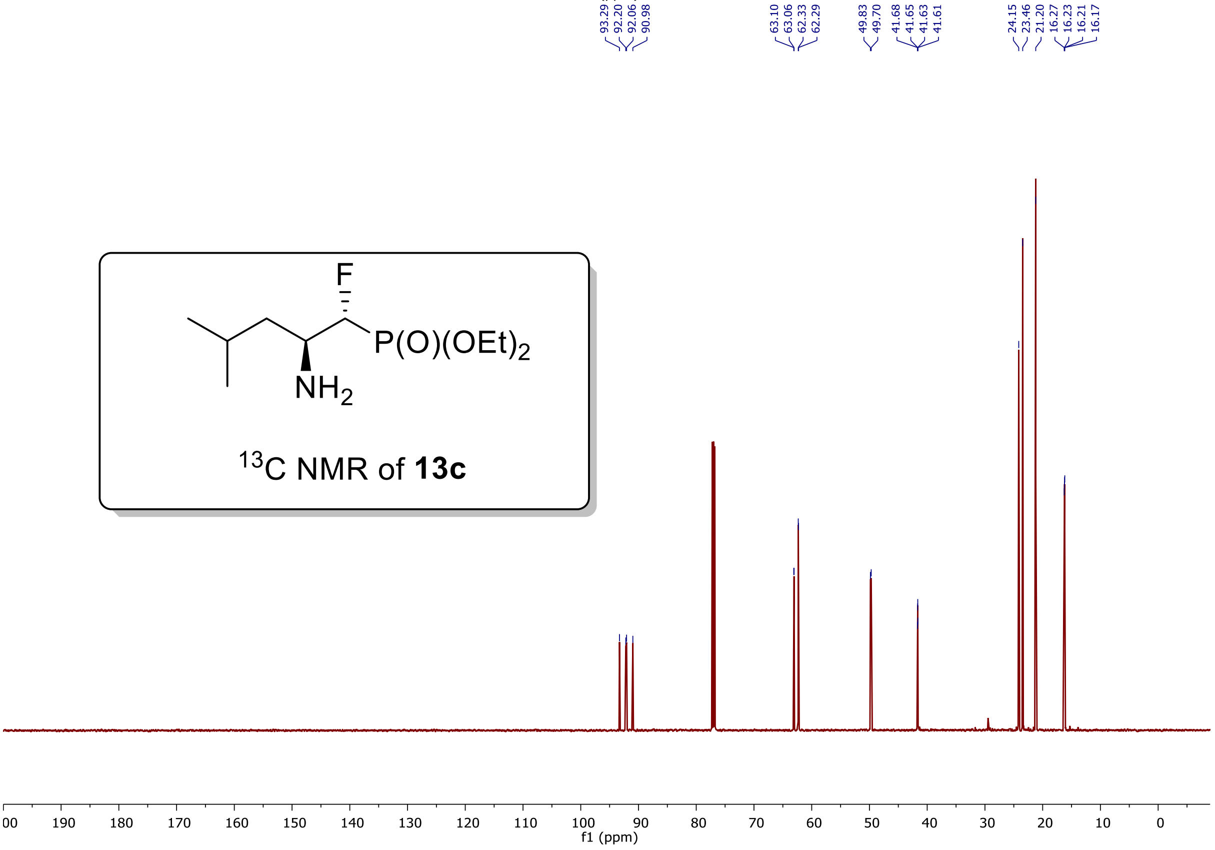
93.29
92.20
92.06
90.98
63.10
63.06
62.33
62.29
49.83
49.70
41.68
41.65
41.63
41.61
24.15
23.46
21.20
16.27
16.23
16.21
16.17
F
P(O)(OEt)2
NH2
13C NMR of 13c
f1 (ppm)

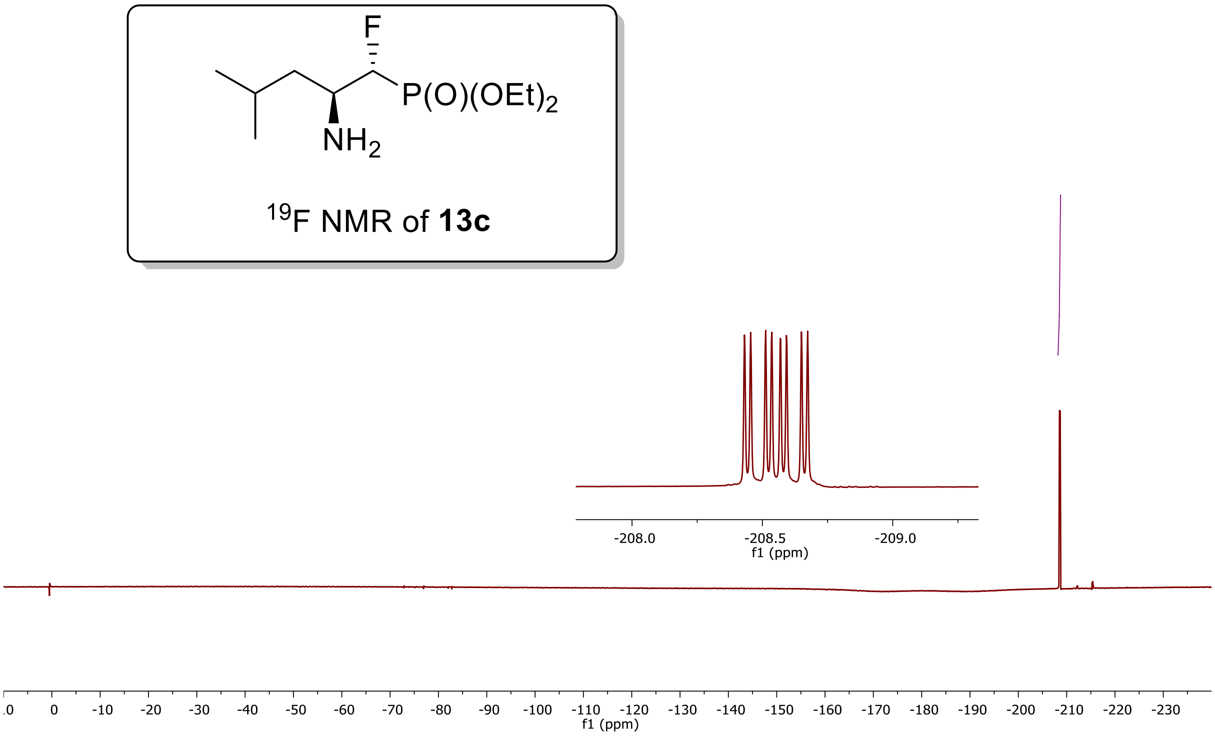

F
P(O)(OEt)2
NH2
19F NMR of 13c
-208.0
-208.5
-209.0
f1 (ppm)
0
-10
-20
-30
-40
-50
-60
-70
-80
-90
-100
-110
-120
-130
-140
-150
-160
-170
-180
-190
-200
-210
-220
-230
f1 (ppm)

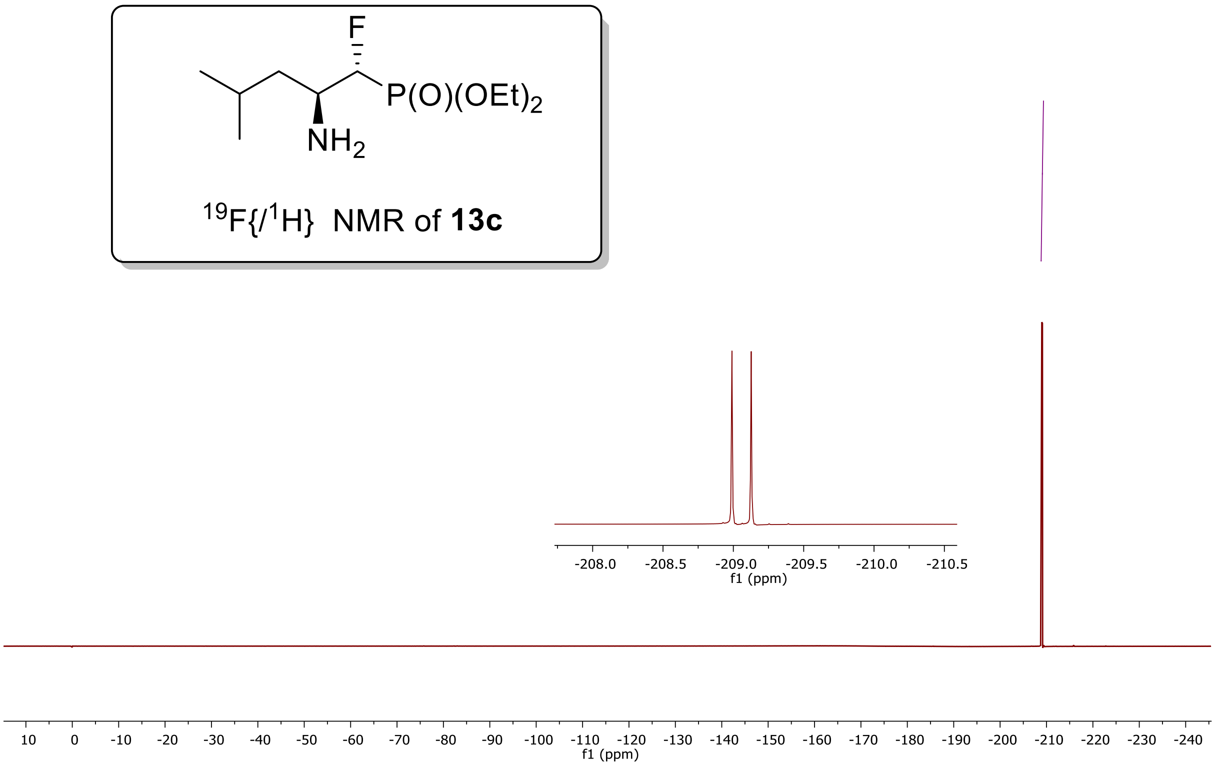

F
P(O)(OEt)2
NH2
19F{/1H} NMR of 13c
-208.0
-208.5
-209.0
-209.5
-210.0
-210.5
f1 (ppm)
10
0
-10
-20
-30
-40
-50
-60
-70
-80
-90
-100
-110
-120
-130
-140
-150
-160
-170
-180
-190
-200
-210
-220
-230
-240
f1 (ppm)

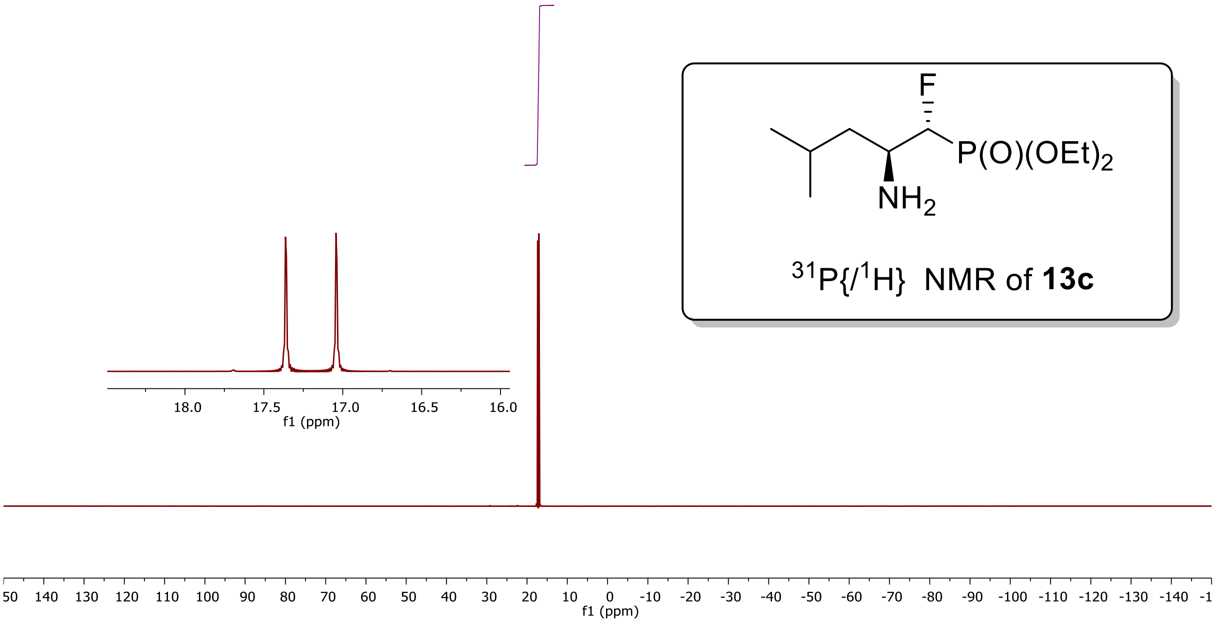
F
P(O)(OEt)2
NH2
31P{/1H} NMR of 13c
18.0
17.5
17.0
16.5
16.0
f1 (ppm)
f1 (ppm)

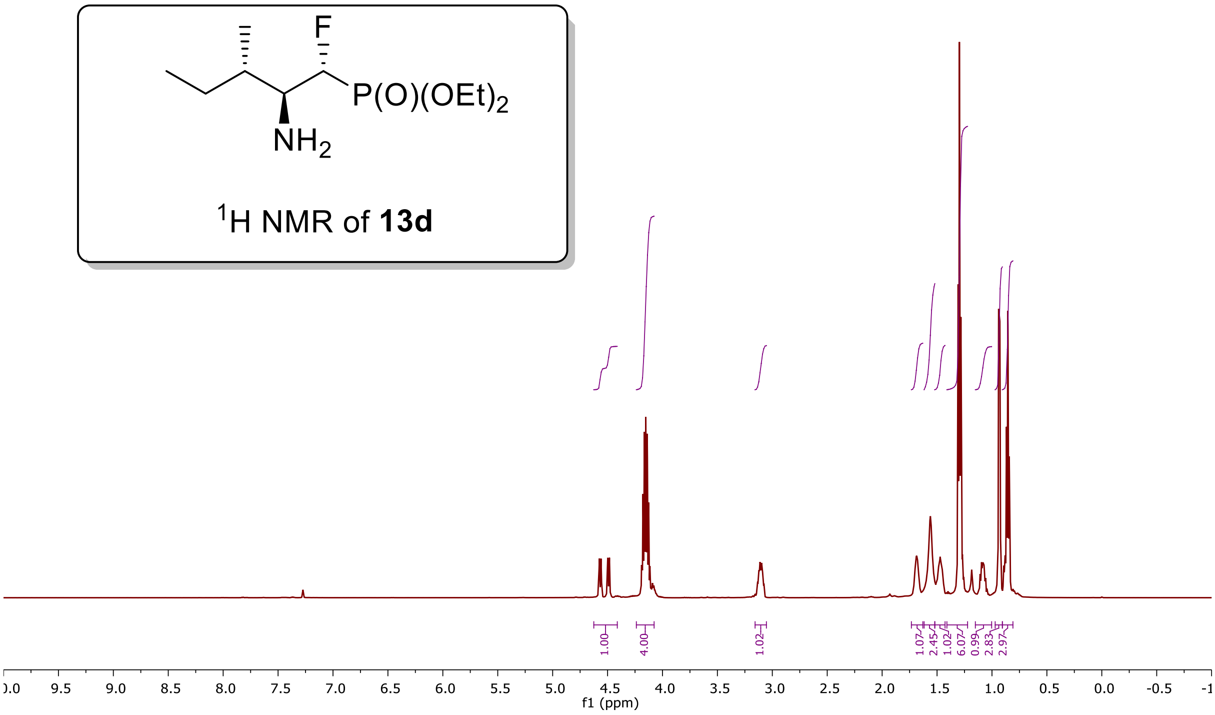
F
P(O)(OEt)2
NH2
1H NMR of 13d
1.00
4.00
1.02
1.07
2.45
1.02
6.07
0.99
2.83
2.97
f1 (ppm)

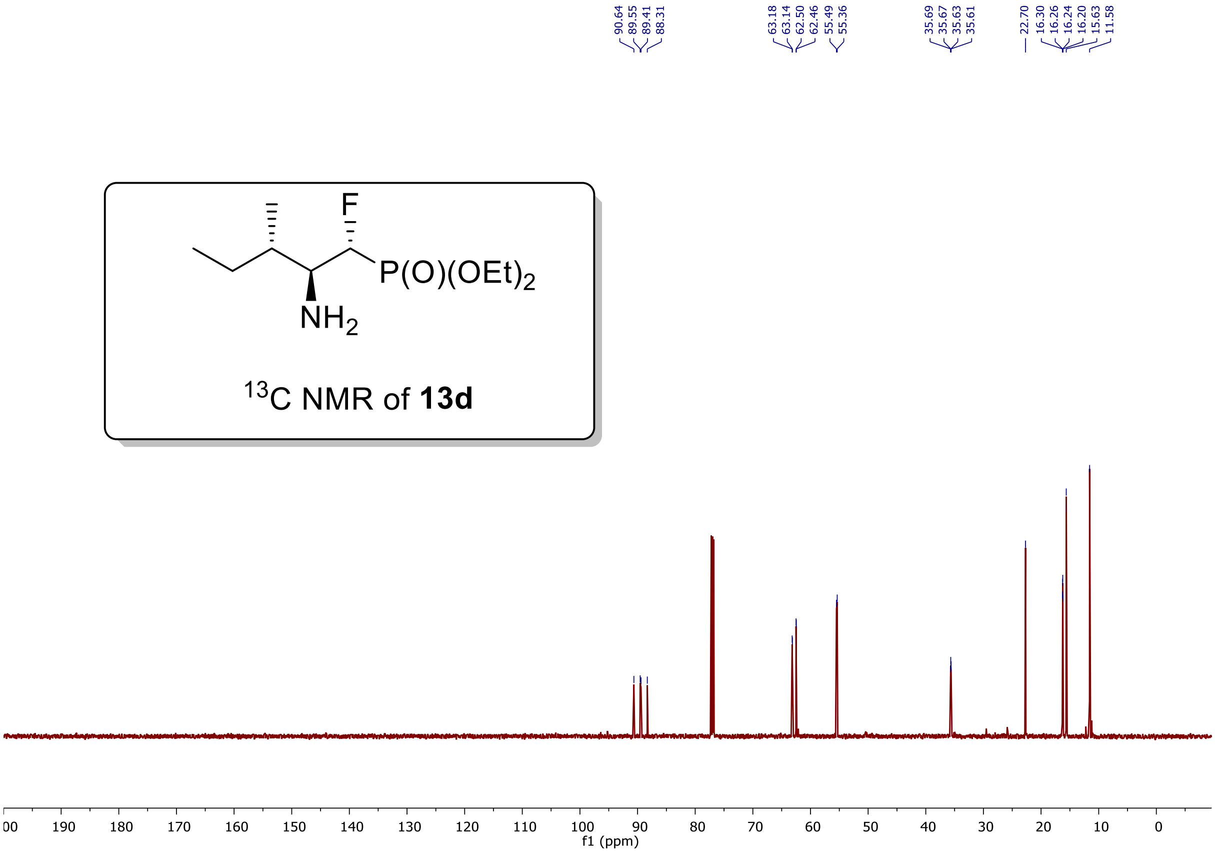
90.64
89.55
89.41
88.31
63.18
63.14
62.50
62.46
55.49
55.36
35.69
35.67
35.63
35.61
22.70
16.30
16.26
16.24
16.20
15.63
11.58
F
P(O)(OEt)2
NH2
13C NMR of 13d
f1 (ppm)

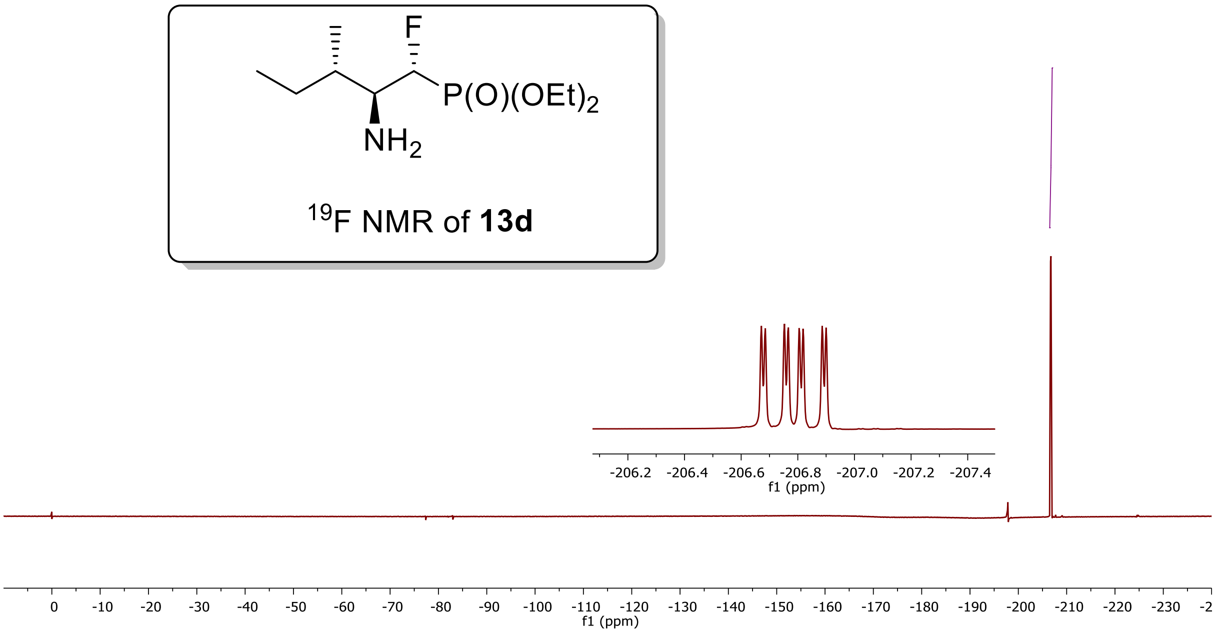
F
P(O)(OEt)2
NH2
19F NMR of 13d
-206.2
-206.4
-206.6
-206.8
-207.0
-207.2
-207.4
f1 (ppm)

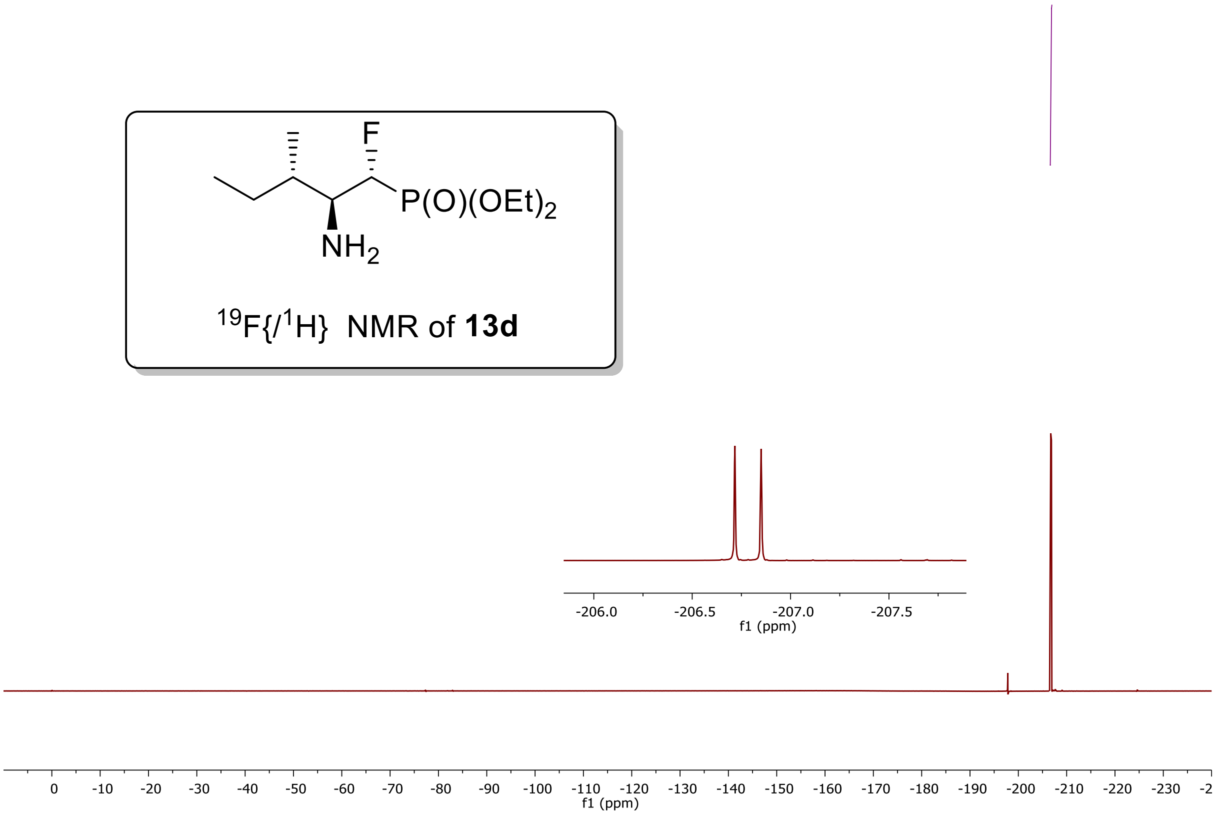
F
P(O)(OEt)$_2$
NH$_2$
^{19}F{/^{1}H} NMR of **13d**
-206.0
-206.5
-207.0
-207.5
f1 (ppm)
0
-10
-20
-30
-40
-50
-60
-70
-80
-90
-100
-110
-120
-130
-140
-150
-160
-170
-180
-190
-200
-210
-220
-230
f1 (ppm)

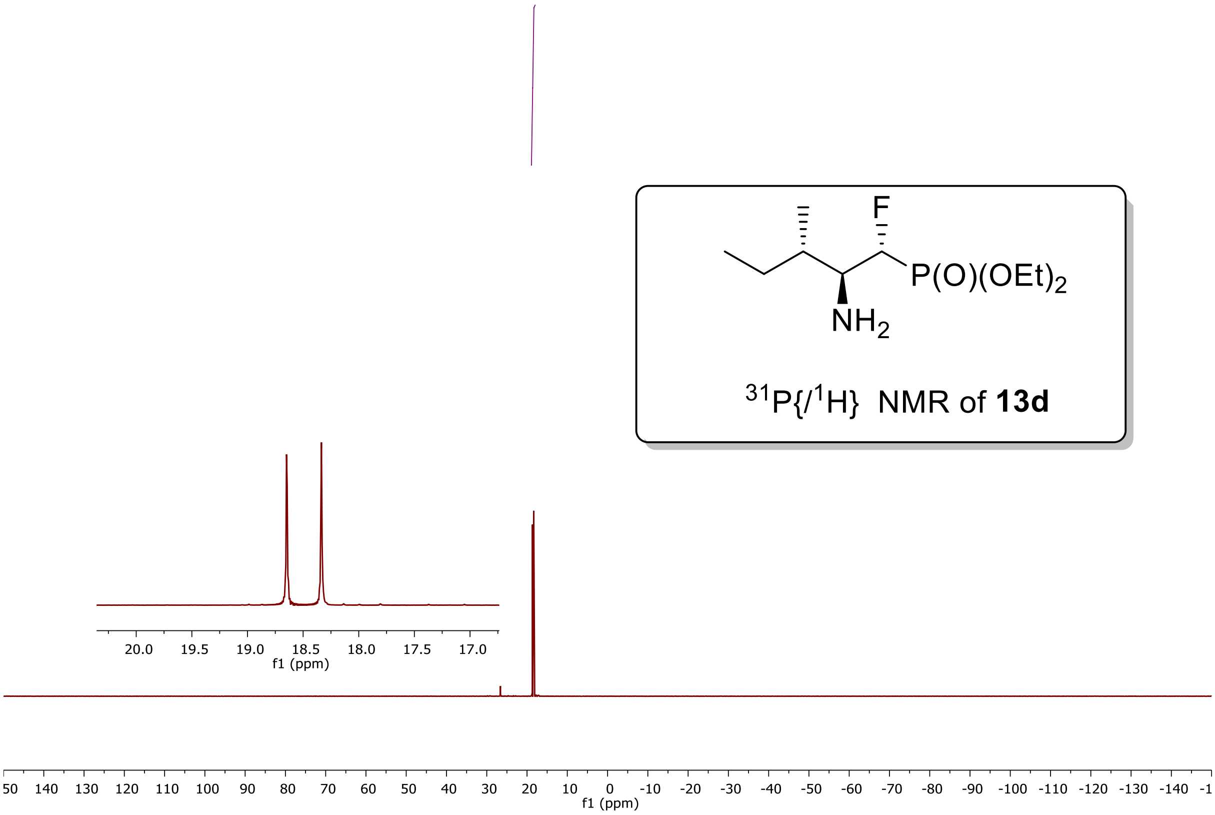
F
P(O)(OEt)$_2$
NH$_2$
^{31}P{/^{1}H} NMR of **13d**
20.0
19.5
19.0
18.5
18.0
17.5
17.0
f1 (ppm)
140
130
120
110
100
90
80
70
60
50
40
30
20
10
0
-10
-20
-30
-40
-50
-60
-70
-80
-90
-100
-110
-120
-130
-140
f1 (ppm)

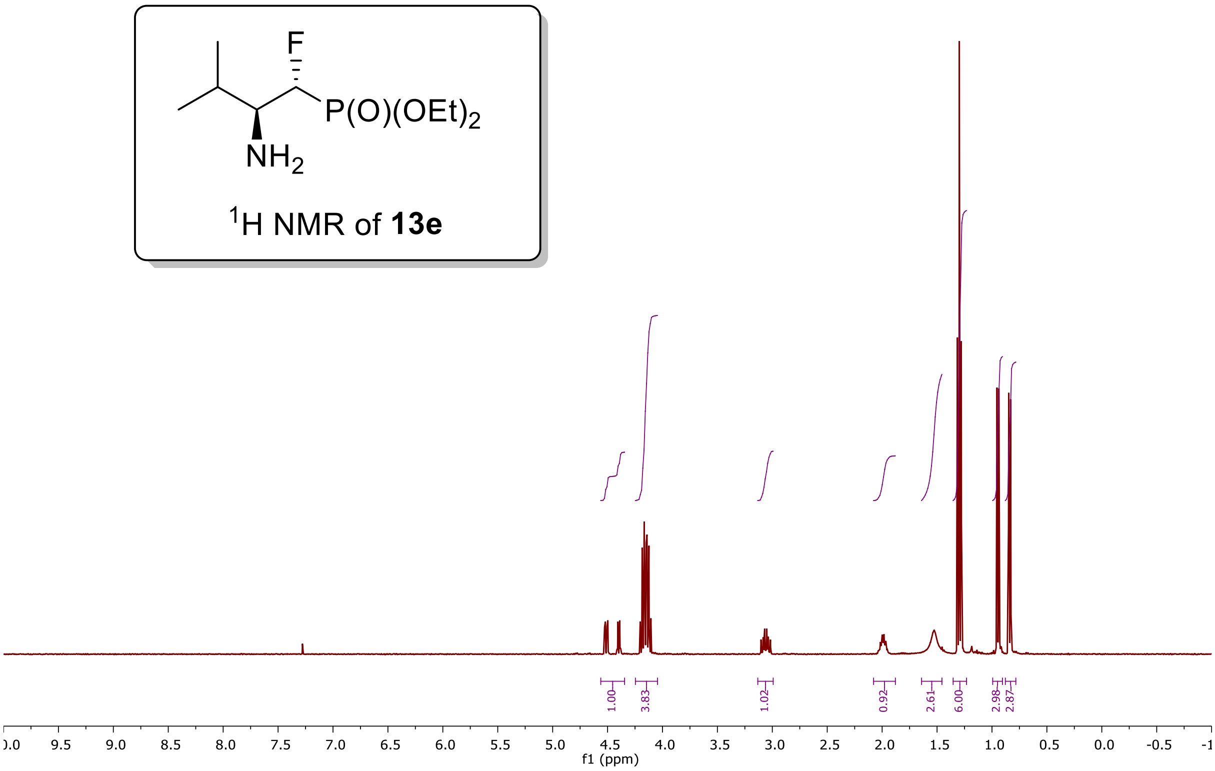
F
P(O)(OEt)2
NH2
1H NMR of 13e
1.00
3.83
1.02
0.92
2.61
6.00
2.98
2.87
f1 (ppm)

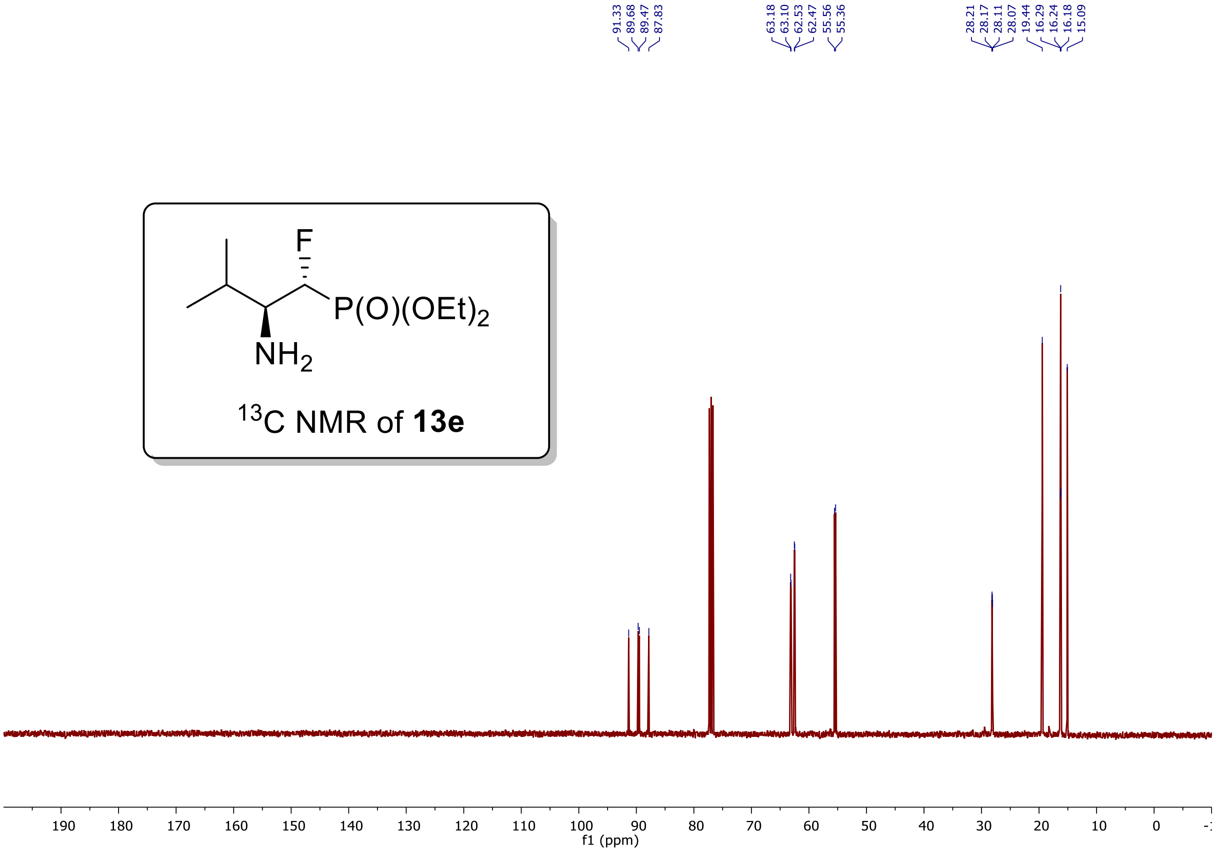
91.33
89.68
89.47
87.83
63.18
63.10
62.53
62.47
55.56
55.36
28.21
28.17
28.11
28.07
19.44
16.29
16.24
16.18
15.09
F
P(O)(OEt)2
NH2
13C NMR of 13e
f1 (ppm)

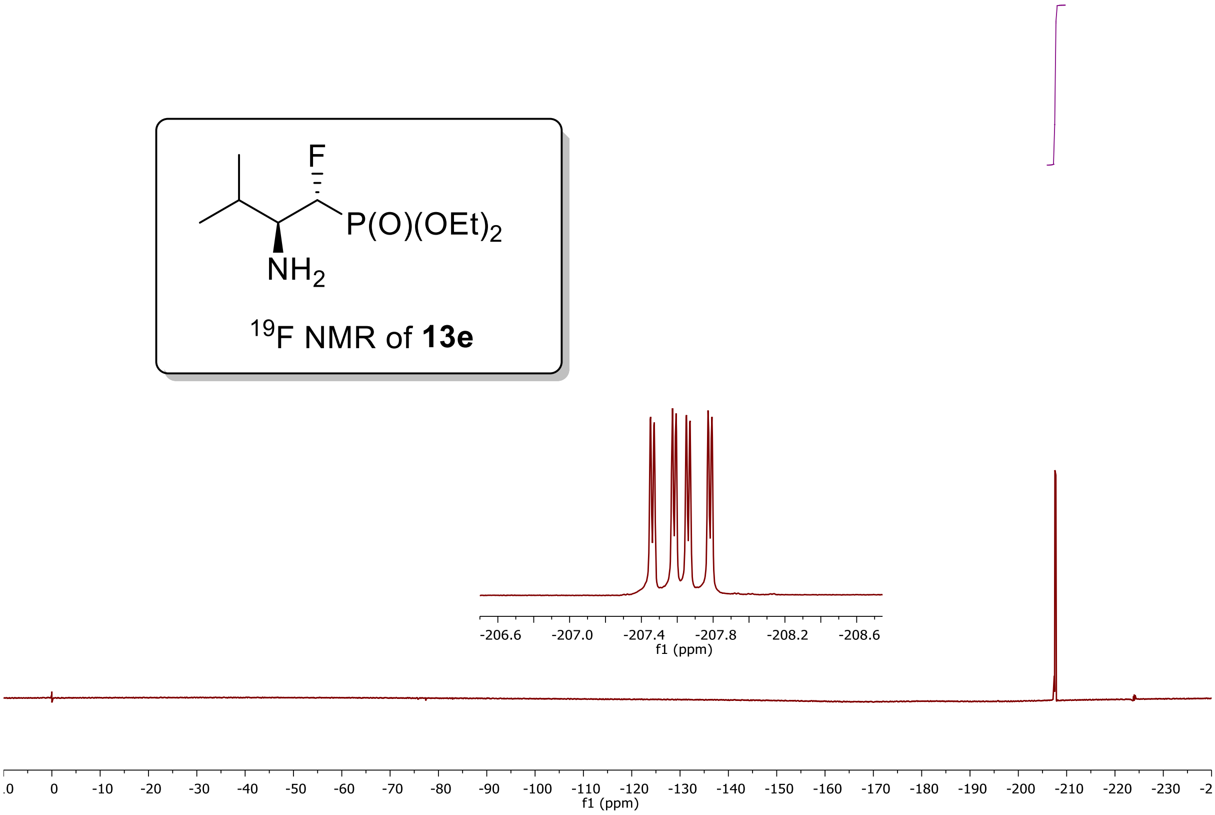
F
P(O)(OEt)2
NH2
19F NMR of 13e
f1 (ppm)
f1 (ppm)

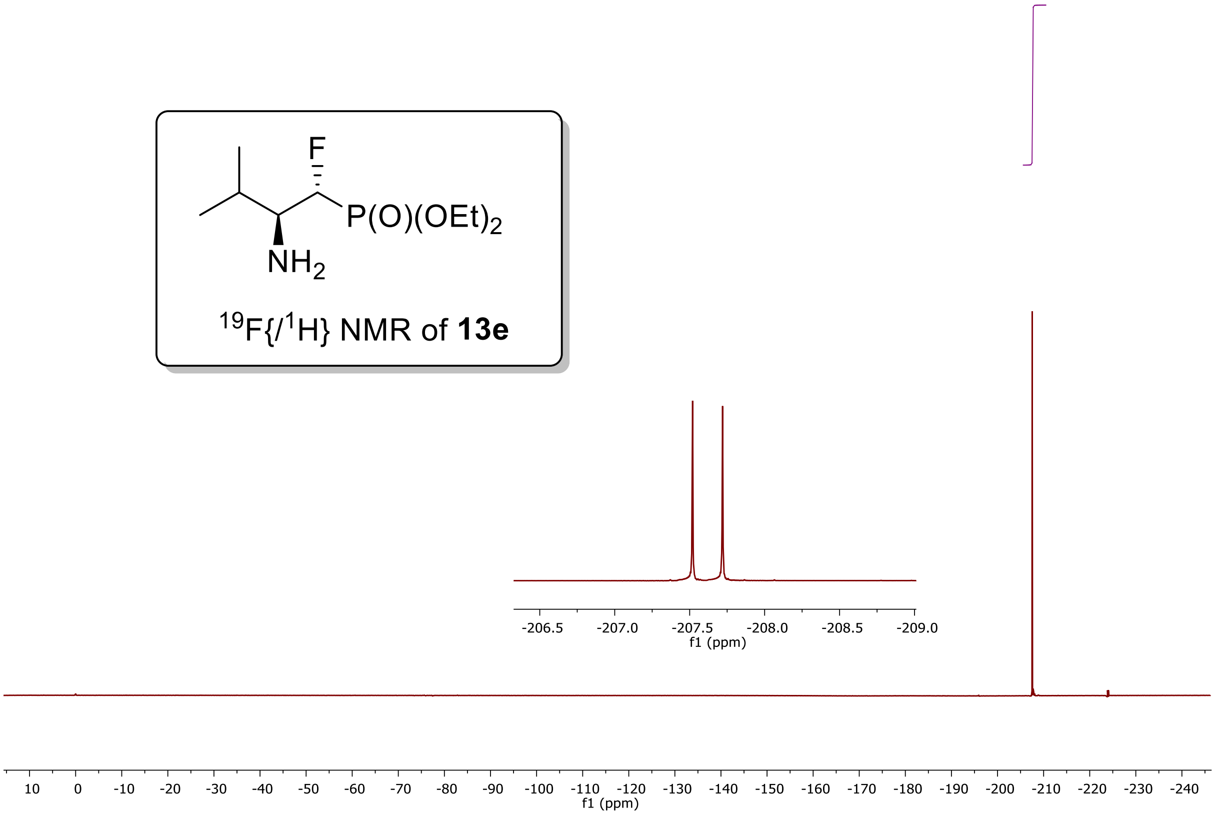
F
P(O)(OEt)2
NH2
19F{/1H} NMR of 13e
f1 (ppm)
f1 (ppm)

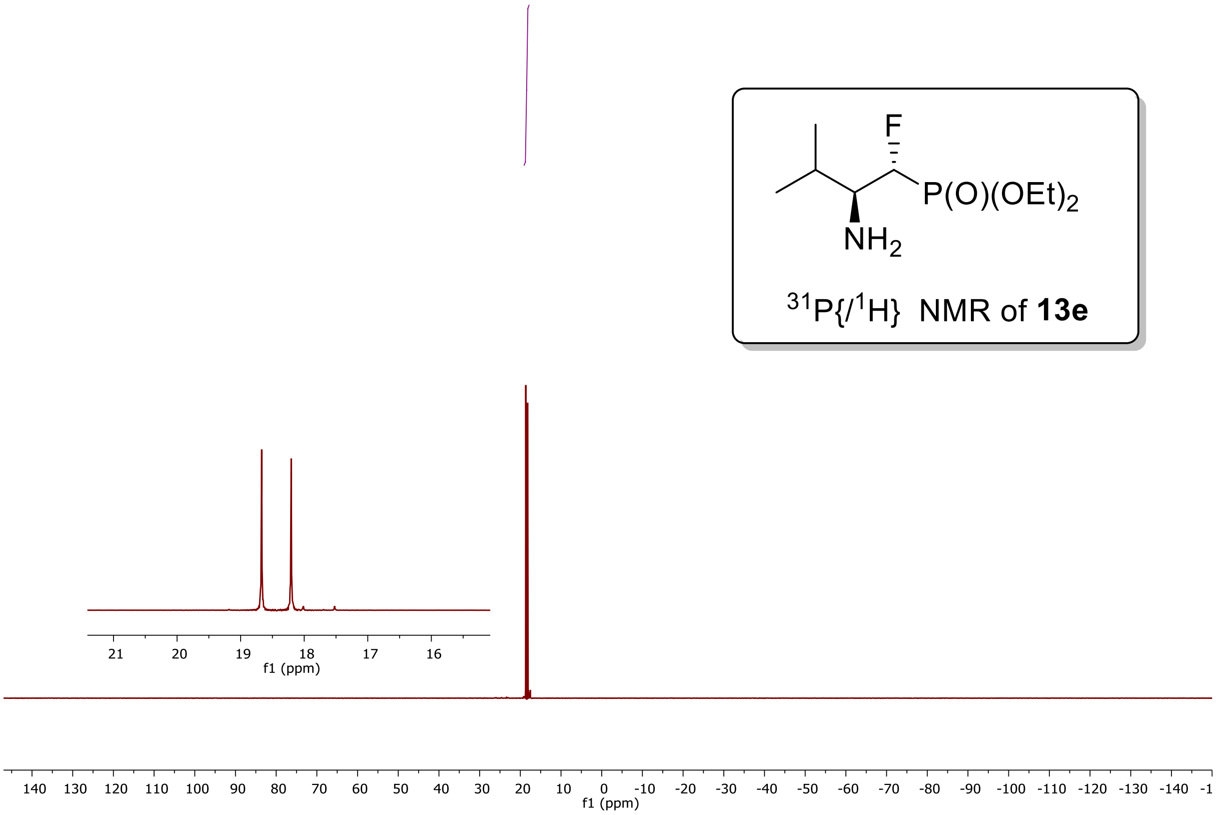

F
P(O)(OEt)2
NH2
31P{/1H} NMR of 13e
21
20
19
18
17
16
f1 (ppm)
140
130
120
110
100
90
80
70
60
50
40
30
20
10
0
-10
-20
-30
-40
-50
-60
-70
-80
-90
-100
-110
-120
-130
-140
f1 (ppm)

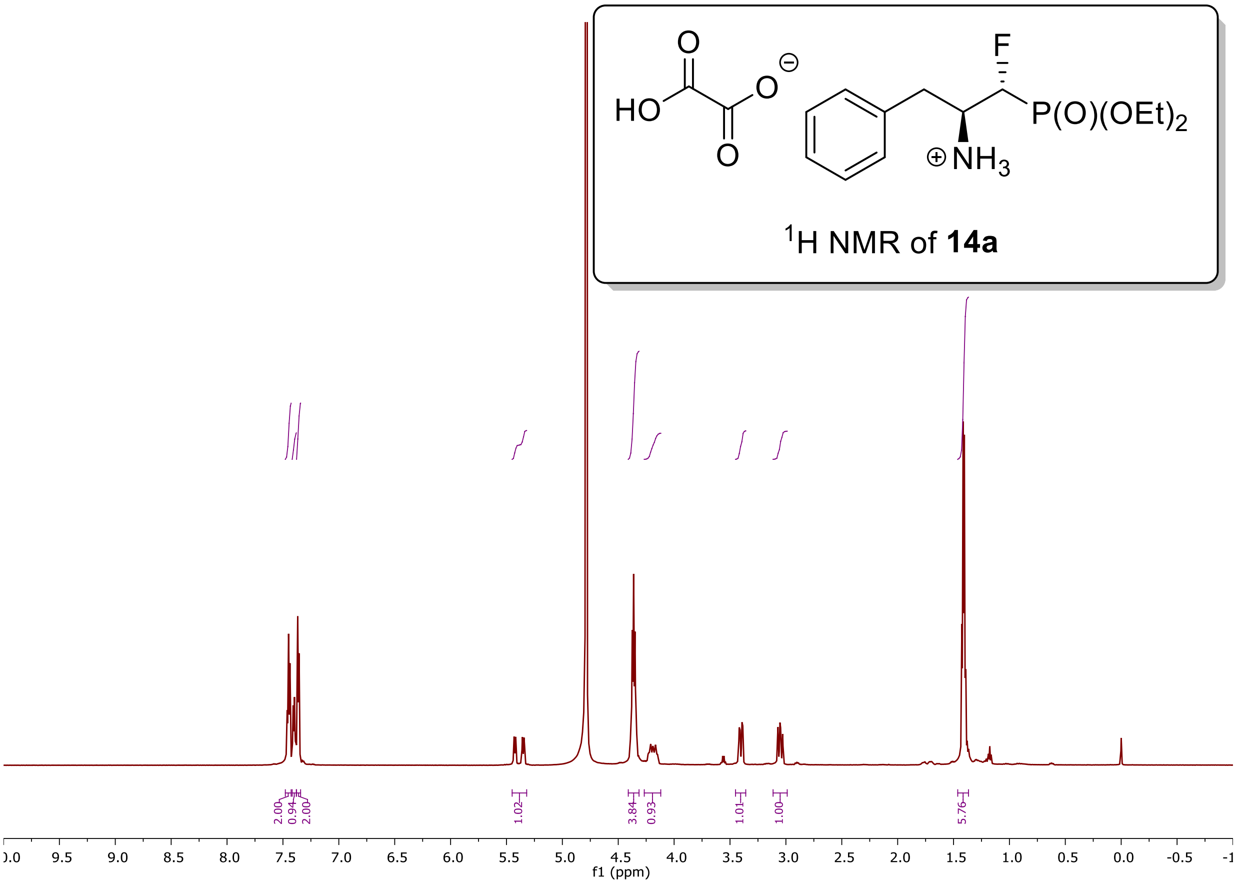

O
HO
O
O
F
P(O)(OEt)2
NH3
1H NMR of 14a
2.00
0.94
2.00
1.02
3.84
0.93
1.01
1.00
5.76
9.5
9.0
8.5
8.0
7.5
7.0
6.5
6.0
5.5
5.0
4.5
4.0
3.5
3.0
2.5
2.0
1.5
1.0
0.5
0.0
-0.5
f1 (ppm)

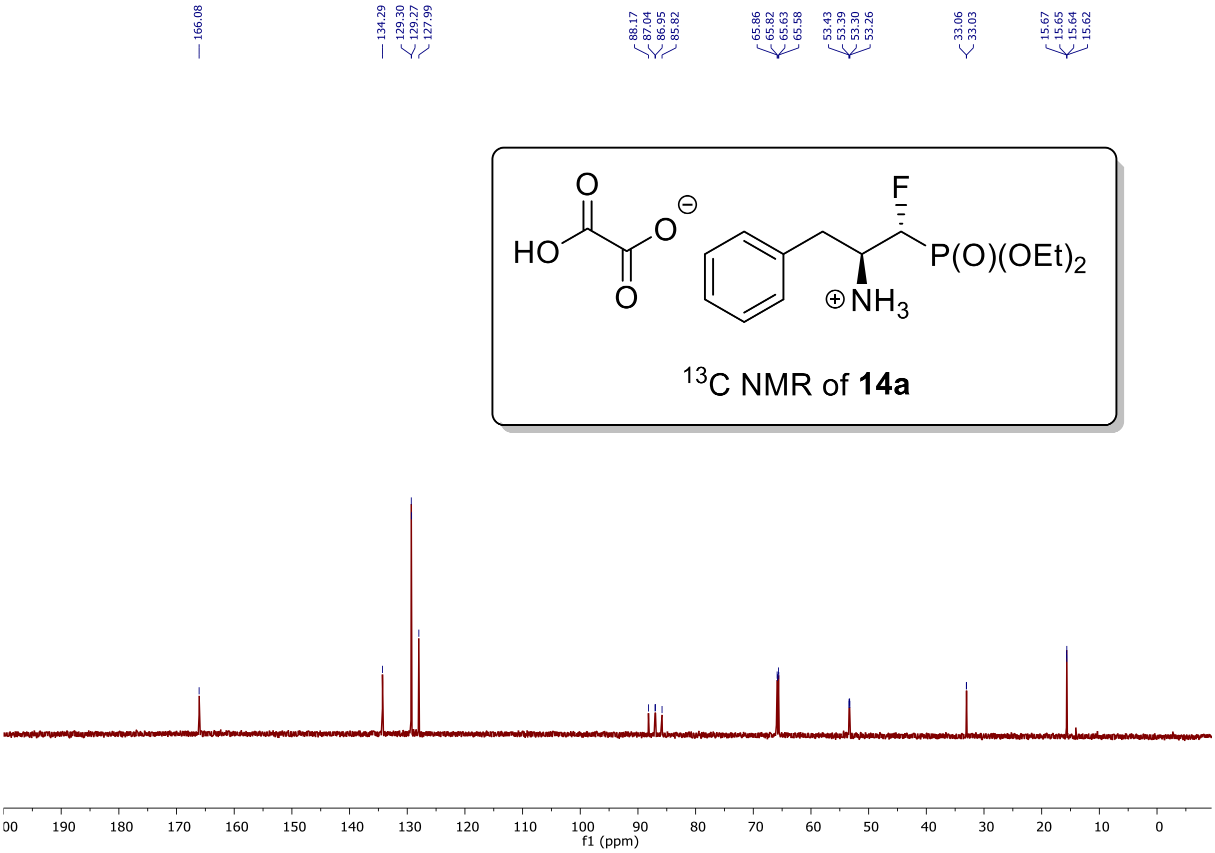
166.08
134.29
129.30
129.27
127.99
88.17
87.04
86.95
85.82
65.86
65.82
65.63
65.58
53.43
53.39
53.30
53.26
33.06
33.03
15.67
15.65
15.64
15.62
HO
O
O
O
F
P(O)(OEt)2
NH3
13C NMR of 14a
f1 (ppm)

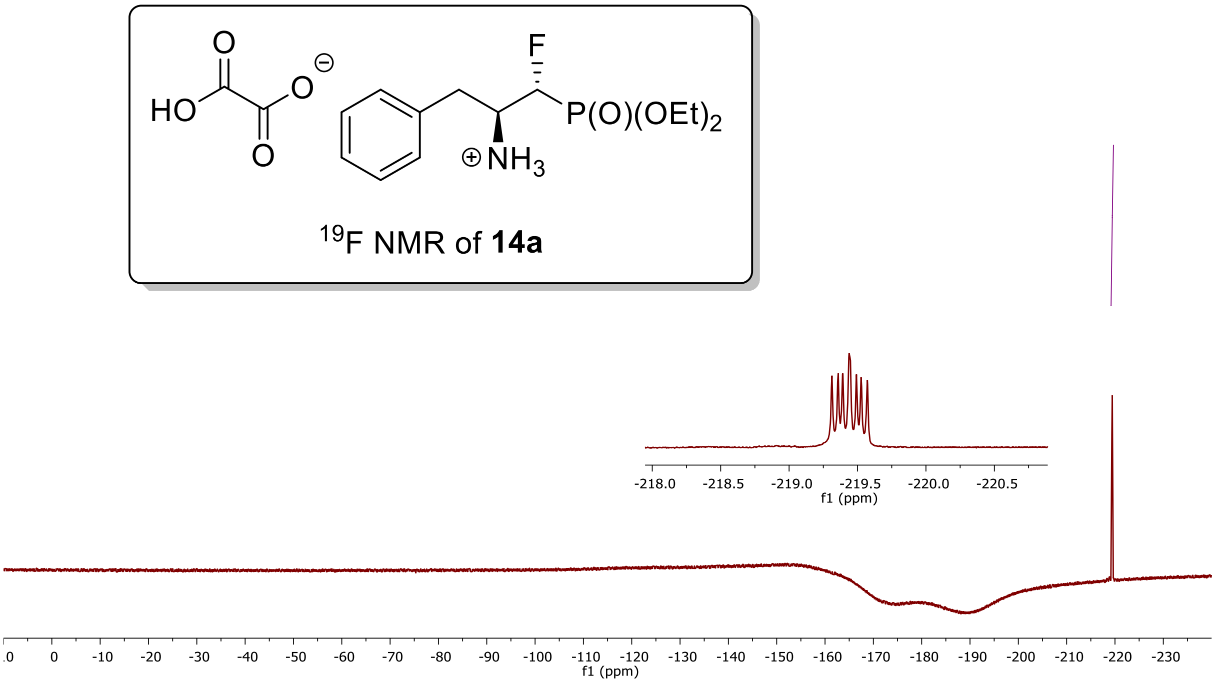
HO
O
O
O
F
P(O)(OEt)2
NH3
19F NMR of 14a
-218.0
-218.5
-219.0
-219.5
-220.0
-220.5
f1 (ppm)

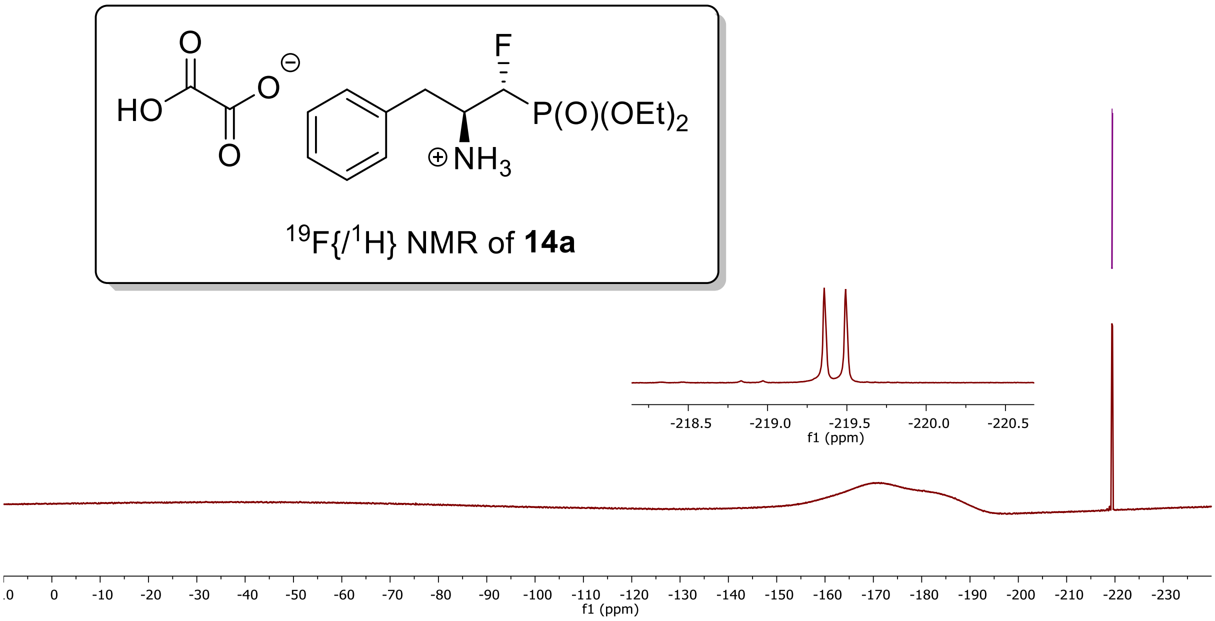
HO
O
O
O
F
P(O)(OEt)2
NH3
19F{/1H} NMR of 14a
-218.5 -219.0 -219.5 -220.0 -220.5
f1 (ppm)
0 -10 -20 -30 -40 -50 -60 -70 -80 -90 -100 -110 -120 -130 -140 -150 -160 -170 -180 -190 -200 -210 -220 -230
f1 (ppm)

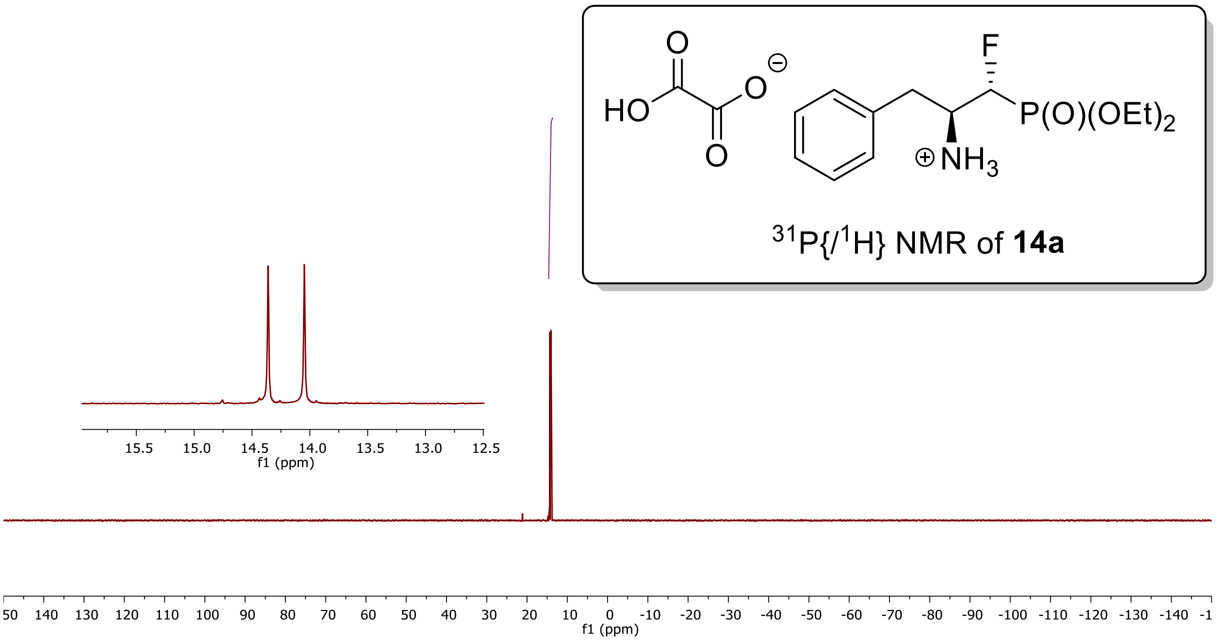
HO
O
O
O
F
P(O)(OEt)2
NH3
31P{/1H} NMR of 14a
15.5 15.0 14.5 14.0 13.5 13.0 12.5
f1 (ppm)
140 130 120 110 100 90 80 70 60 50 40 30 20 10 0 -10 -20 -30 -40 -50 -60 -70 -80 -90 -100 -110 -120 -130 -140
f1 (ppm)

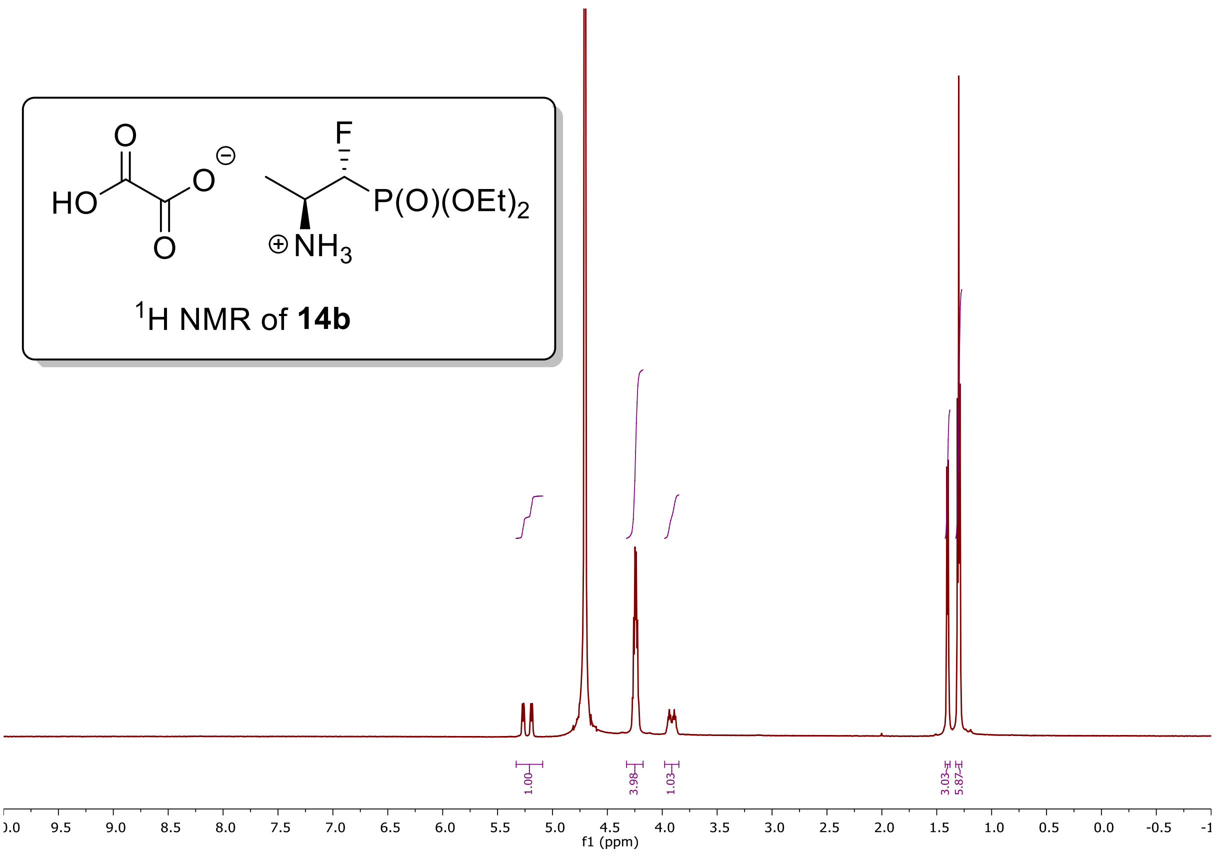
^{1}H NMR of **14b**
HO
O
P(O)(OEt)2
F
NH3
1.00
3.98
1.03
3.03
5.87
f1 (ppm)

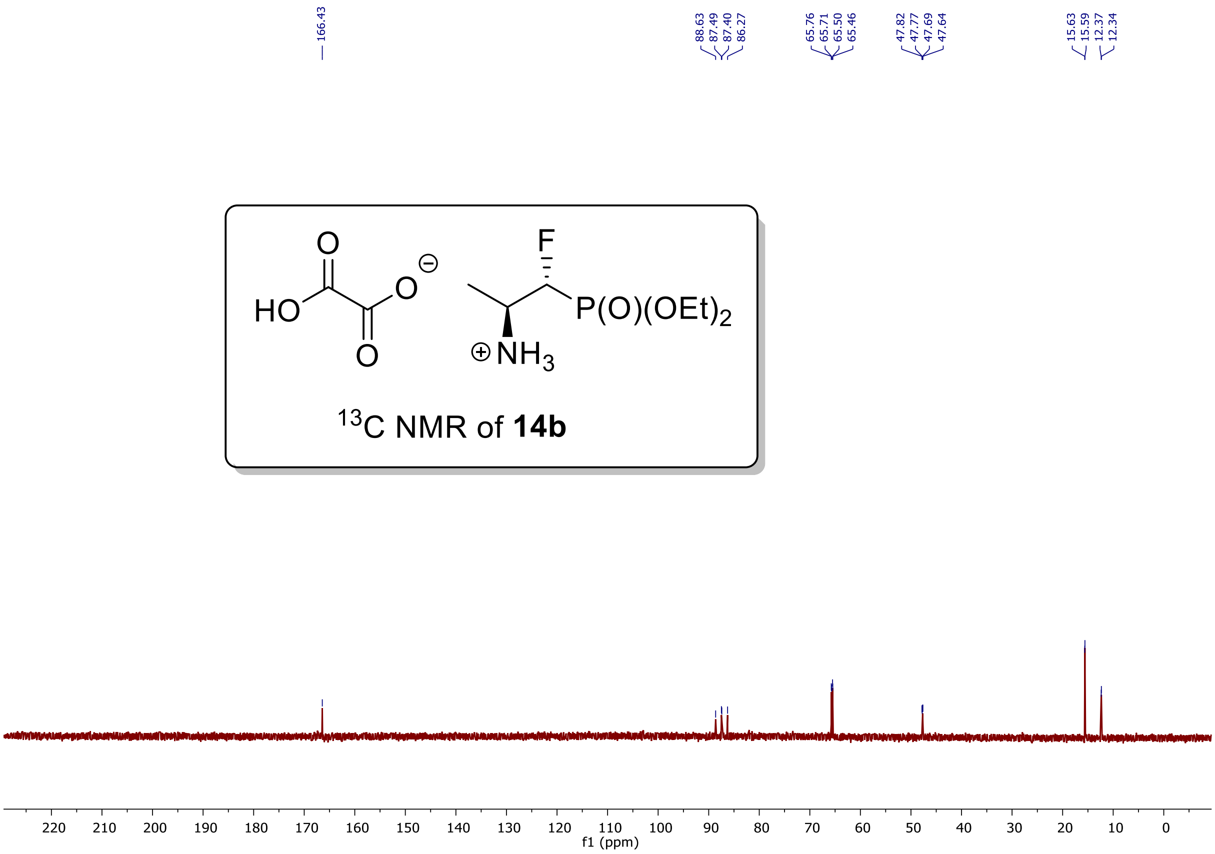
166.43
88.63
87.49
87.40
86.27
65.76
65.71
65.50
65.46
47.82
47.77
47.69
47.64
15.63
15.59
12.37
12.34
^{13}C NMR of **14b**
HO
O
P(O)(OEt)2
F
NH3
f1 (ppm)

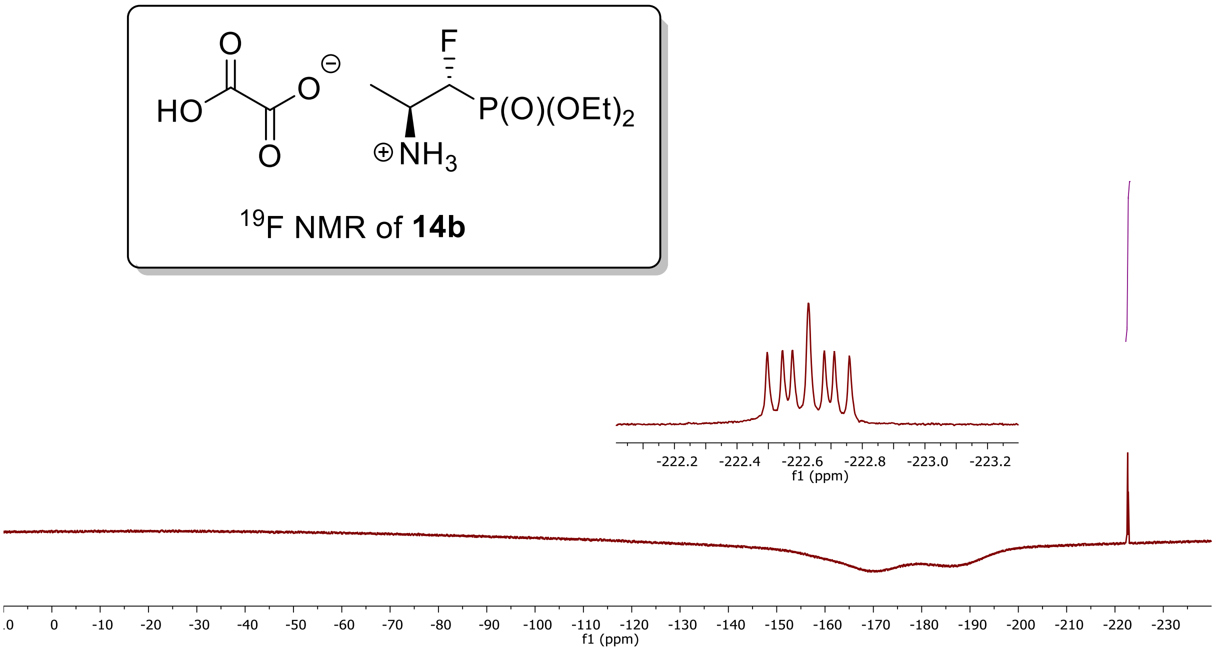

^{19}F NMR of **14b**

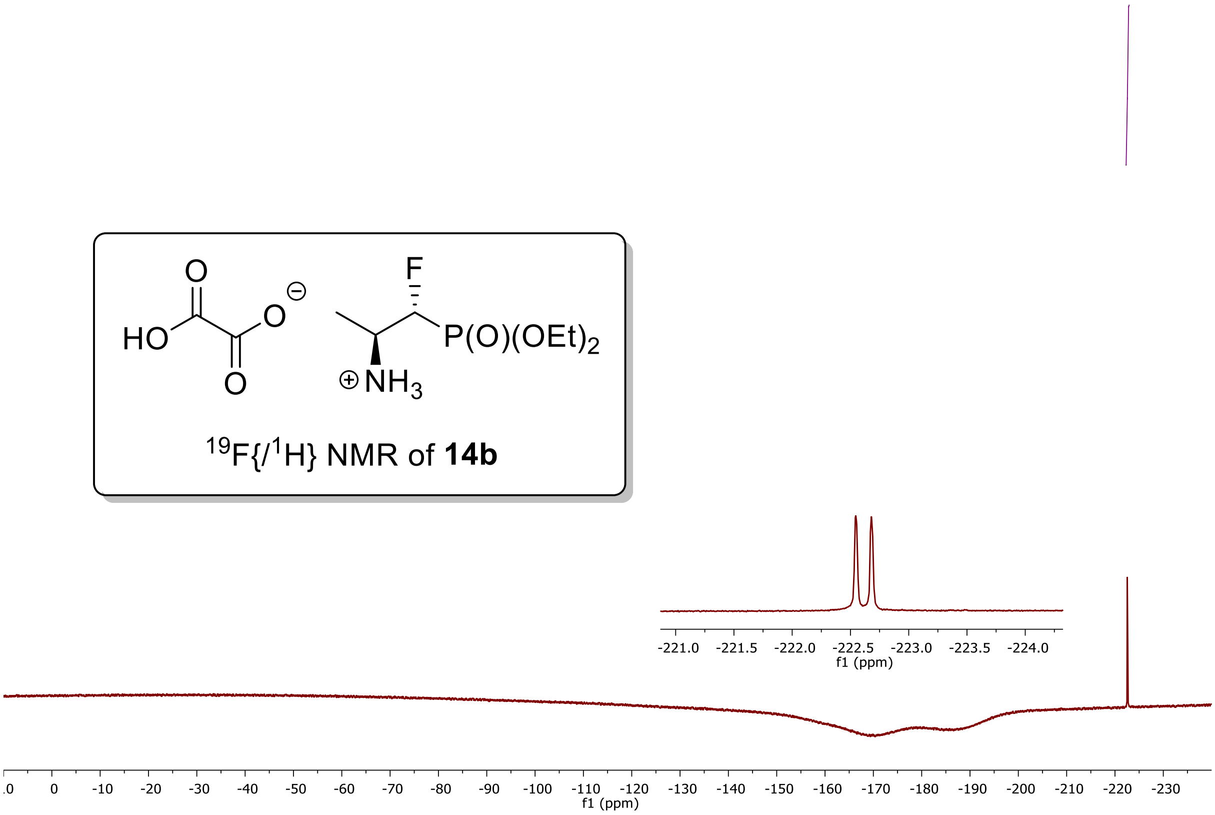

^{19}F{/^{1}H} NMR of **14b**

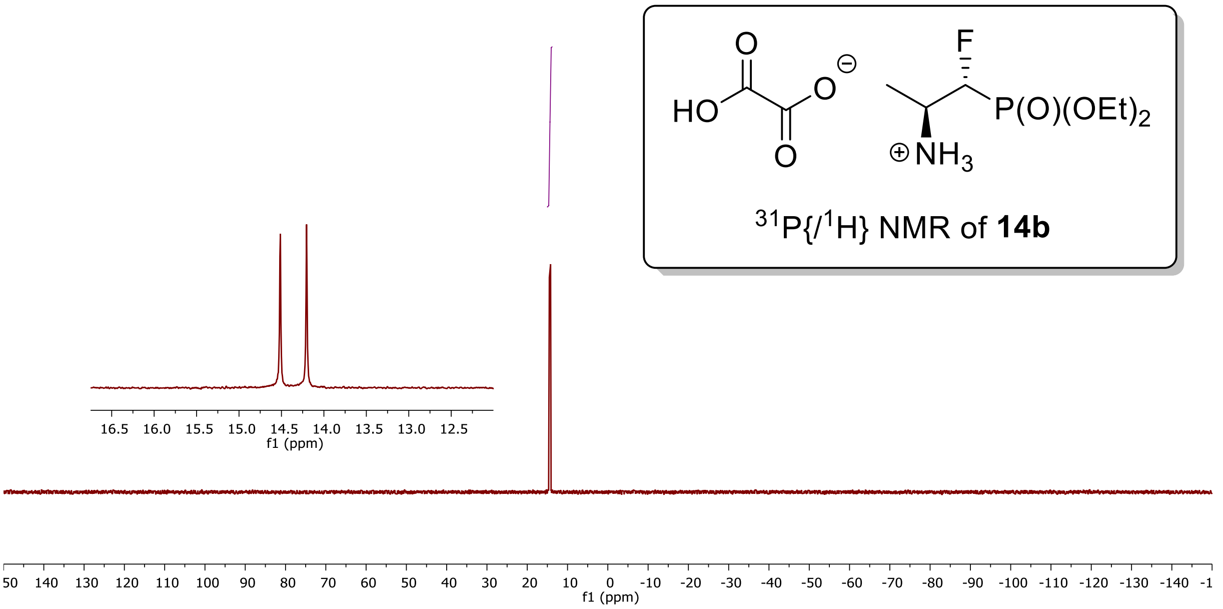
HO
O
O
O
F
P(O)(OEt)$_2$
NH$_3$
^{31}P{/^{1}H} NMR of **14b**
16.5 16.0 15.5 15.0 14.5 14.0 13.5 13.0 12.5
f1 (ppm)
f1 (ppm)

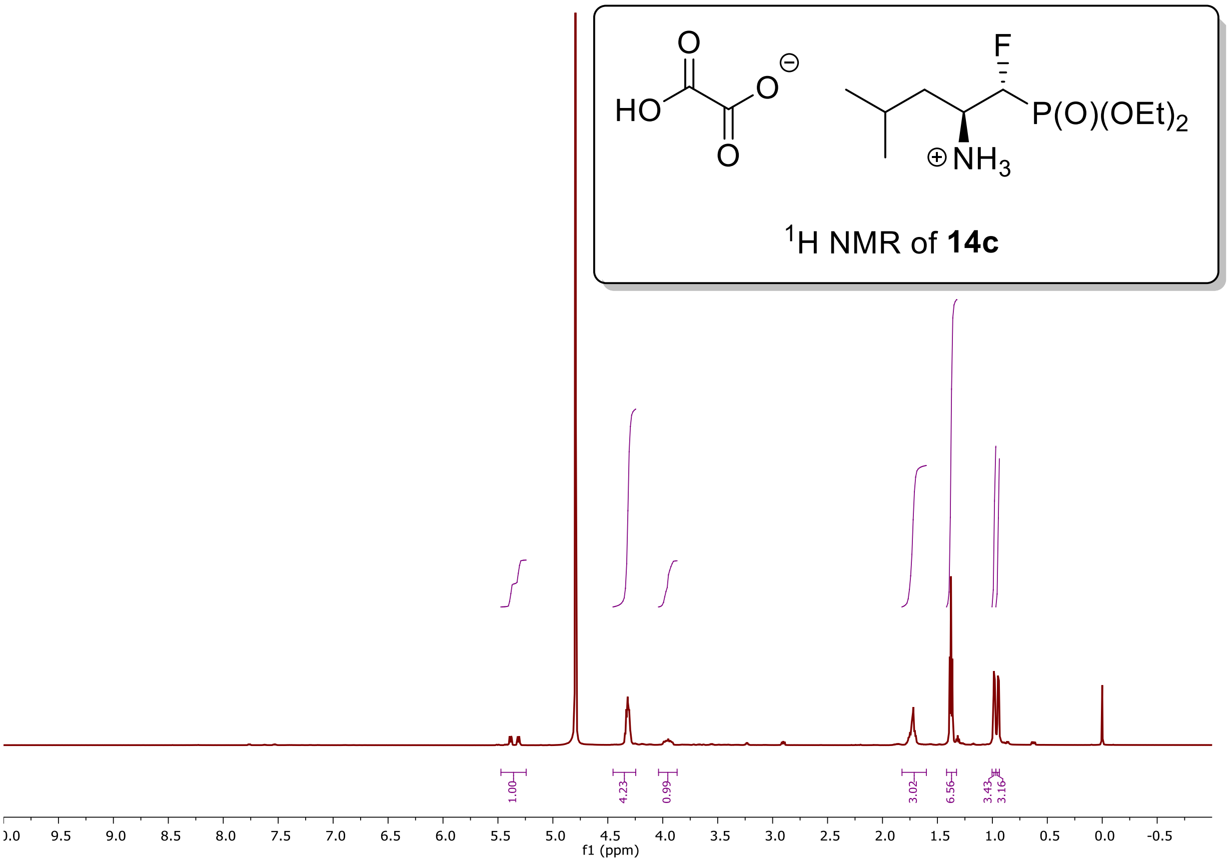
HO
O
O
O
F
P(O)(OEt)$_2$
NH$_3$
^{1}H NMR of **14c**
1.00
4.23
0.99
3.02
6.56
3.43
3.16
f1 (ppm)

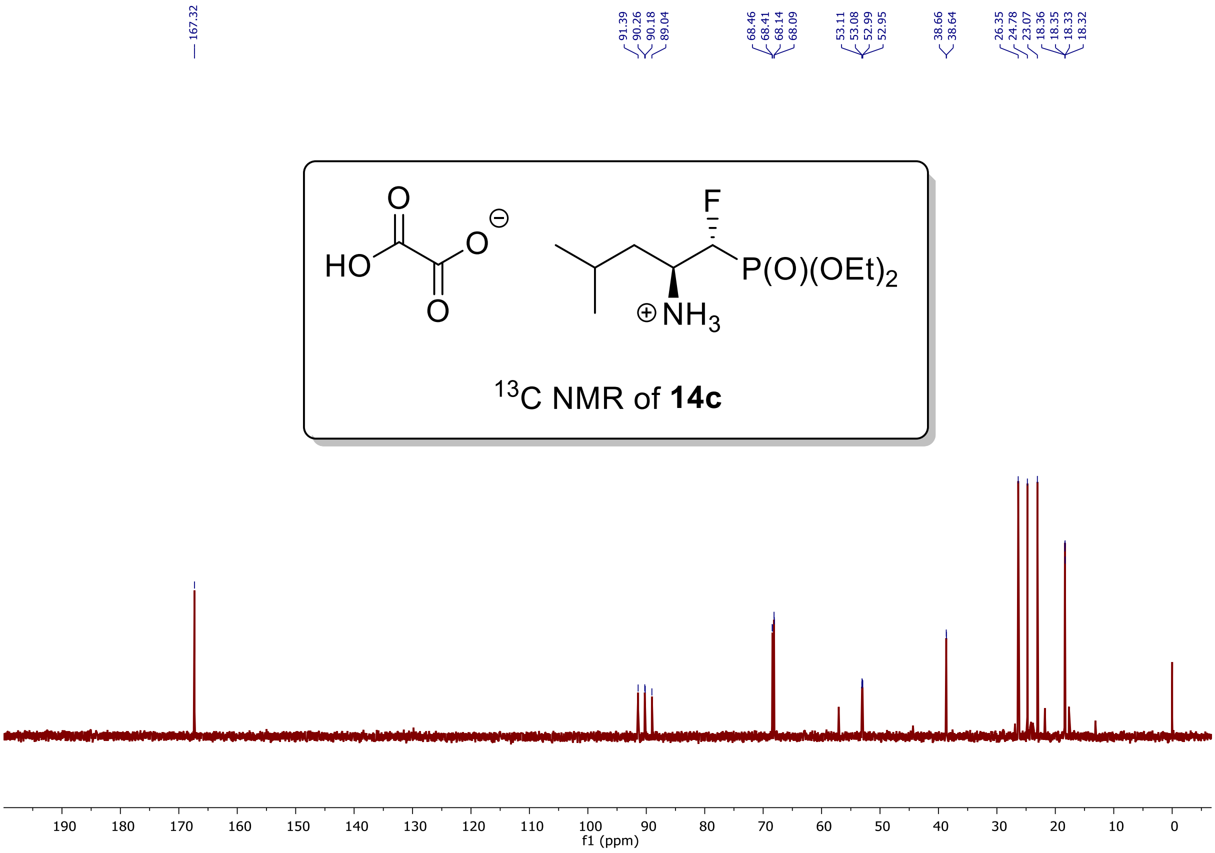
167.32
91.39
90.26
90.18
89.04
68.46
68.41
68.14
68.09
53.11
53.08
52.99
52.95
38.66
38.64
26.35
24.78
23.07
18.36
18.35
18.33
18.32
HO
O
O
O
F
P(O)(OEt)2
NH3
13C NMR of 14c
190 180 170 160 150 140 130 120 110 100 90 80 70 60 50 40 30 20 10 0
f1 (ppm)

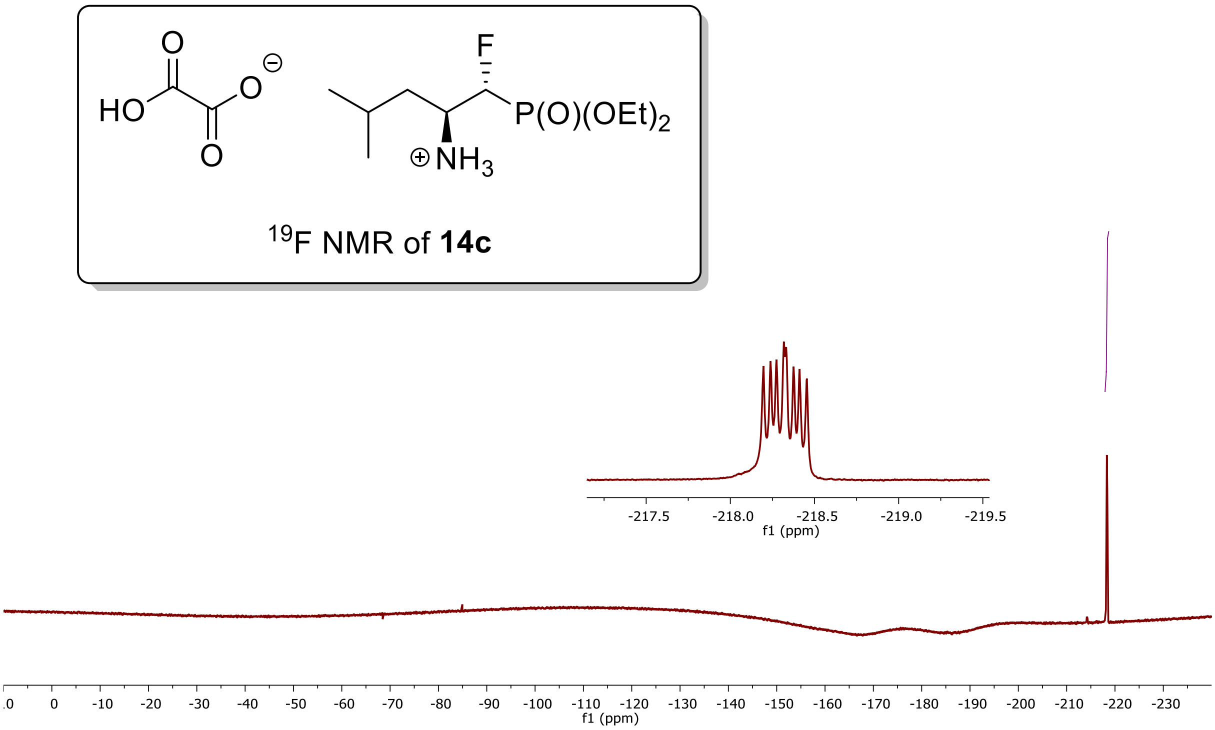
HO
O
O
O
F
P(O)(OEt)2
NH3
19F NMR of 14c
-217.5 -218.0 -218.5 -219.0 -219.5
f1 (ppm)
0 -10 -20 -30 -40 -50 -60 -70 -80 -90 -100 -110 -120 -130 -140 -150 -160 -170 -180 -190 -200 -210 -220 -230
f1 (ppm)

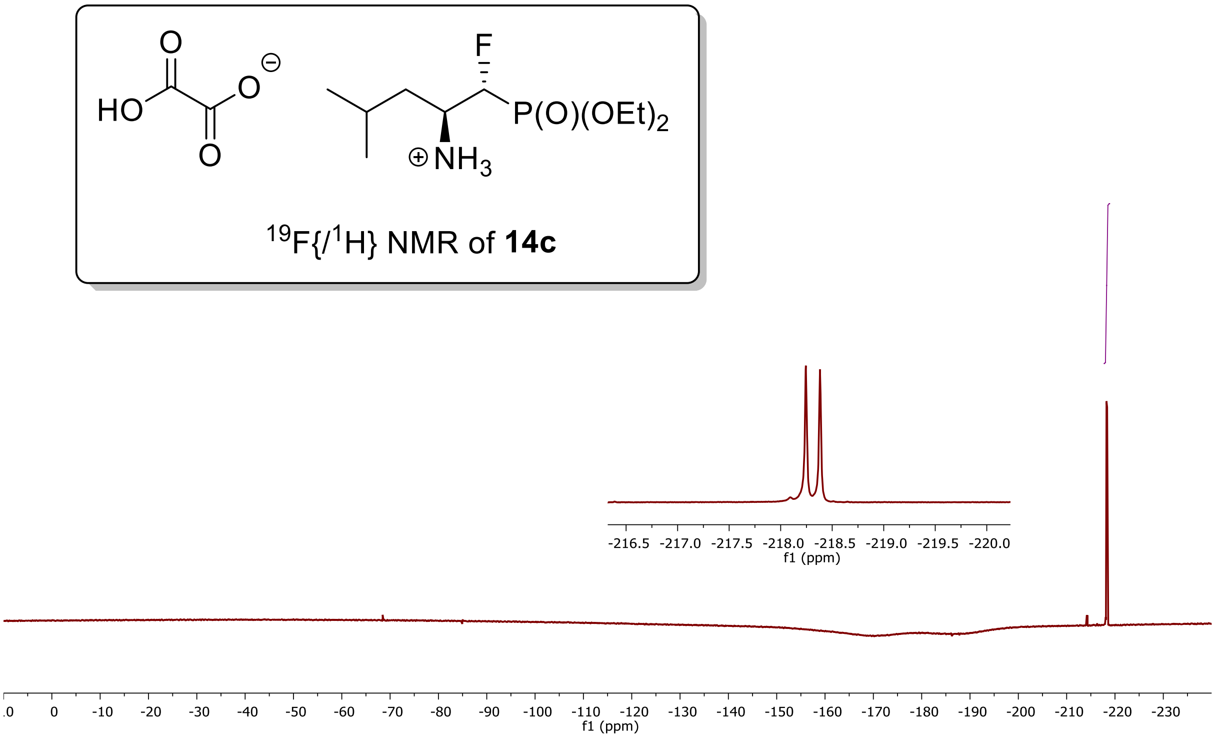
HO
O
O
O
F
P(O)(OEt)2
NH3
19F{/1H} NMR of 14c
-216.5 -217.0 -217.5 -218.0 -218.5 -219.0 -219.5 -220.0
f1 (ppm)
0 -10 -20 -30 -40 -50 -60 -70 -80 -90 -100 -110 -120 -130 -140 -150 -160 -170 -180 -190 -200 -210 -220 -230
f1 (ppm)

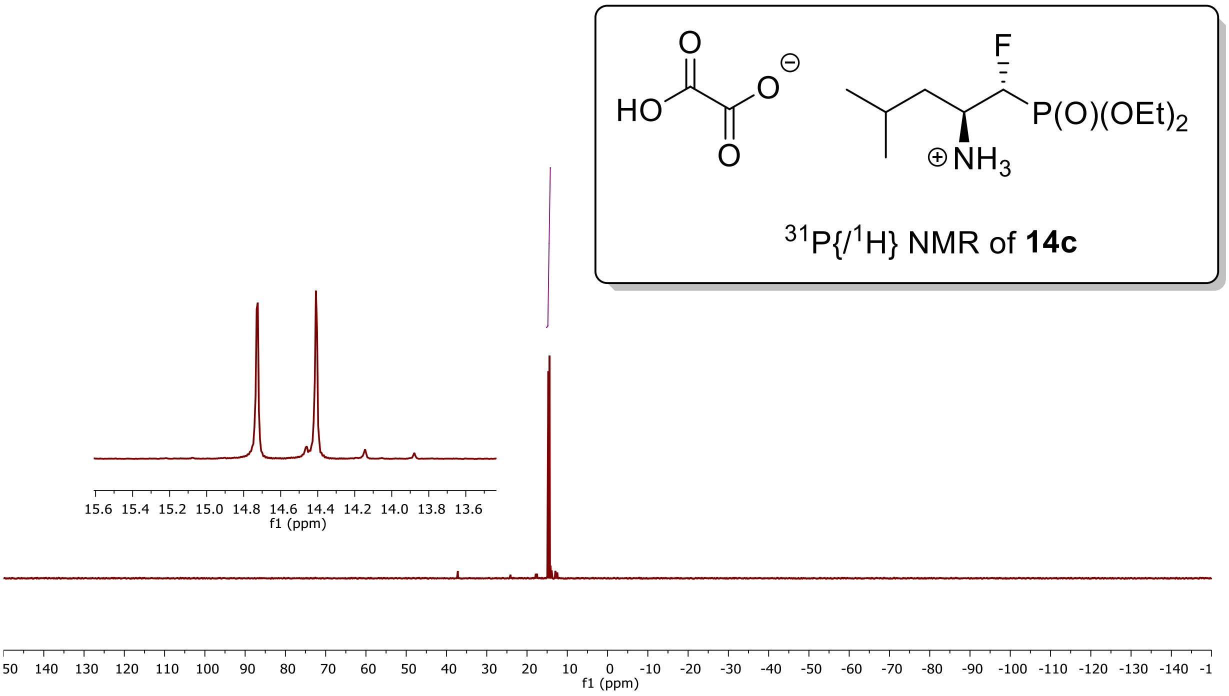
HO
O
O
O
F
P(O)(OEt)2
NH3
31P{/1H} NMR of 14c
15.6 15.4 15.2 15.0 14.8 14.6 14.4 14.2 14.0 13.8 13.6
f1 (ppm)
140 130 120 110 100 90 80 70 60 50 40 30 20 10 0 -10 -20 -30 -40 -50 -60 -70 -80 -90 -100 -110 -120 -130 -140
f1 (ppm)

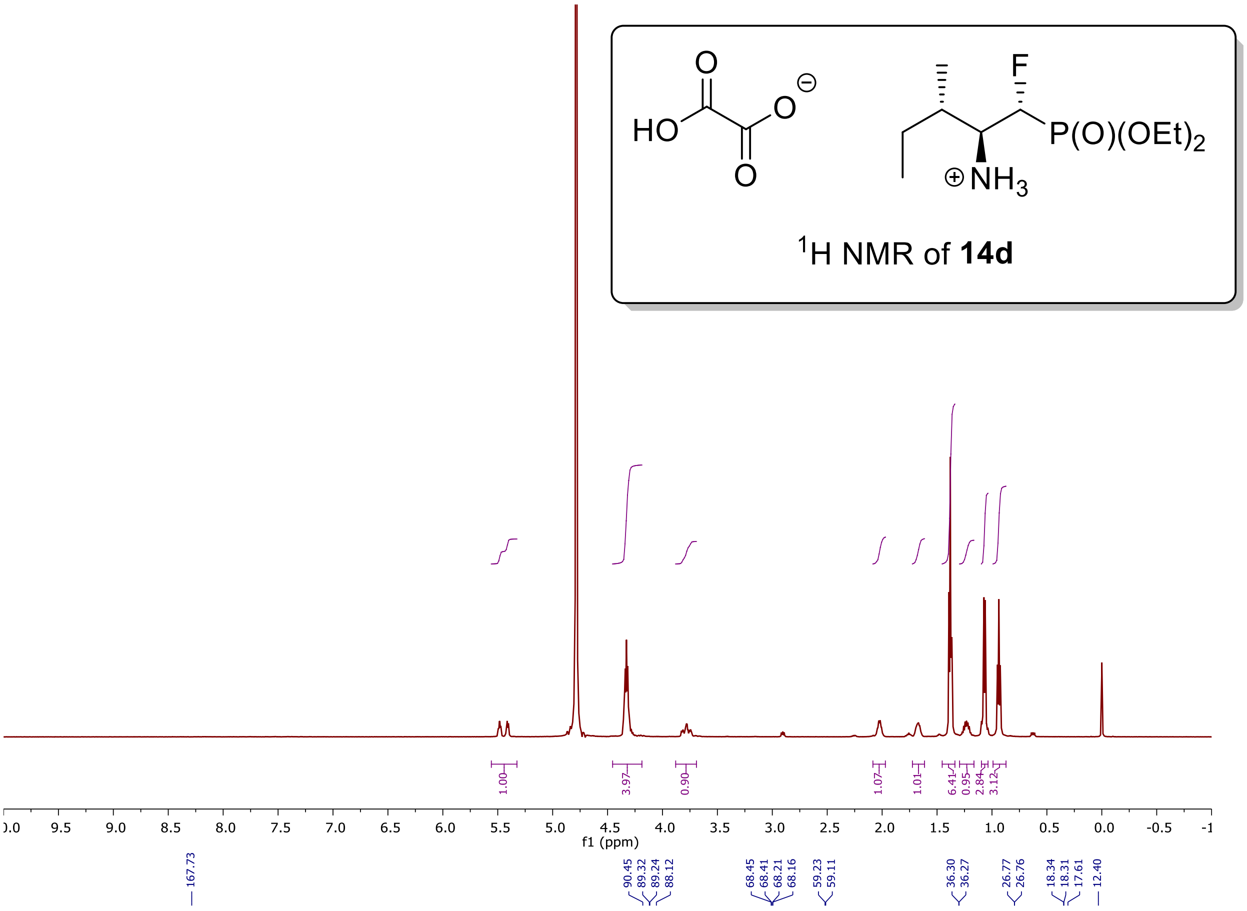
HO
O
O
O
F
P(O)(OEt)$_2$
NH$_3$
^{1}H NMR of **14d**
1.00
3.97
0.90
1.07
1.01
6.41
0.95
2.84
3.12
f1 (ppm)
167.73
90.45
89.32
89.24
88.12
68.45
68.41
68.21
68.16
59.23
59.11
36.30
36.27
26.77
26.76
18.34
18.31
17.61
12.40

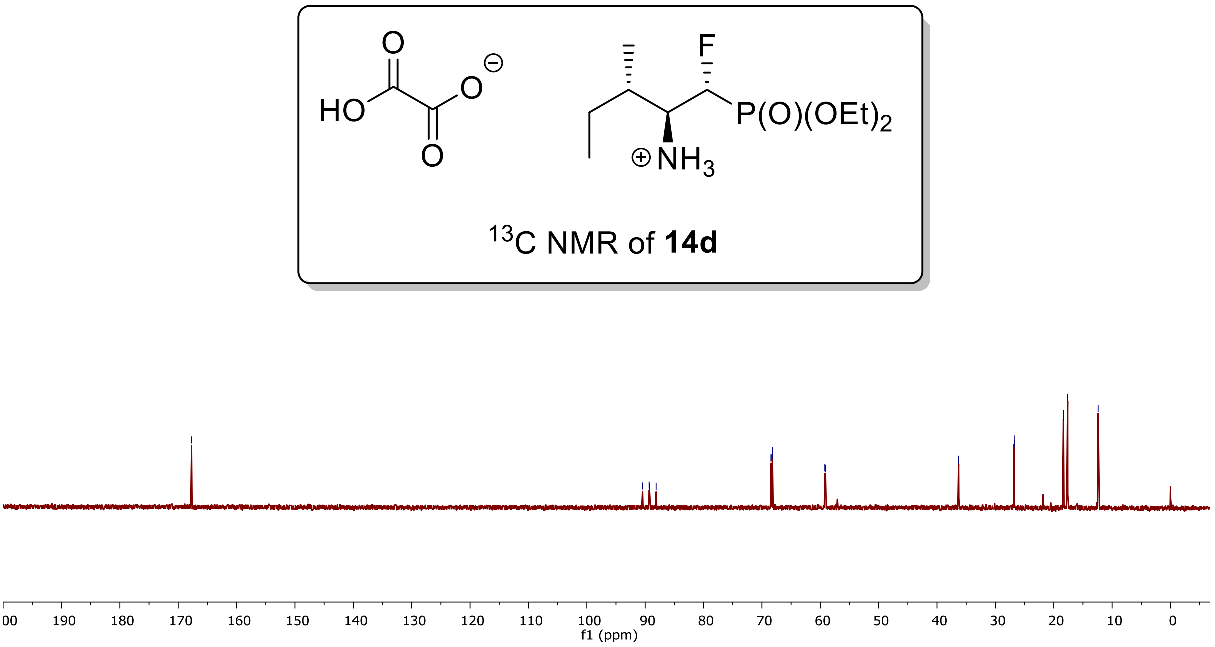
HO
O
O
O
F
P(O)(OEt)$_2$
NH$_3$
^{13}C NMR of **14d**
f1 (ppm)

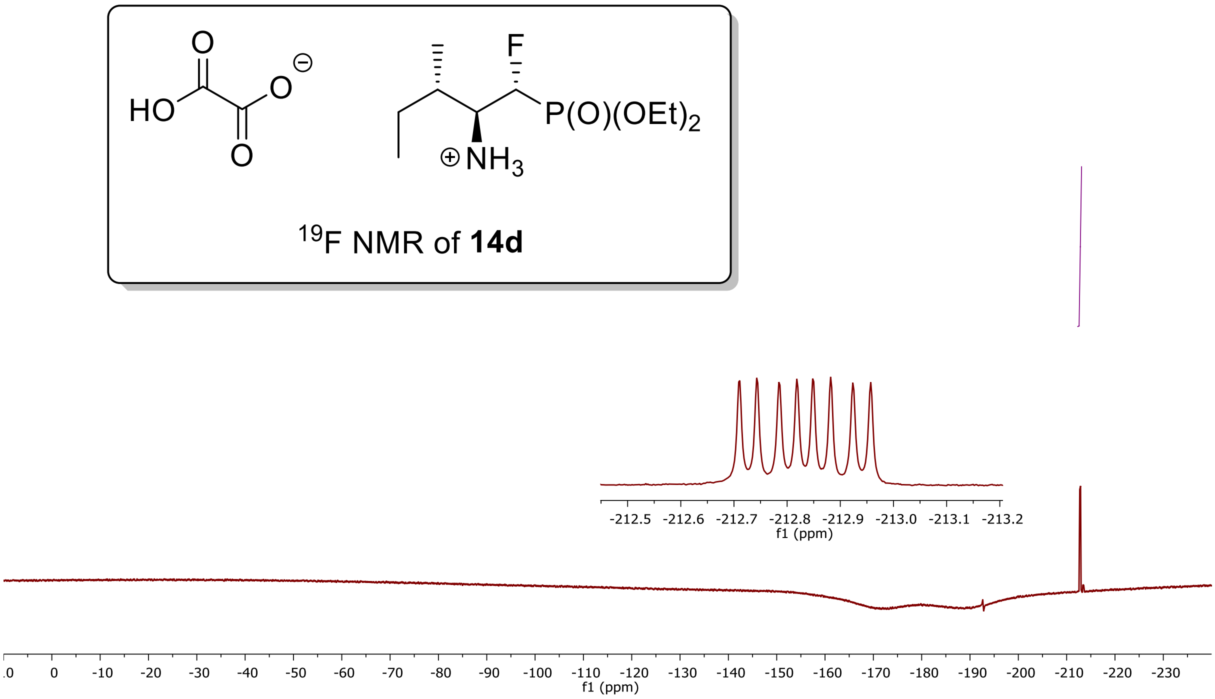

HO
O
O
O
F
P(O)(OEt)2
NH3
19F NMR of 14d
-212.5 -212.6 -212.7 -212.8 -212.9 -213.0 -213.1 -213.2
f1 (ppm)
0 -10 -20 -30 -40 -50 -60 -70 -80 -90 -100 -110 -120 -130 -140 -150 -160 -170 -180 -190 -200 -210 -220 -230
f1 (ppm)

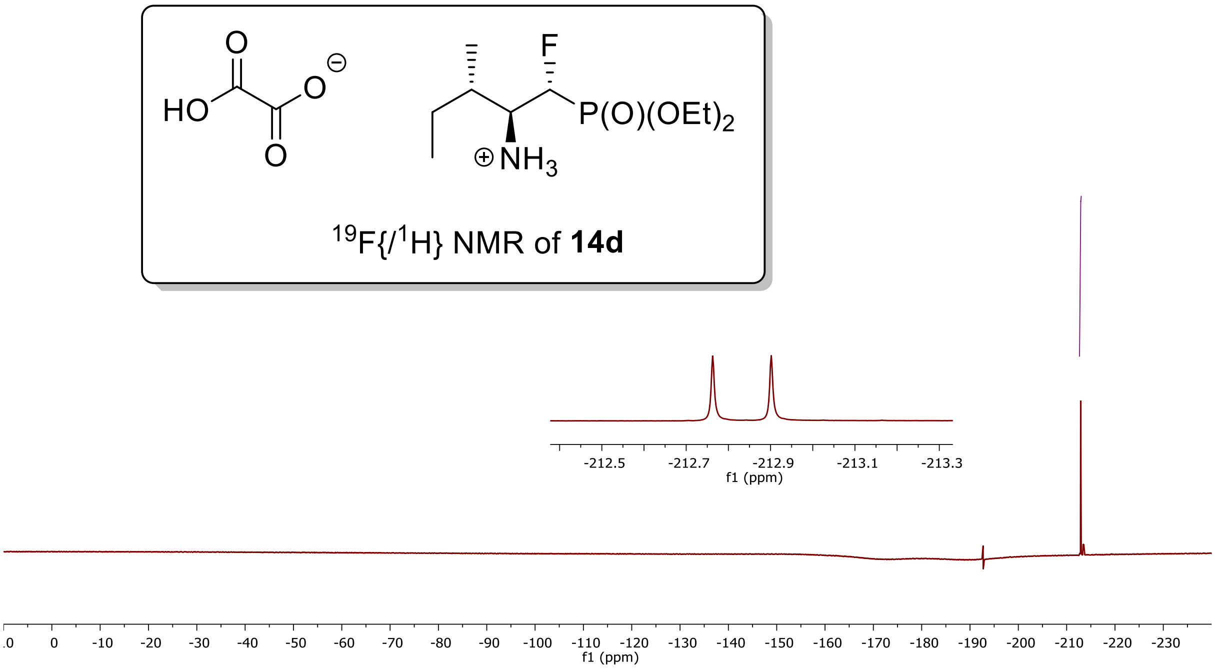

HO
O
O
O
F
P(O)(OEt)2
NH3
19F{/1H} NMR of 14d
-212.5 -212.7 -212.9 -213.1 -213.3
f1 (ppm)
0 -10 -20 -30 -40 -50 -60 -70 -80 -90 -100 -110 -120 -130 -140 -150 -160 -170 -180 -190 -200 -210 -220 -230
f1 (ppm)

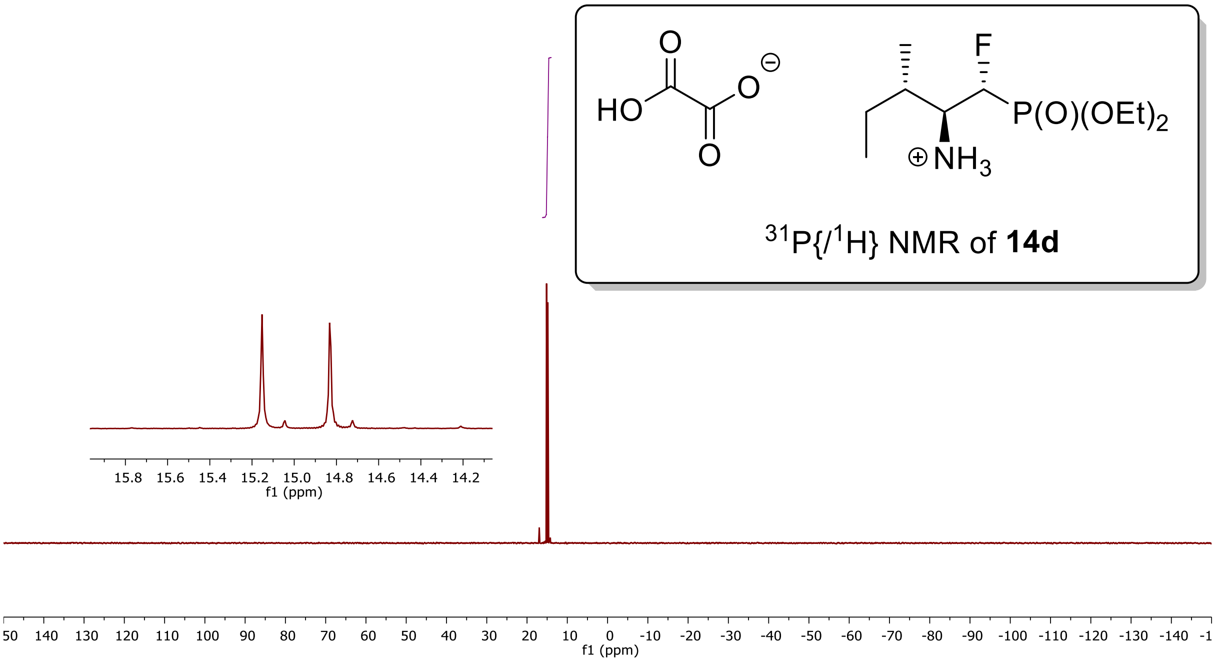
HO
O
O
O
F
P(O)(OEt)2
NH3
31P{/1H} NMR of 14d
f1 (ppm)

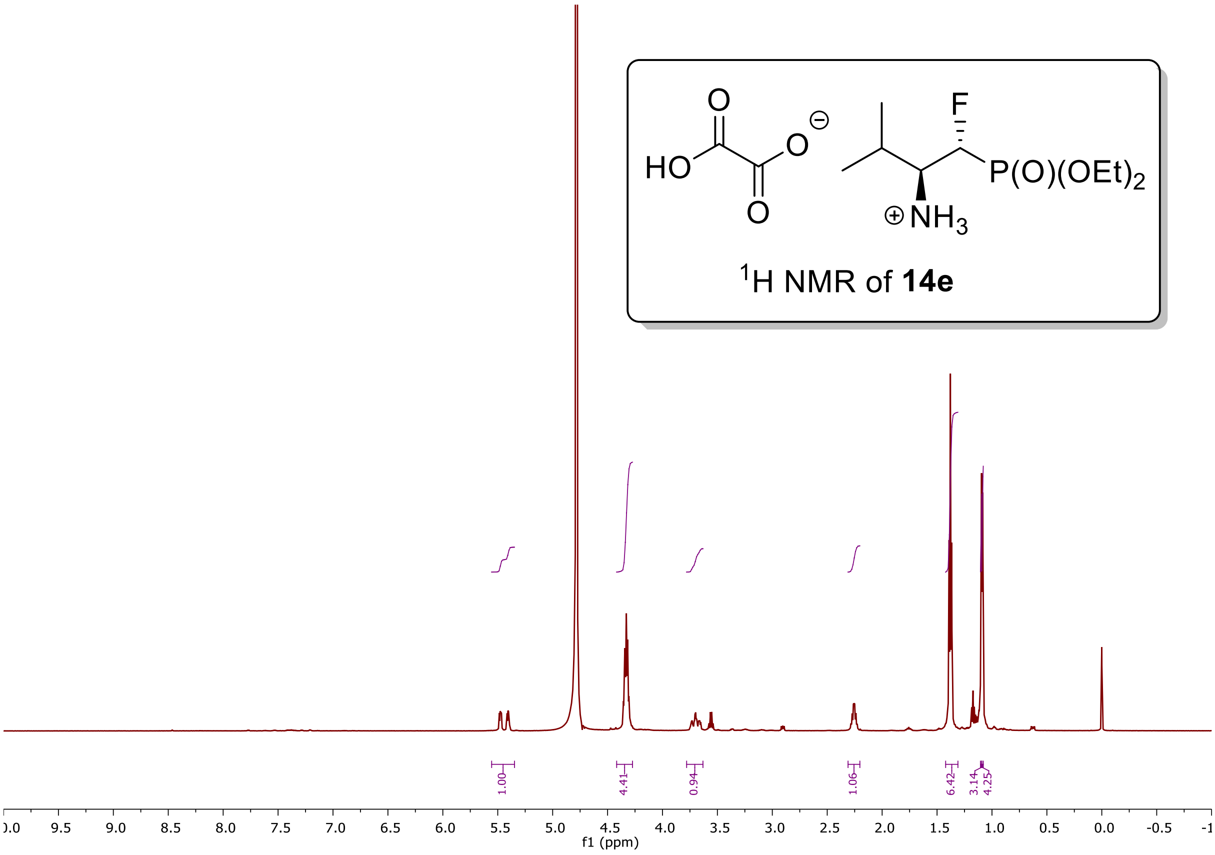
HO
O
O
O
F
P(O)(OEt)2
NH3
1H NMR of 14e
f1 (ppm)

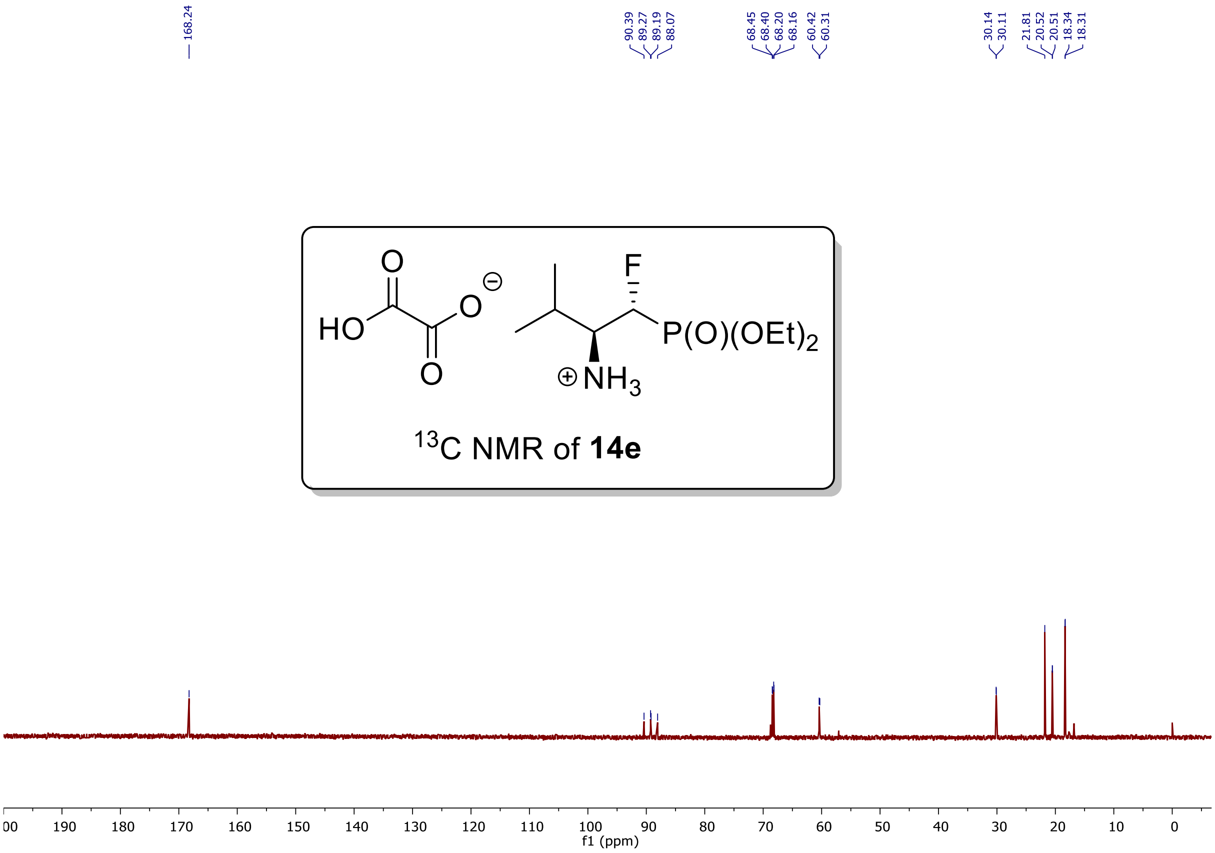
168.24
90.39
89.27
89.19
88.07
68.45
68.40
68.20
68.16
60.42
60.31
30.14
30.11
21.81
20.52
20.51
18.34
18.31
O
HO
O
O
F
P(O)(OEt)2
NH3
13C NMR of 14e
f1 (ppm)

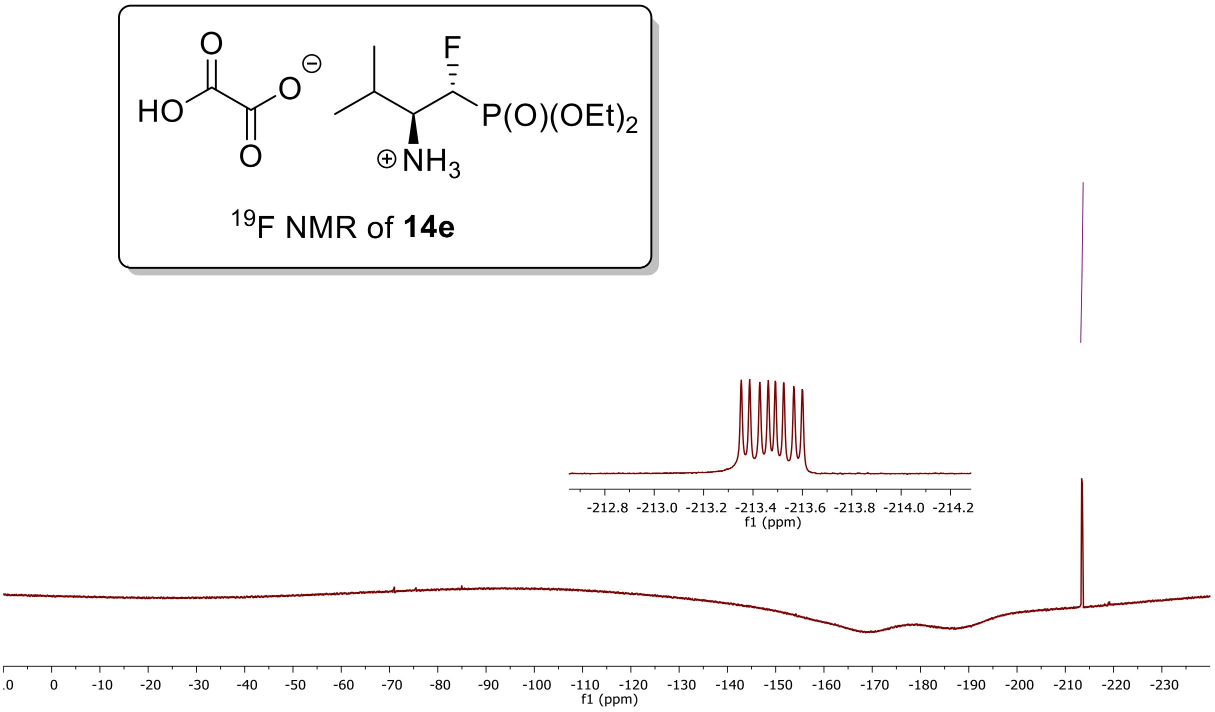
O
HO
O
O
F
P(O)(OEt)2
NH3
19F NMR of 14e
-212.8 -213.0 -213.2 -213.4 -213.6 -213.8 -214.0 -214.2
f1 (ppm)
f1 (ppm)

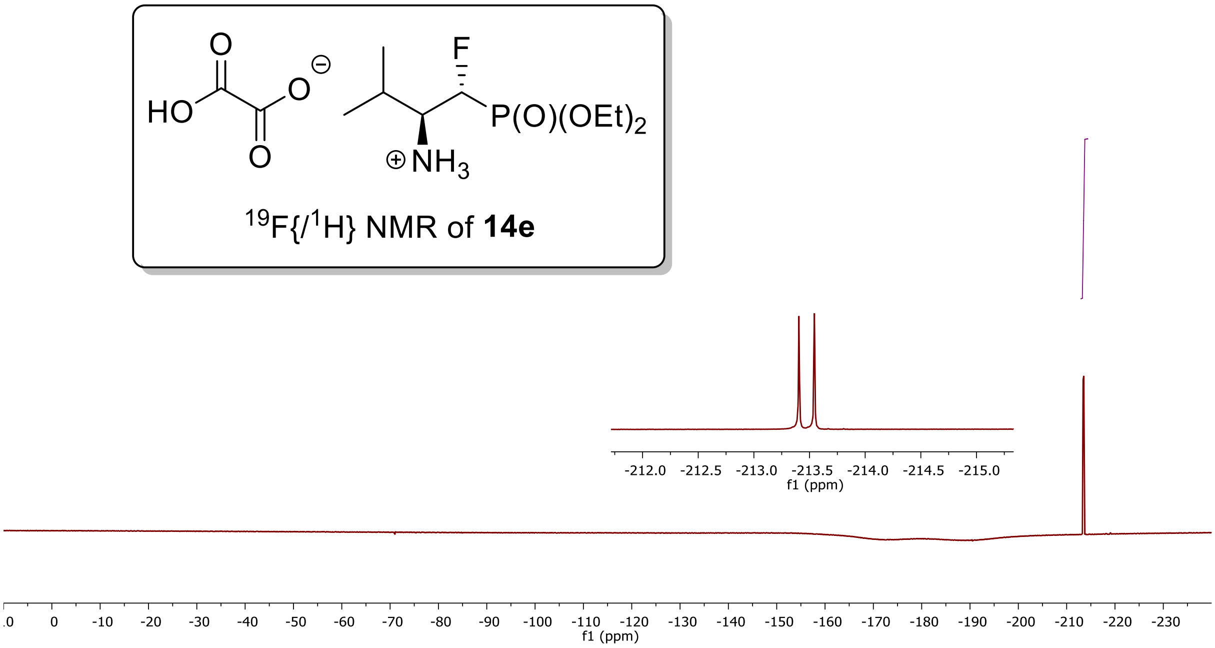
HO
O
O
O
F
P(O)(OEt)2
NH3
19F{/1H} NMR of 14e
-212.0 -212.5 -213.0 -213.5 -214.0 -214.5 -215.0
f1 (ppm)
0 -10 -20 -30 -40 -50 -60 -70 -80 -90 -100 -110 -120 -130 -140 -150 -160 -170 -180 -190 -200 -210 -220 -230
f1 (ppm)

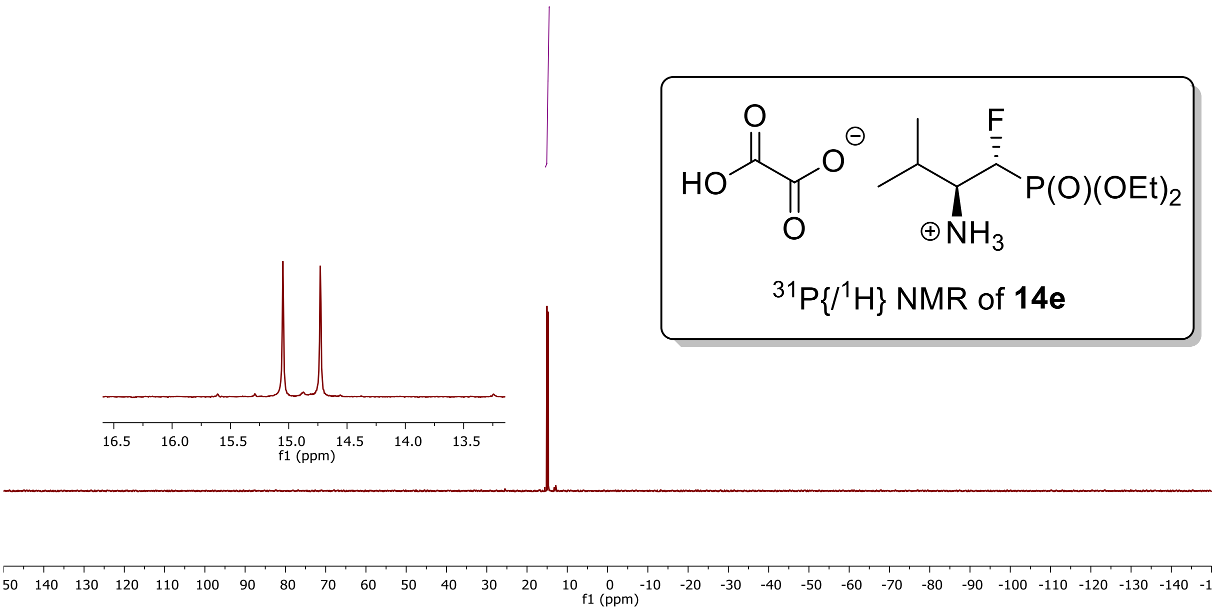
HO
O
O
O
F
P(O)(OEt)2
NH3
31P{/1H} NMR of 14e
16.5 16.0 15.5 15.0 14.5 14.0 13.5
f1 (ppm)
140 130 120 110 100 90 80 70 60 50 40 30 20 10 0 -10 -20 -30 -40 -50 -60 -70 -80 -90 -100 -110 -120 -130 -140
f1 (ppm)

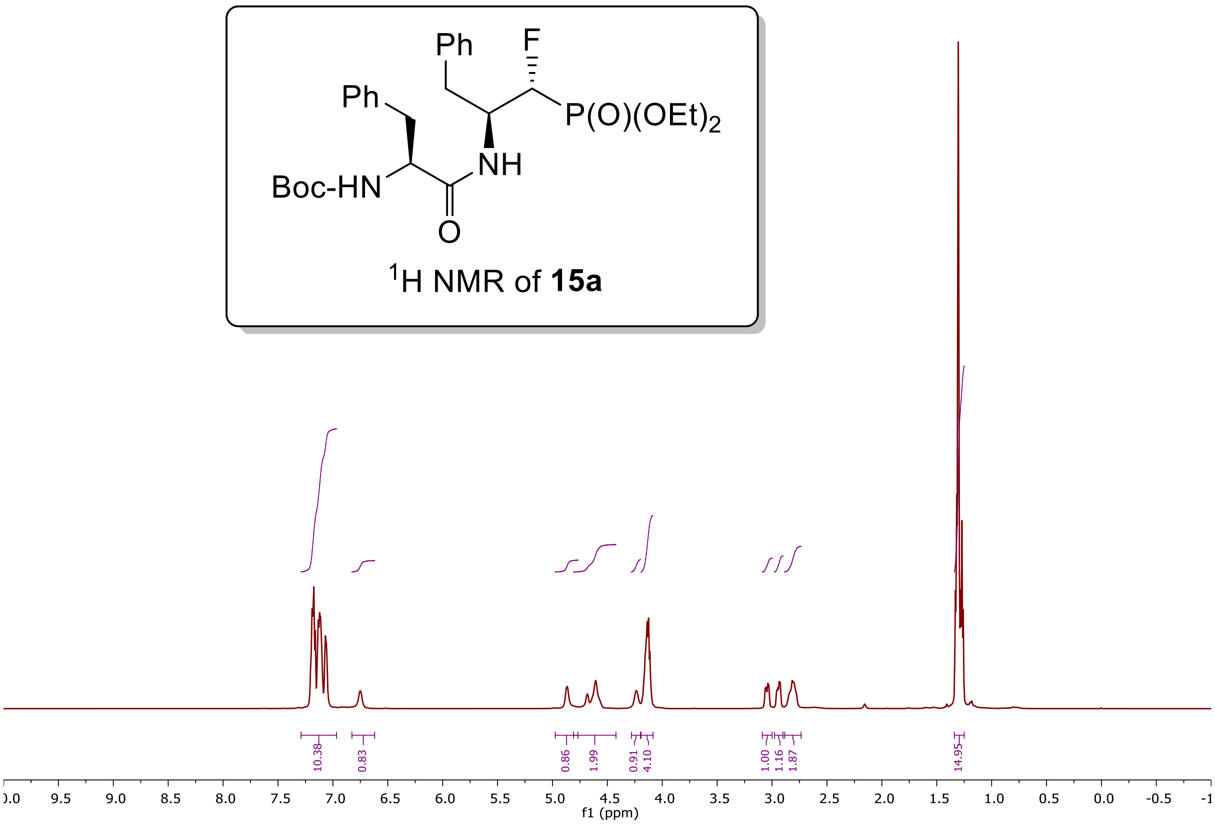
Ph
F
Ph
P(O)(OEt)2
NH
Boc-HN
O
1H NMR of 15a
10.38
0.83
0.86
1.99
0.91
4.10
1.00
1.16
1.87
14.95
f1 (ppm)

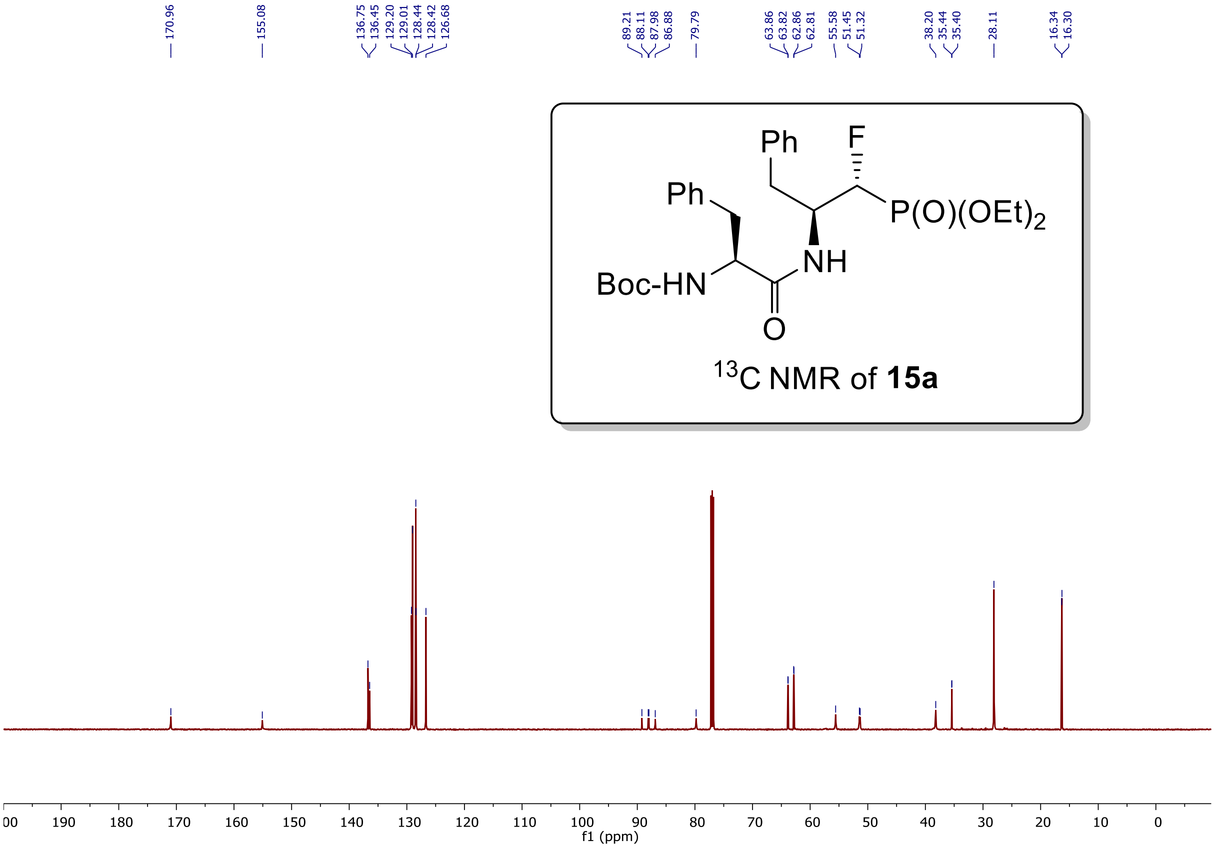
170.96
155.08
136.75
136.45
129.20
129.01
128.44
128.42
126.68
89.21
88.11
87.98
86.88
79.79
63.86
63.82
62.86
62.81
55.58
51.45
51.32
38.20
35.44
35.40
28.11
16.34
16.30
Ph
F
Ph
P(O)(OEt)2
NH
Boc-HN
O
13C NMR of 15a
f1 (ppm)

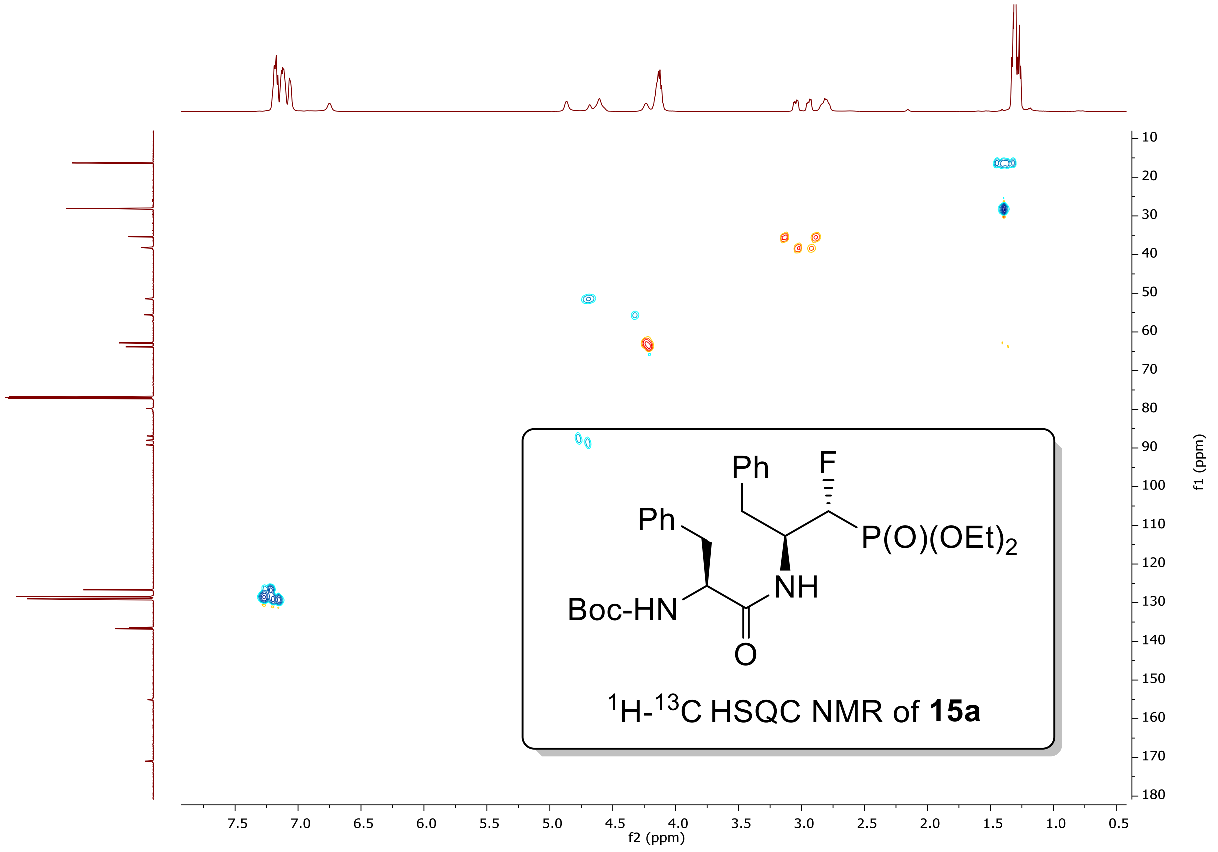
Ph
F
P(O)(OEt)2
Ph
NH
Boc-HN
O
1H-13C HSQC NMR of 15a
f2 (ppm)
f1 (ppm)

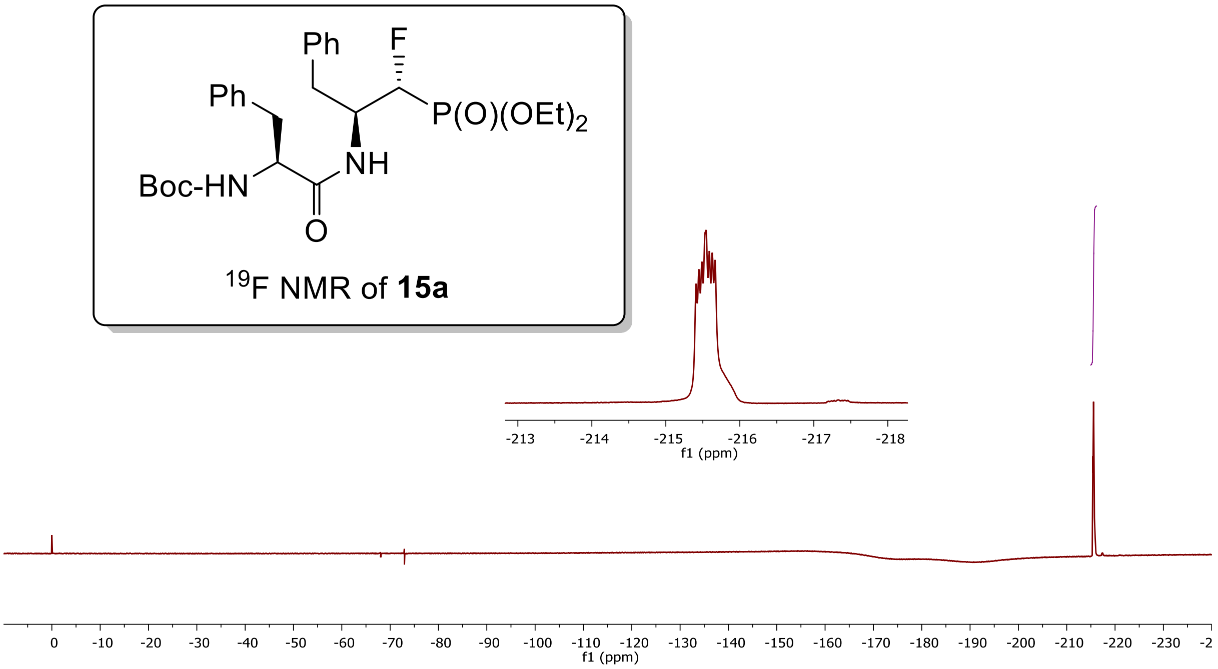
Ph
F
P(O)(OEt)2
Ph
NH
Boc-HN
O
19F NMR of 15a
f1 (ppm)
f1 (ppm)

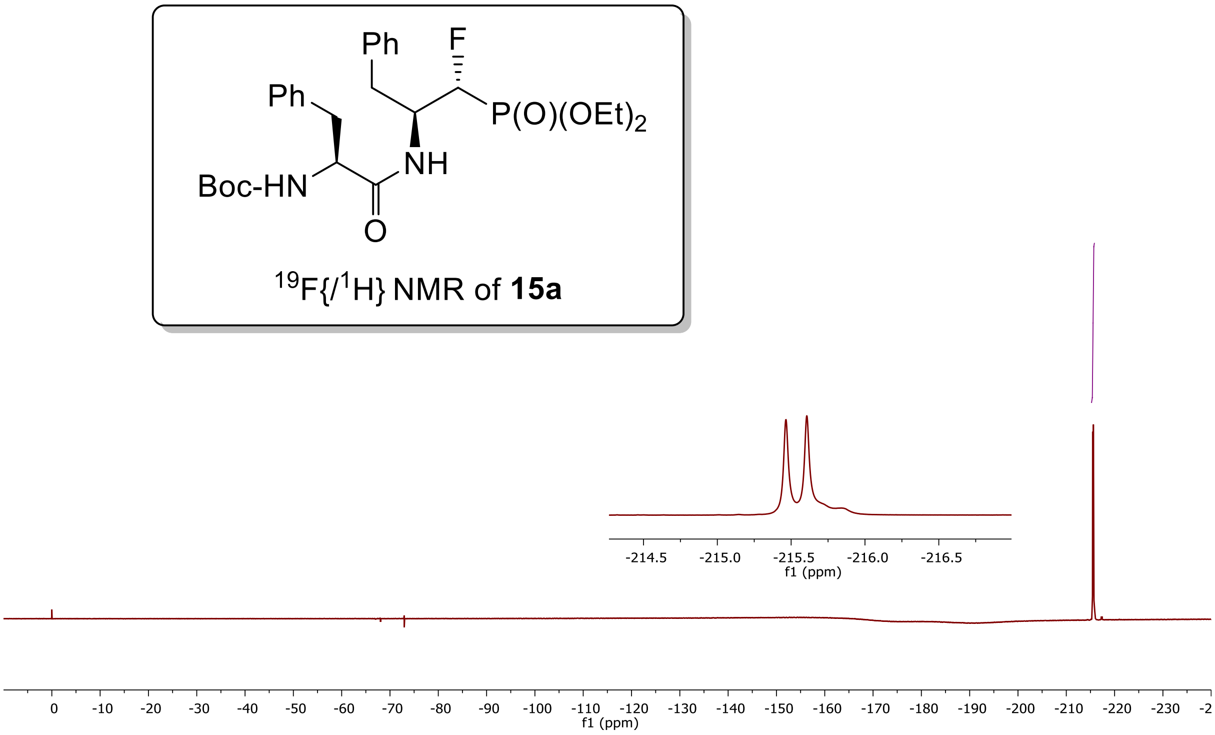
Ph
F
Ph
P(O)(OEt)$_2$
NH
Boc-HN
O
^{19}F{/^{1}H} NMR of **15a**
f1 (ppm)
f1 (ppm)

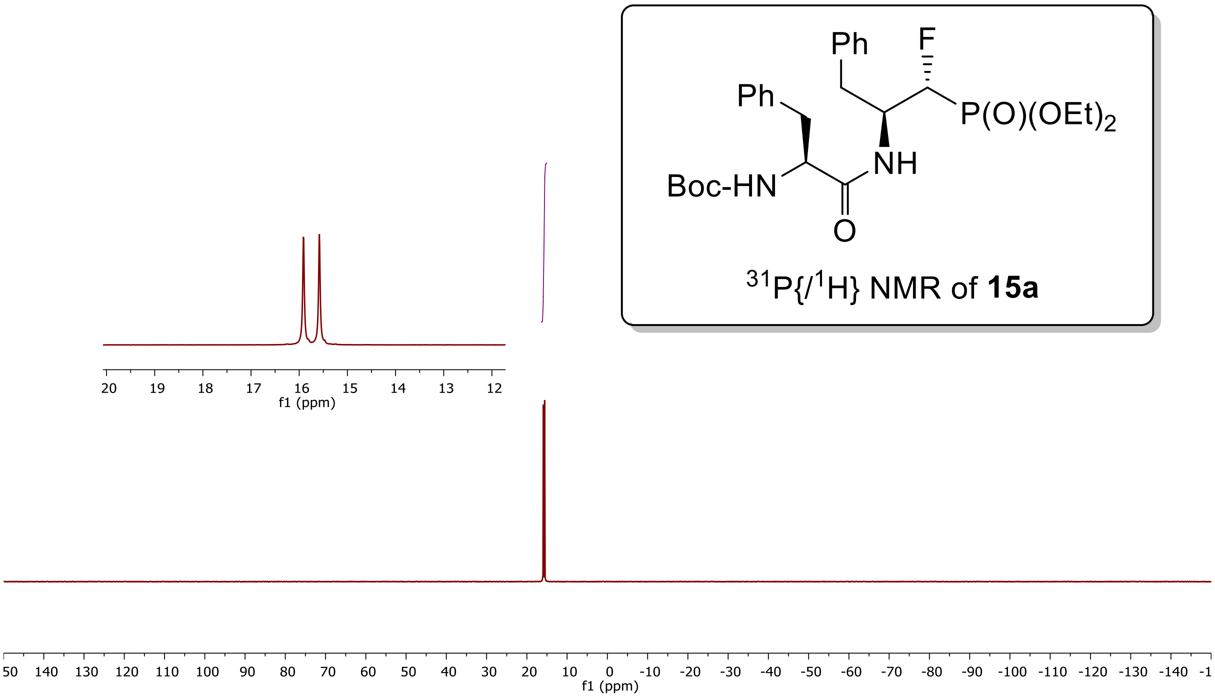
Ph
F
Ph
P(O)(OEt)$_2$
NH
Boc-HN
O
^{31}P{/^{1}H} NMR of **15a**
f1 (ppm)
f1 (ppm)

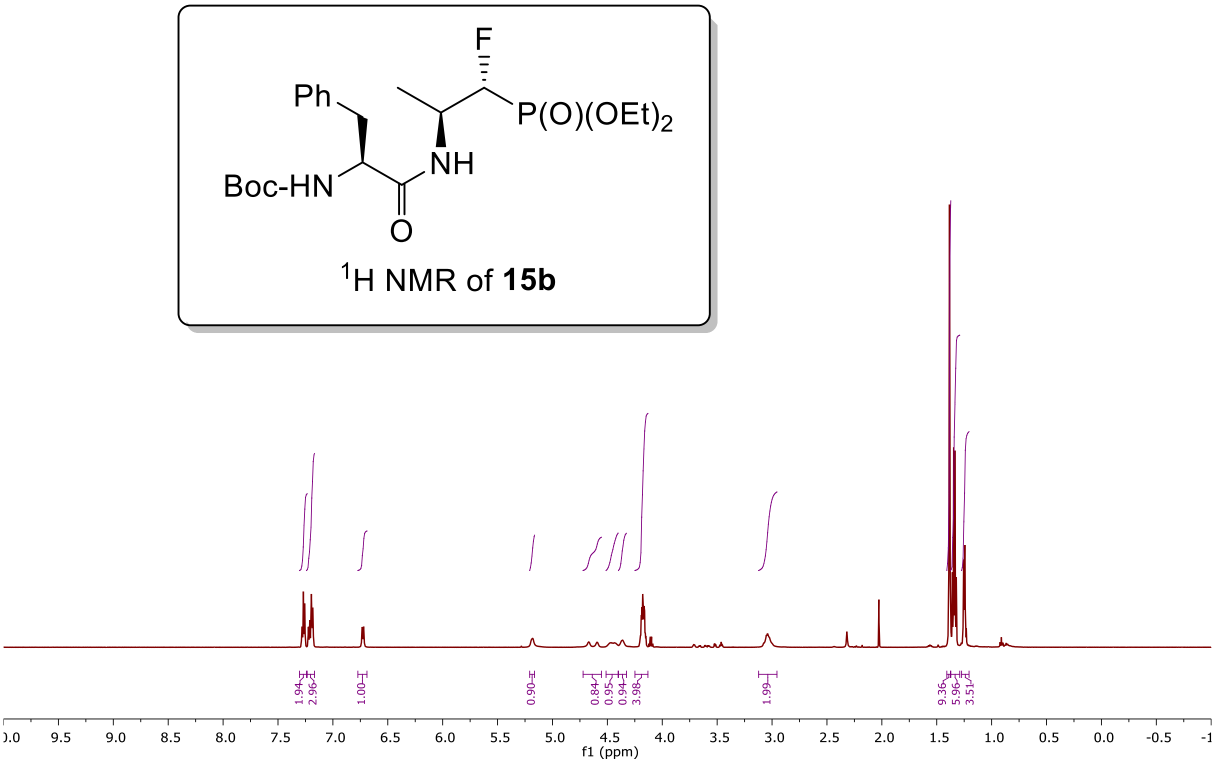
F
Ph
P(O)(OEt)$_2$
NH
Boc-HN
O
^{1}H NMR of **15b**
1.94
2.96
1.00
0.90
0.84
0.95
0.94
3.98
1.99
9.36
5.96
3.51
f1 (ppm)

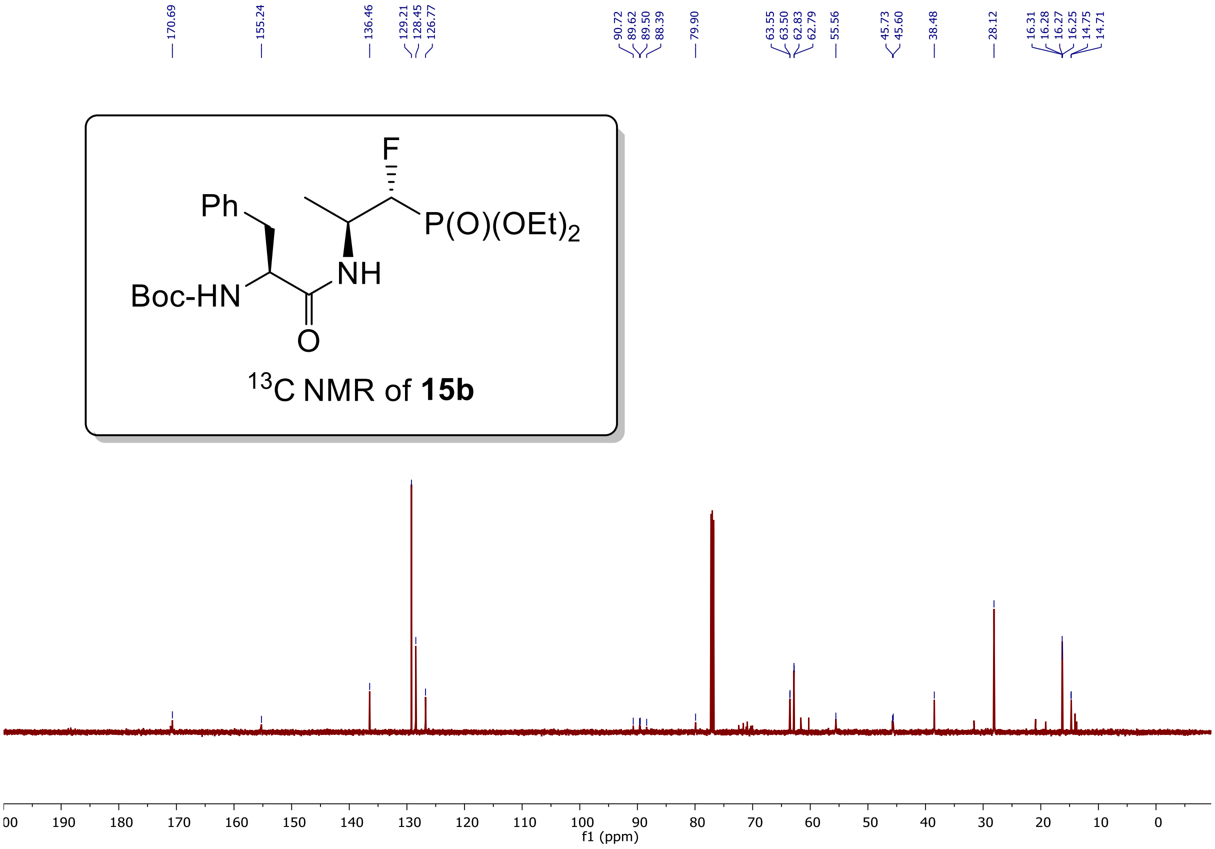
170.69
155.24
136.46
129.21
128.45
126.77
90.72
89.62
89.50
88.39
79.90
63.55
63.50
62.83
62.79
55.56
45.73
45.60
38.48
28.12
16.31
16.28
16.27
16.25
14.75
14.71
F
Ph
P(O)(OEt)$_2$
NH
Boc-HN
O
^{13}C NMR of **15b**
f1 (ppm)

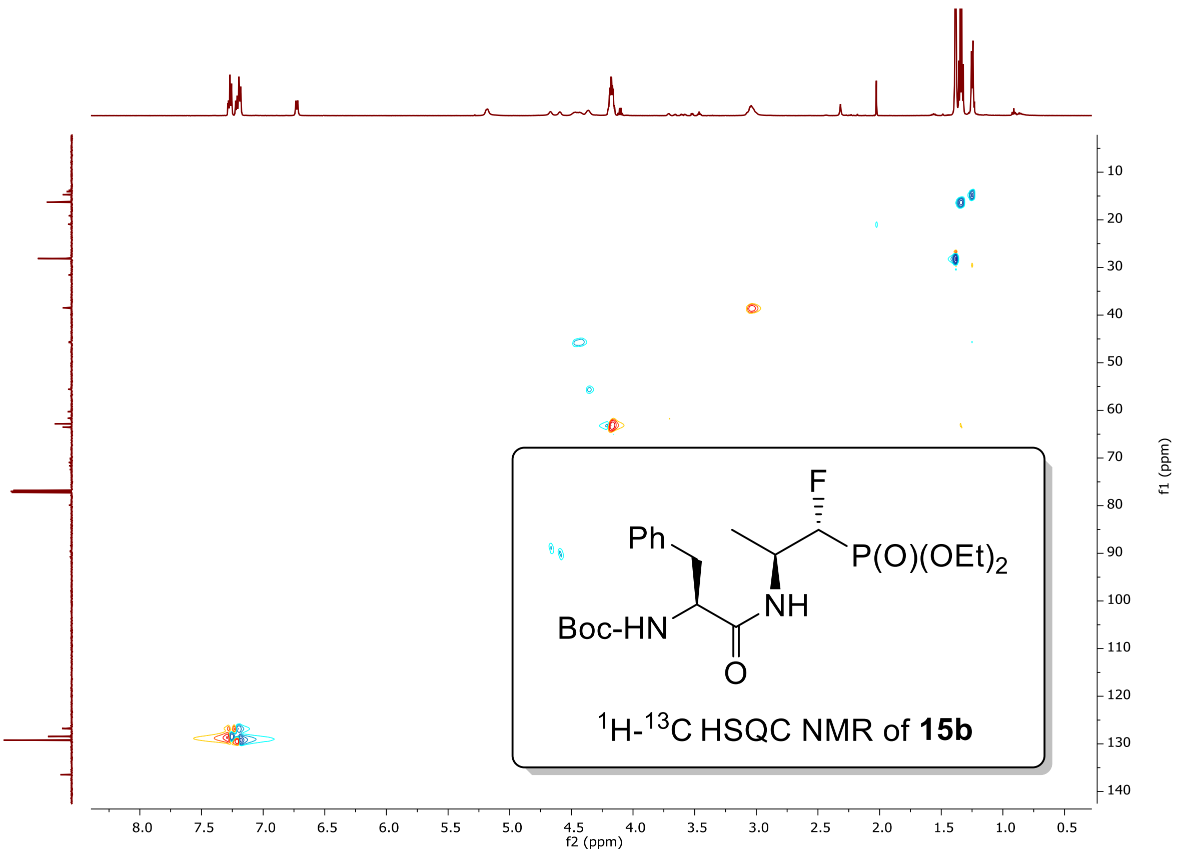

F
Ph
P(O)(OEt)2
NH
Boc-HN
O
1H-13C HSQC NMR of 15b
f2 (ppm)
f1 (ppm)

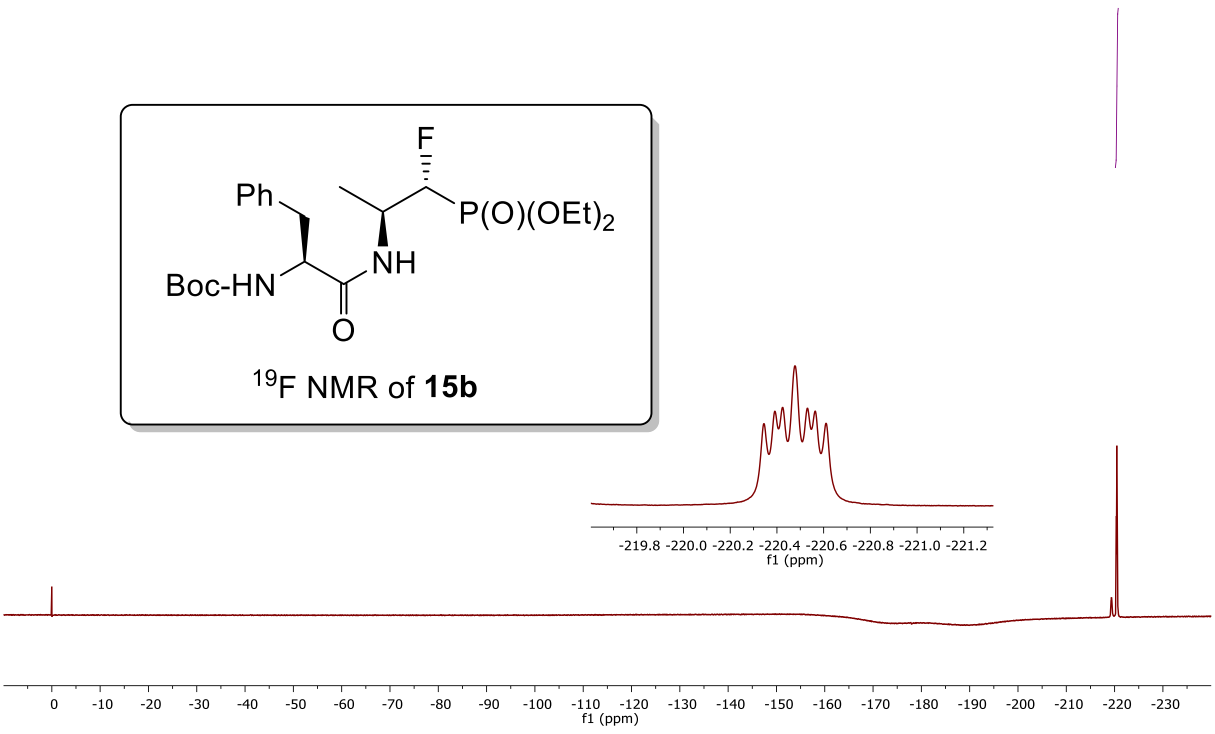

F
Ph
P(O)(OEt)2
NH
Boc-HN
O
19F NMR of 15b
f1 (ppm)

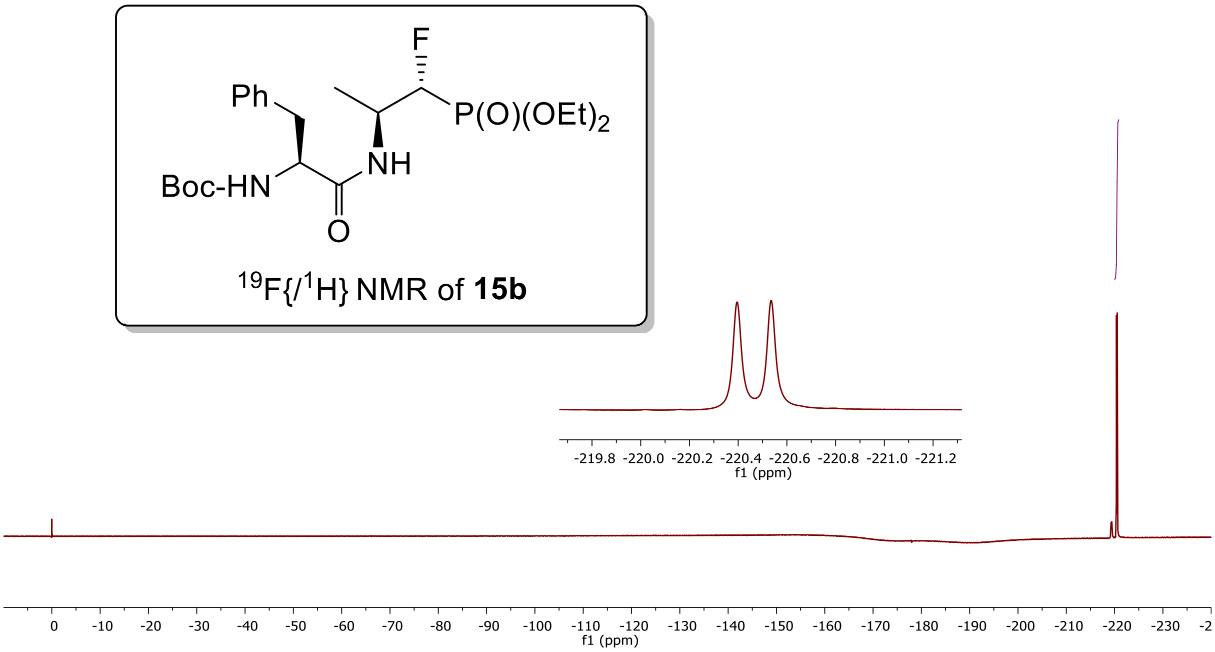

F
Ph
P(O)(OEt)2
Boc-HN
NH
O
19F{/1H} NMR of 15b
-219.8 -220.0 -220.2 -220.4 -220.6 -220.8 -221.0 -221.2
f1 (ppm)
0 -10 -20 -30 -40 -50 -60 -70 -80 -90 -100 -110 -120 -130 -140 -150 -160 -170 -180 -190 -200 -210 -220 -230
f1 (ppm)

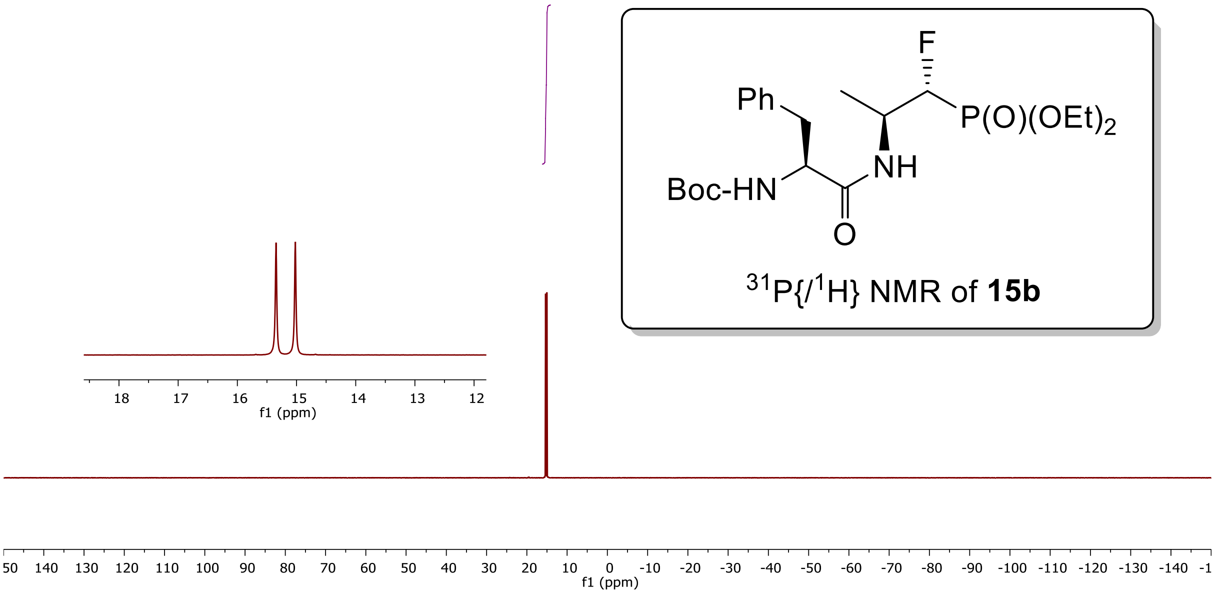

F
Ph
P(O)(OEt)2
Boc-HN
NH
O
31P{/1H} NMR of 15b
18 17 16 15 14 13 12
f1 (ppm)
140 130 120 110 100 90 80 70 60 50 40 30 20 10 0 -10 -20 -30 -40 -50 -60 -70 -80 -90 -100 -110 -120 -130 -140
f1 (ppm)

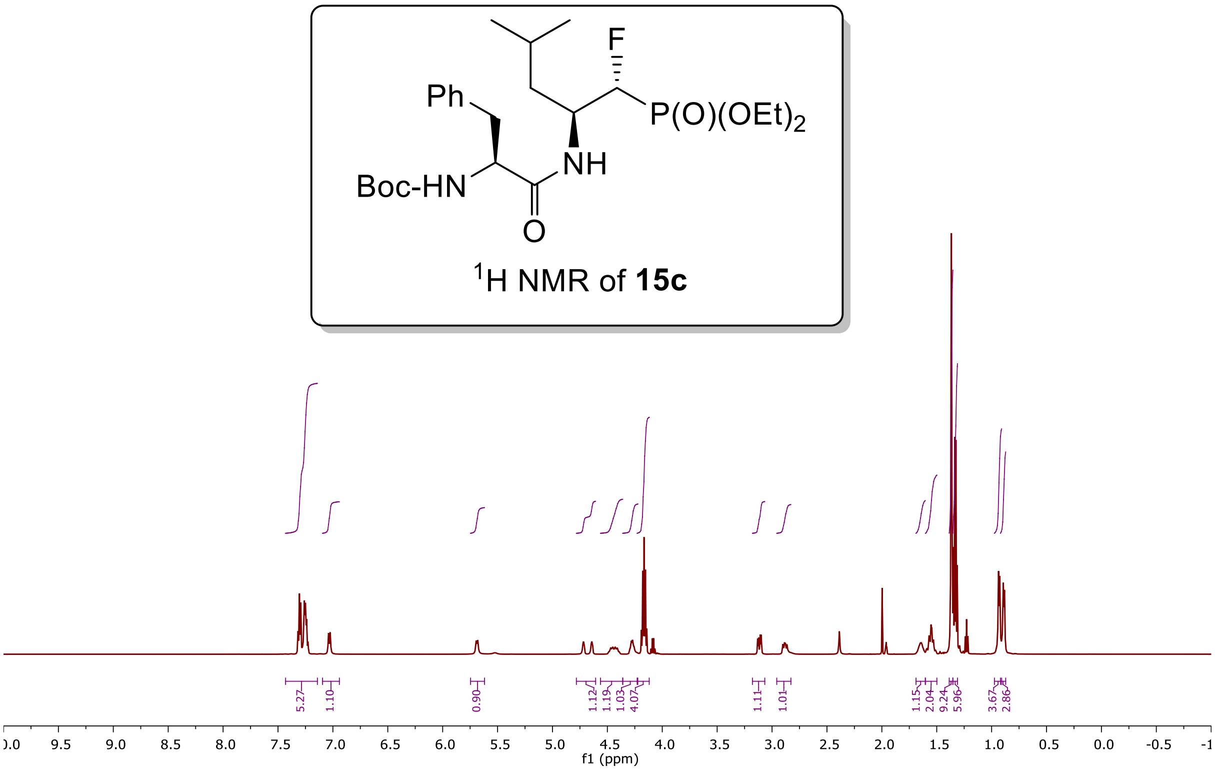

Ph
Boc-HN
NH
O
F
P(O)(OEt)2
1H NMR of 15c
f1 (ppm)

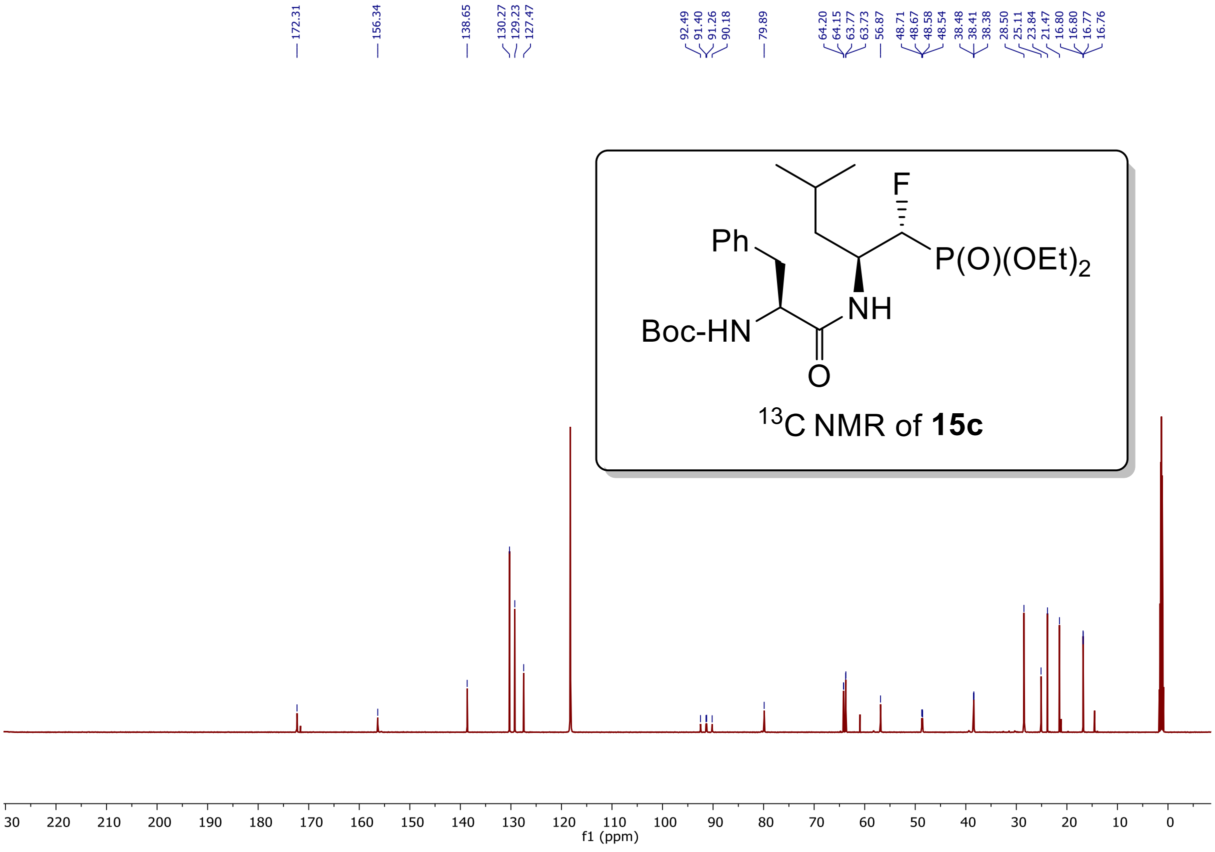

172.31
156.34
138.65
130.27
129.23
127.47
92.49
91.40
91.26
90.18
79.89
64.20
64.15
63.77
63.73
56.87
48.71
48.67
48.58
48.54
38.48
38.41
38.38
28.50
25.11
23.84
21.47
16.80
16.80
16.77
16.76
Ph
Boc-HN
NH
O
F
P(O)(OEt)2
13C NMR of 15c
f1 (ppm)

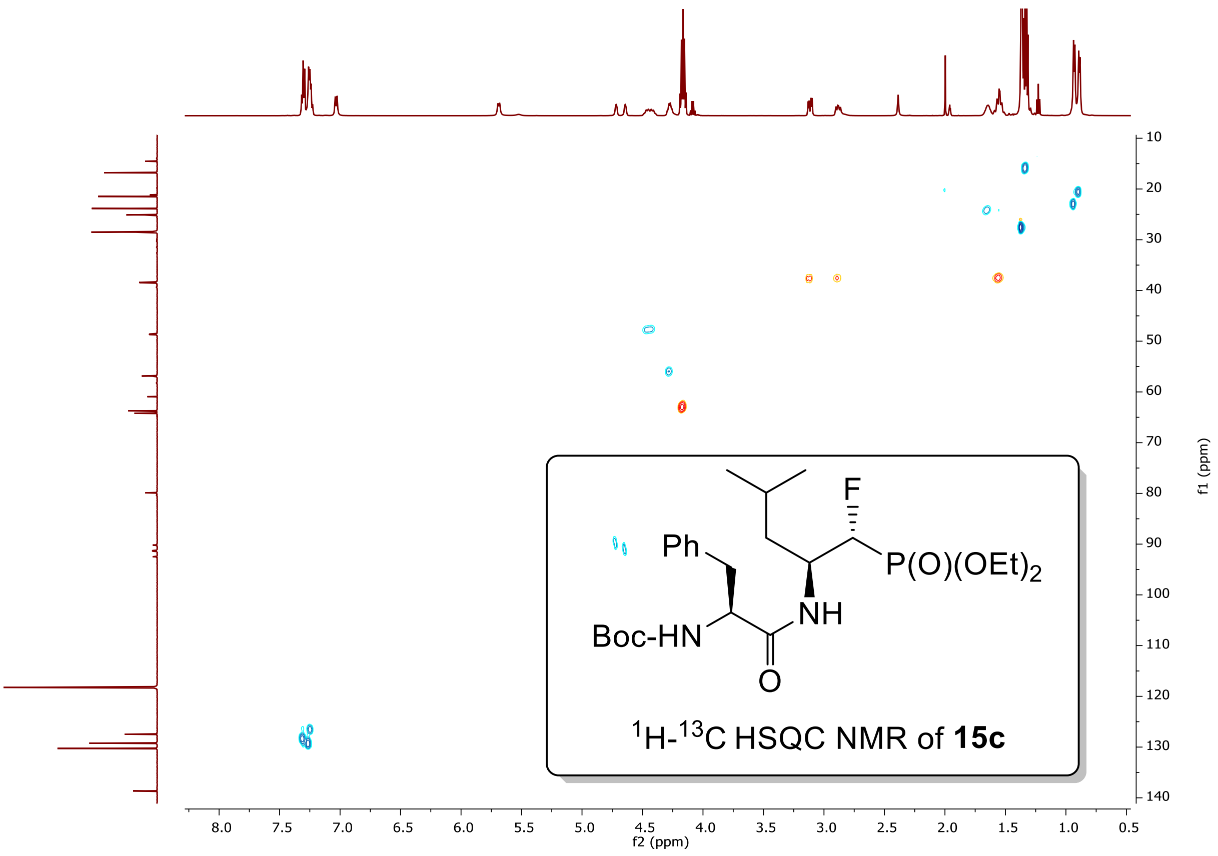
Ph
Boc-HN
NH
O
F
P(O)(OEt)2
1H-13C HSQC NMR of 15c
f2 (ppm)
f1 (ppm)

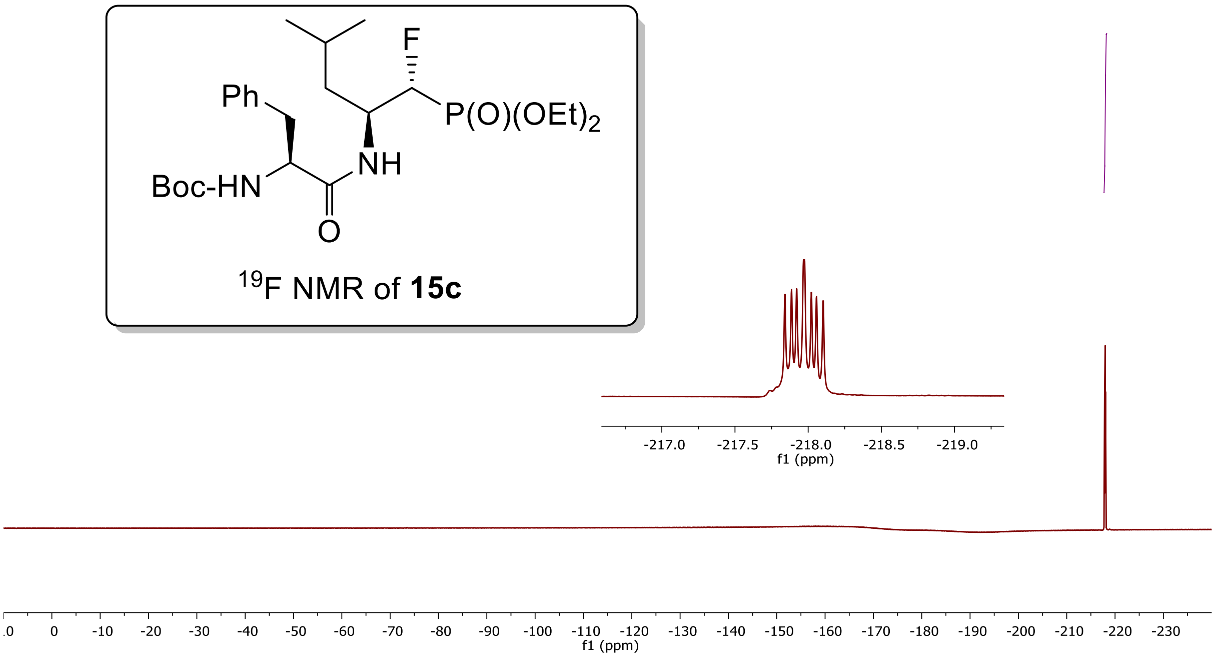
Ph
Boc-HN
NH
O
F
P(O)(OEt)2
19F NMR of 15c
f1 (ppm)

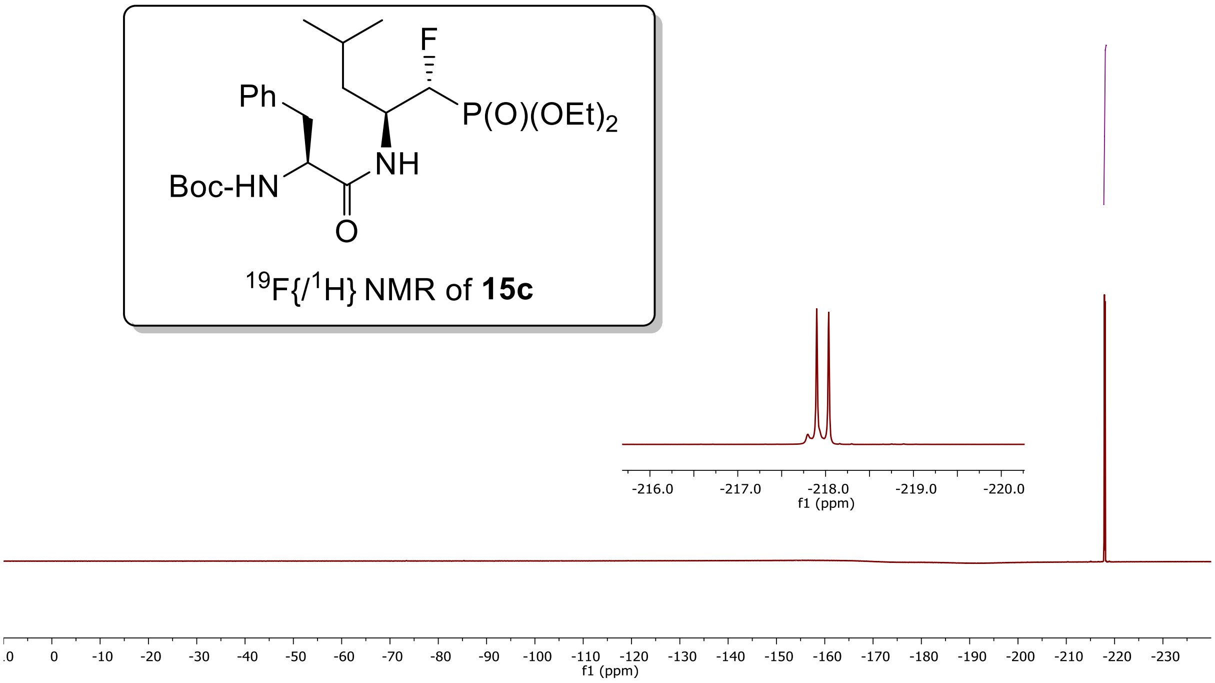
F
Ph
P(O)(OEt)2
NH
Boc-HN
O
19F{/1H} NMR of 15c
f1 (ppm)

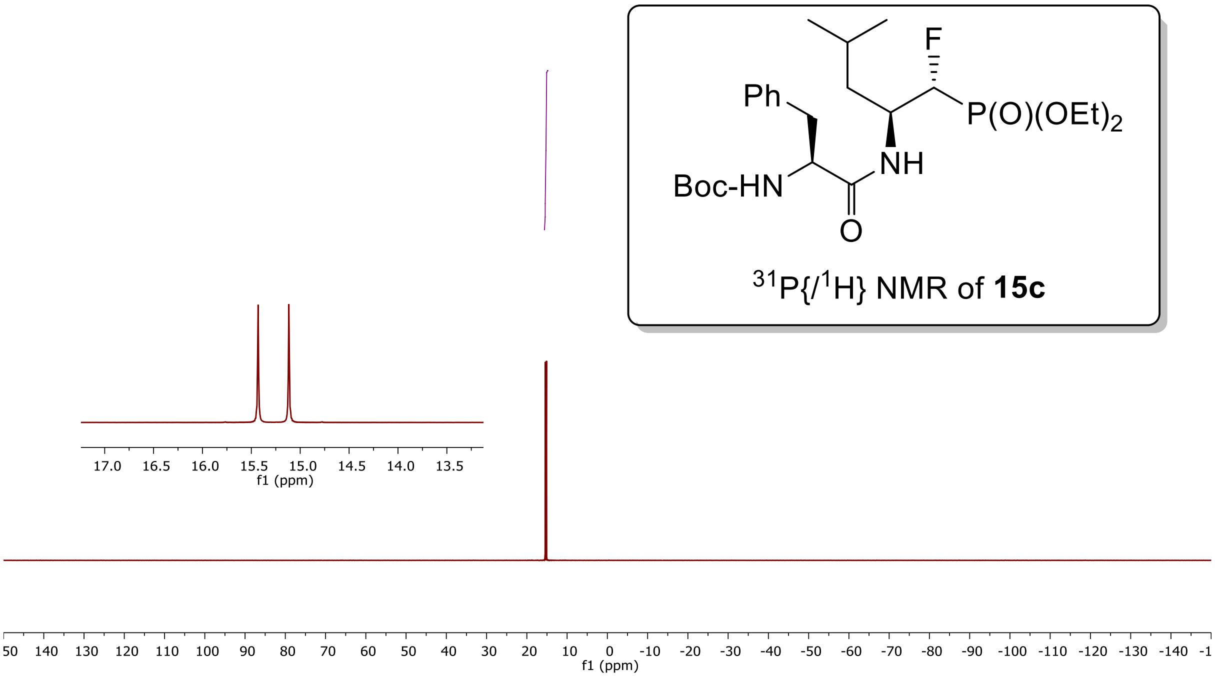
F
Ph
P(O)(OEt)2
NH
Boc-HN
O
31P{/1H} NMR of 15c
f1 (ppm)

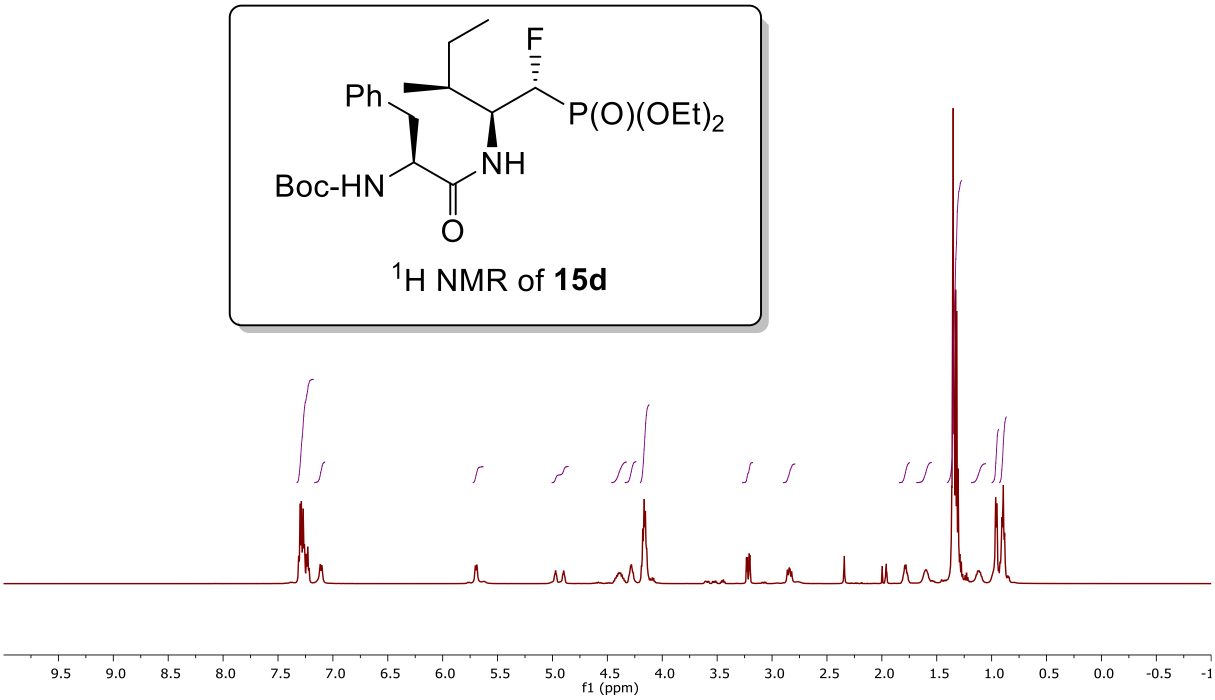
F
Ph
P(O)(OEt)2
NH
Boc-HN
O
1H NMR of 15d
f1 (ppm)

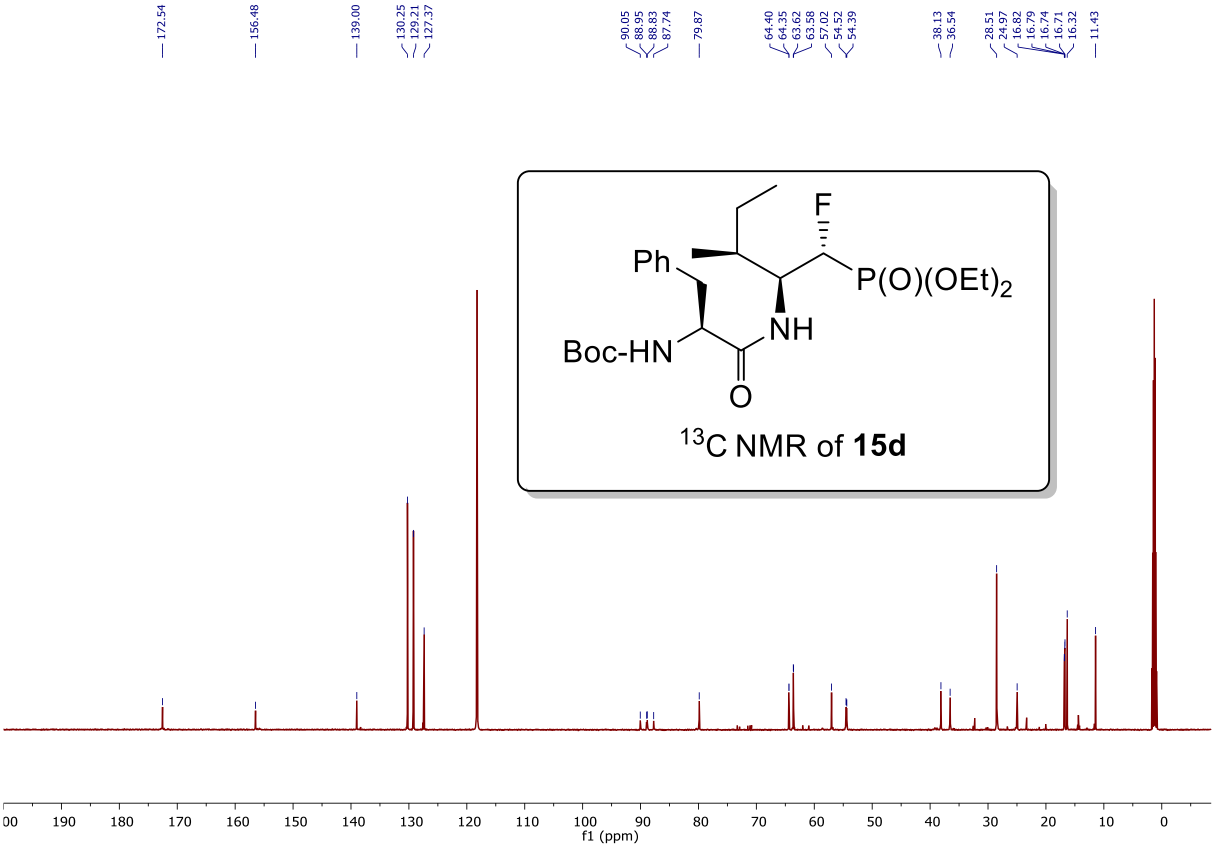
172.54
156.48
139.00
130.25
129.21
127.37
90.05
88.95
88.83
87.74
79.87
64.40
64.35
63.62
63.58
57.02
54.52
54.39
38.13
36.54
28.51
24.97
16.82
16.79
16.74
16.71
16.32
11.43
F
Ph
P(O)(OEt)2
NH
Boc-HN
O
13C NMR of 15d
f1 (ppm)

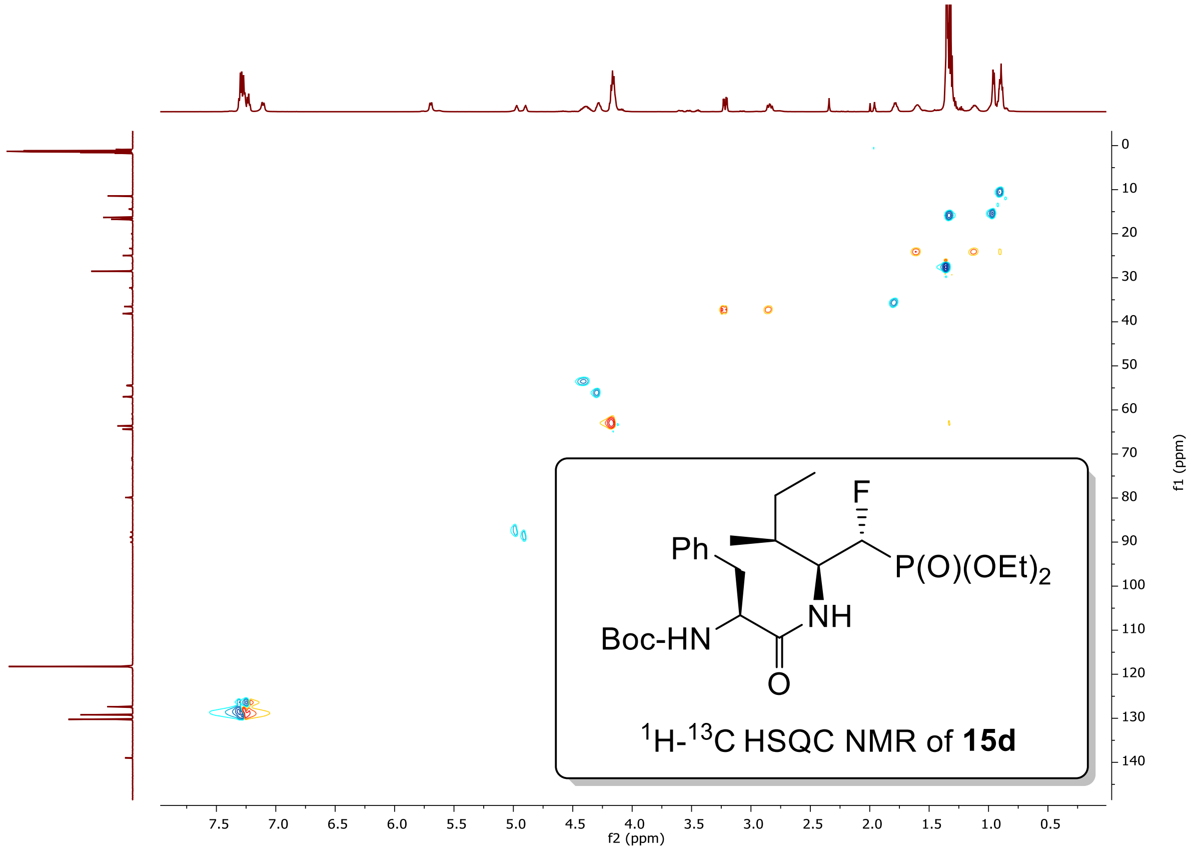

Ph
F
P(O)(OEt)2
Boc-HN
NH
O
1H-13C HSQC NMR of 15d
f2 (ppm)
f1 (ppm)

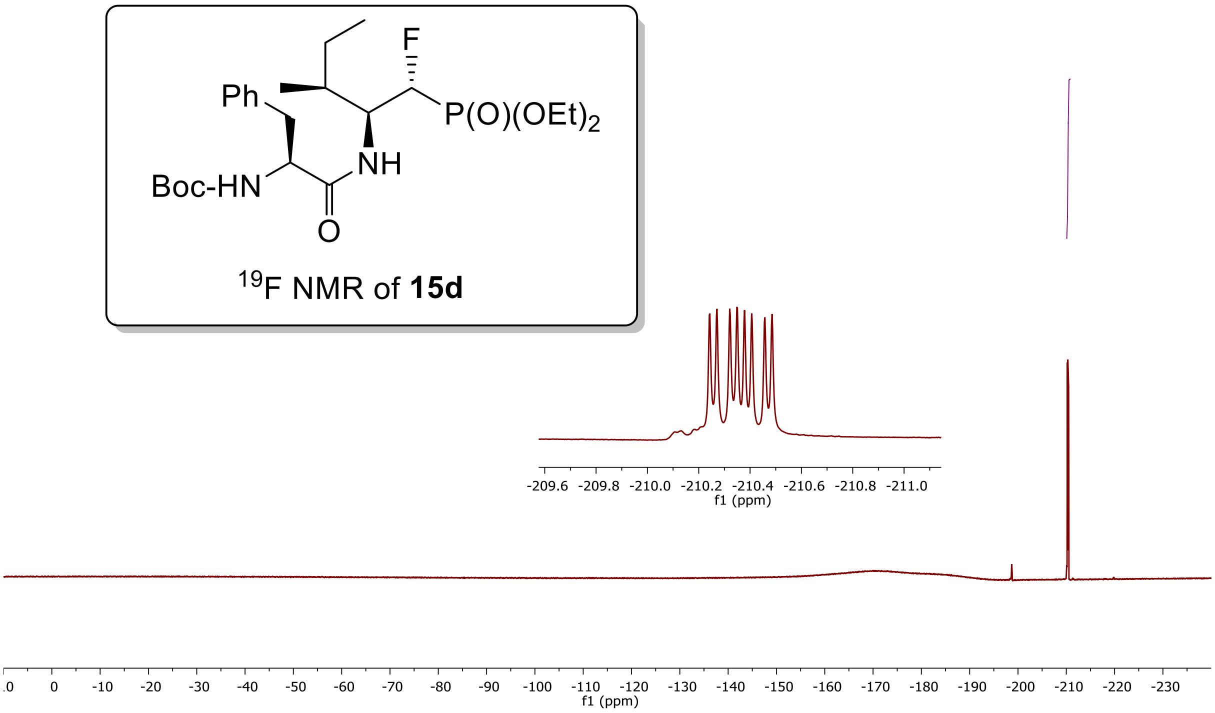

Ph
F
P(O)(OEt)2
Boc-HN
NH
O
19F NMR of 15d
f1 (ppm)

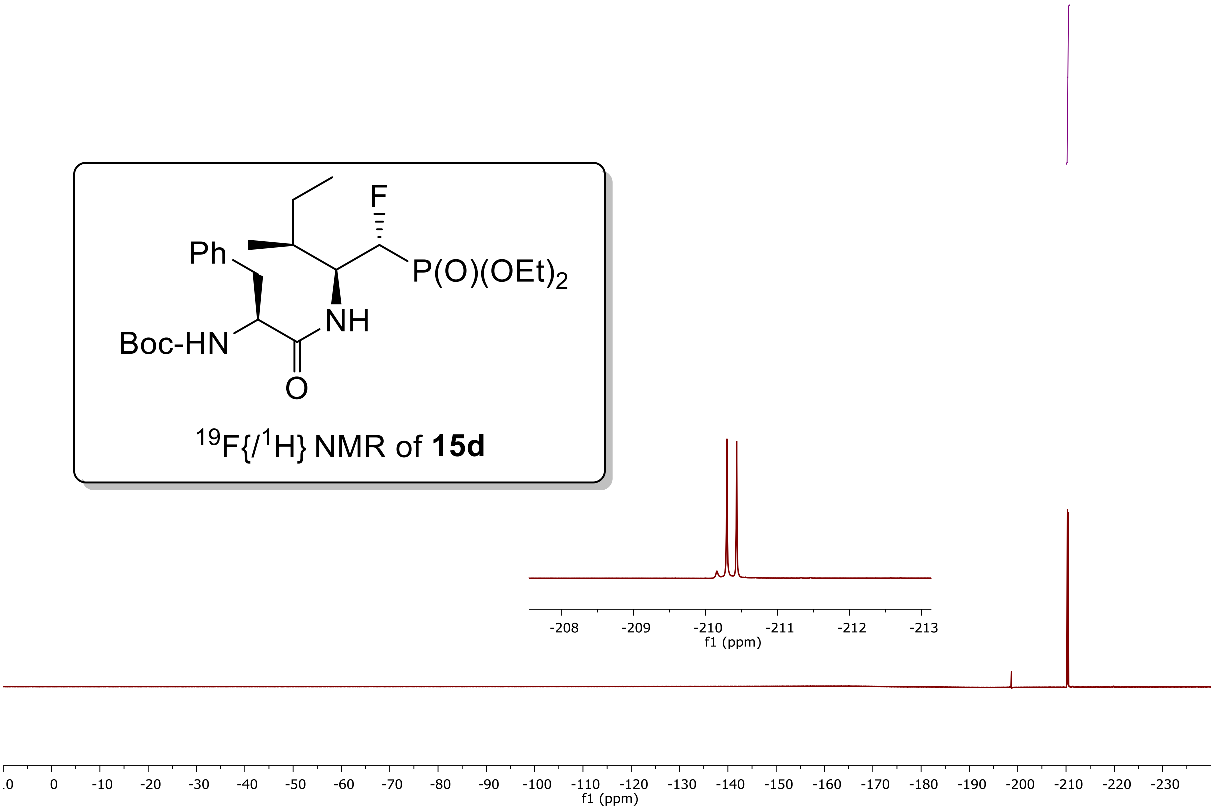
F
Ph
P(O)(OEt)$_2$
NH
Boc-HN
O
^{19}F{/^{1}H} NMR of **15d**
-208
-209
-210
-211
-212
-213
f1 (ppm)
0
-10
-20
-30
-40
-50
-60
-70
-80
-90
-100
-110
-120
-130
-140
-150
-160
-170
-180
-190
-200
-210
-220
-230
f1 (ppm)

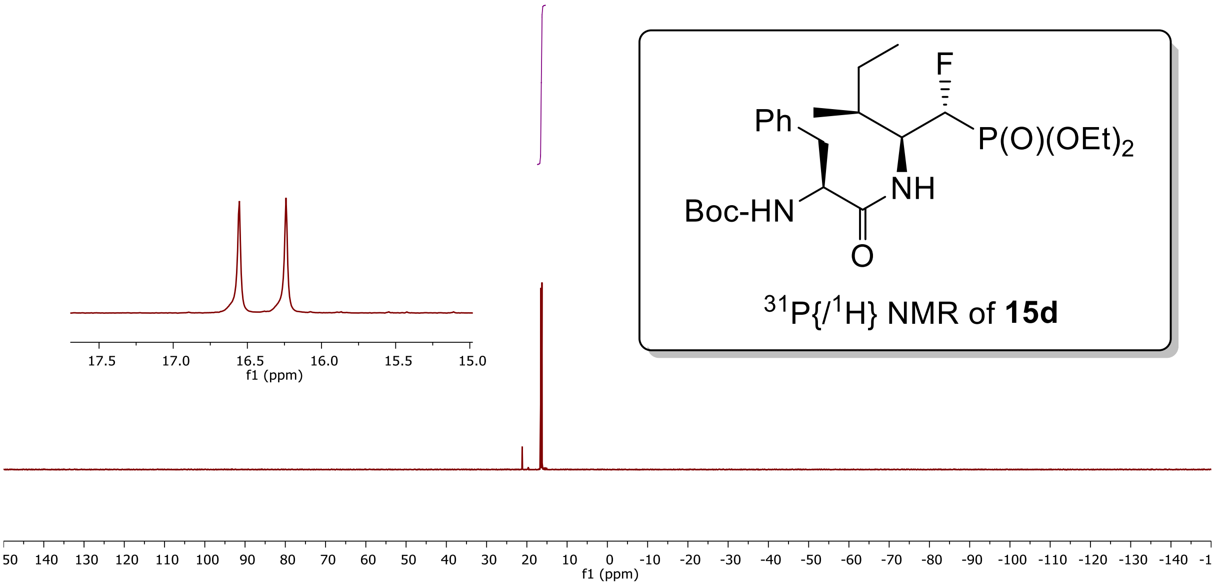
F
Ph
P(O)(OEt)$_2$
NH
Boc-HN
O
^{31}P{/^{1}H} NMR of **15d**
17.5
17.0
16.5
16.0
15.5
15.0
f1 (ppm)
140
130
120
110
100
90
80
70
60
50
40
30
20
10
0
-10
-20
-30
-40
-50
-60
-70
-80
-90
-100
-110
-120
-130
-140
f1 (ppm)

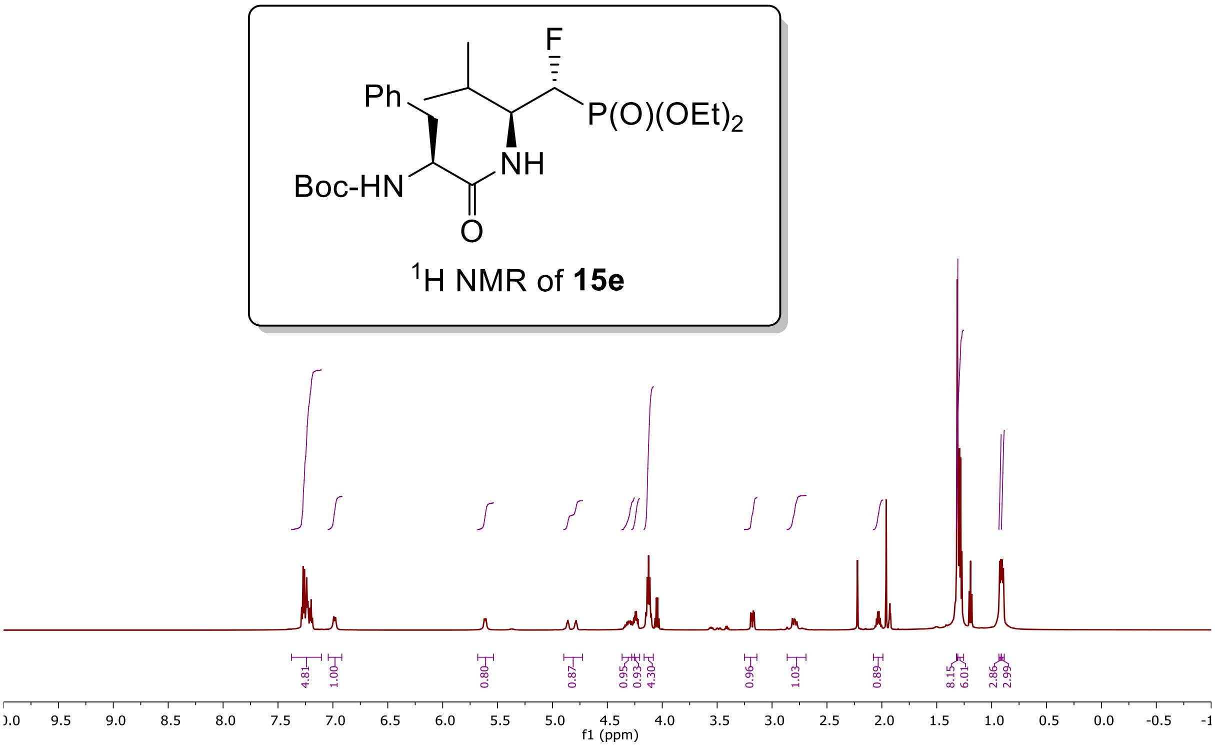
F
Ph
P(O)(OEt)2
NH
Boc-HN
O
1H NMR of 15e
4.81
1.00
0.80
0.87
0.95
0.93
4.30
0.96
1.03
0.89
8.15
6.01
2.86
2.99
f1 (ppm)

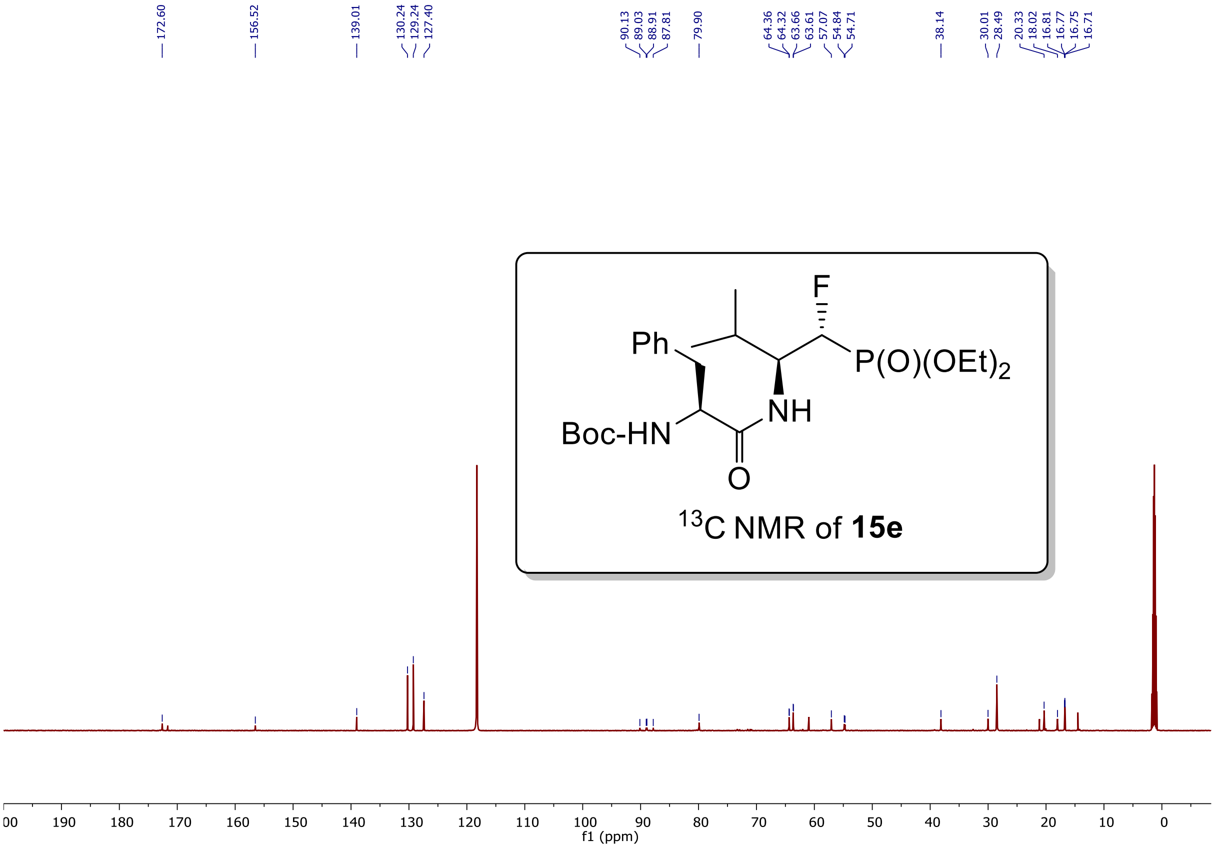
172.60
156.52
139.01
130.24
129.24
127.40
90.13
89.03
88.91
87.81
79.90
64.36
64.32
63.66
63.61
57.07
54.84
54.71
38.14
30.01
28.49
20.33
18.02
16.81
16.77
16.75
16.71
F
Ph
P(O)(OEt)2
NH
Boc-HN
O
13C NMR of 15e
f1 (ppm)

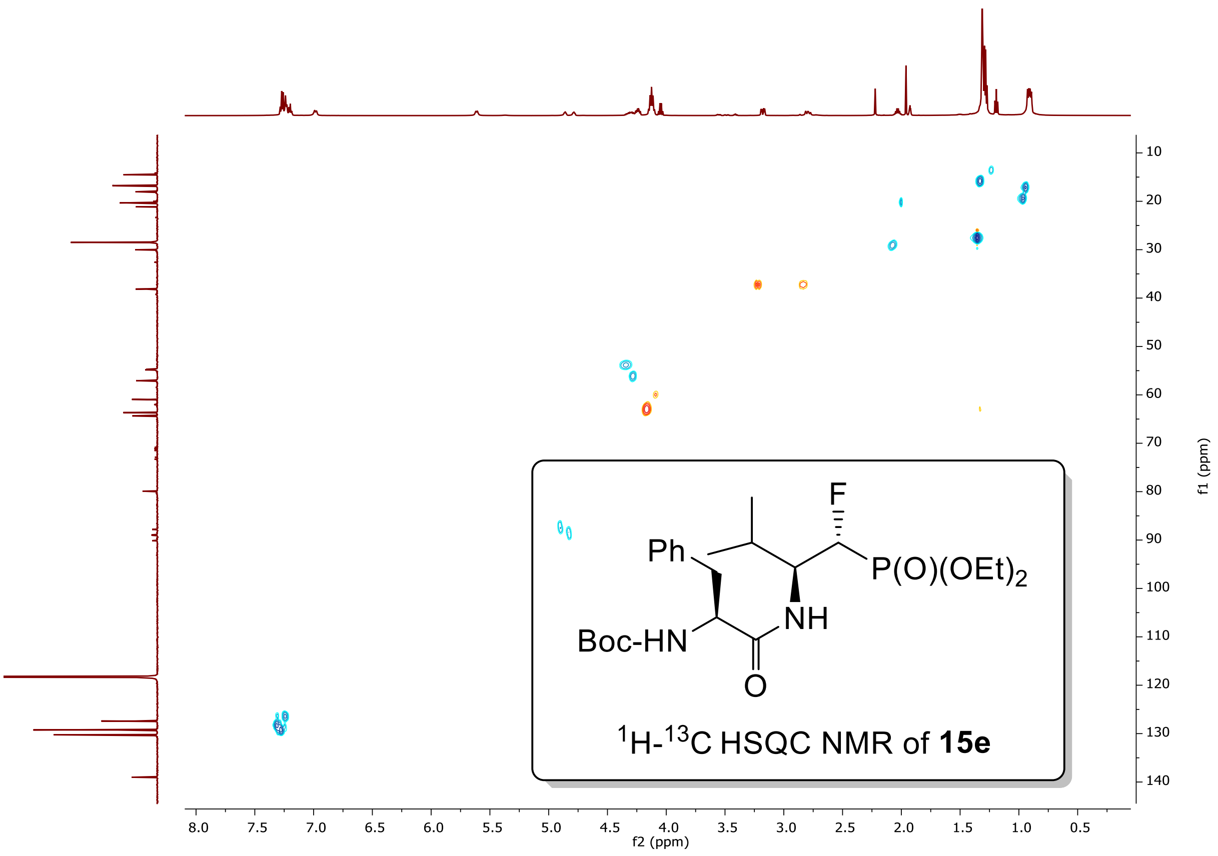

F
Ph
P(O)(OEt)2
NH
Boc-HN
O
1H-13C HSQC NMR of 15e
f2 (ppm)
f1 (ppm)

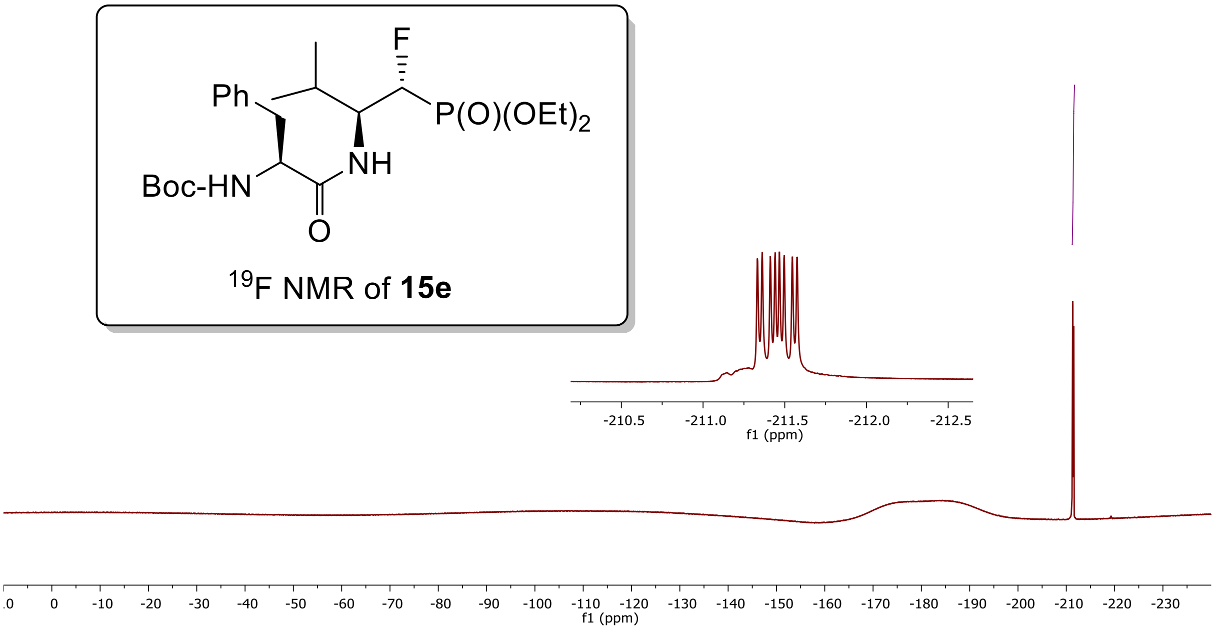

F
Ph
P(O)(OEt)2
NH
Boc-HN
O
19F NMR of 15e
f1 (ppm)

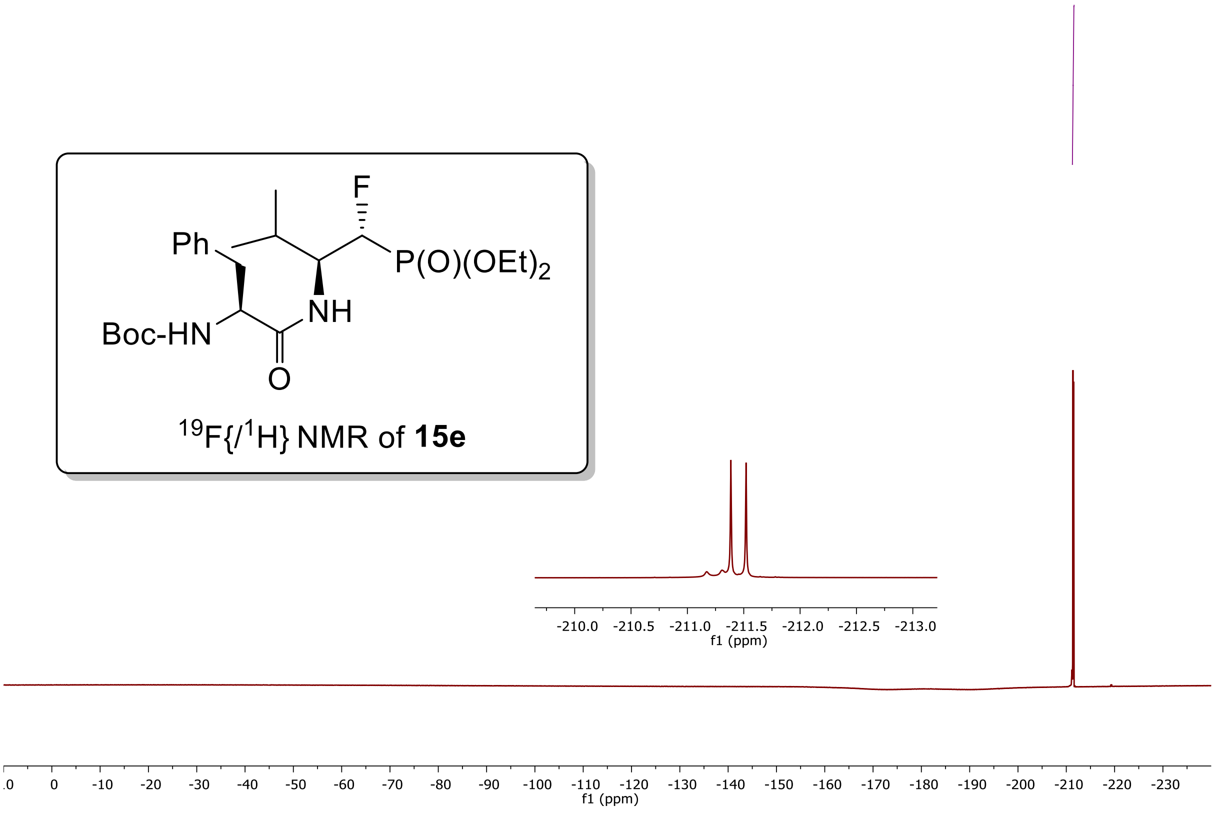
Ph
Boc-HN
NH
O
F
P(O)(OEt)2
19F{/1H} NMR of 15e
-210.0 -210.5 -211.0 -211.5 -212.0 -212.5 -213.0
f1 (ppm)
f1 (ppm)

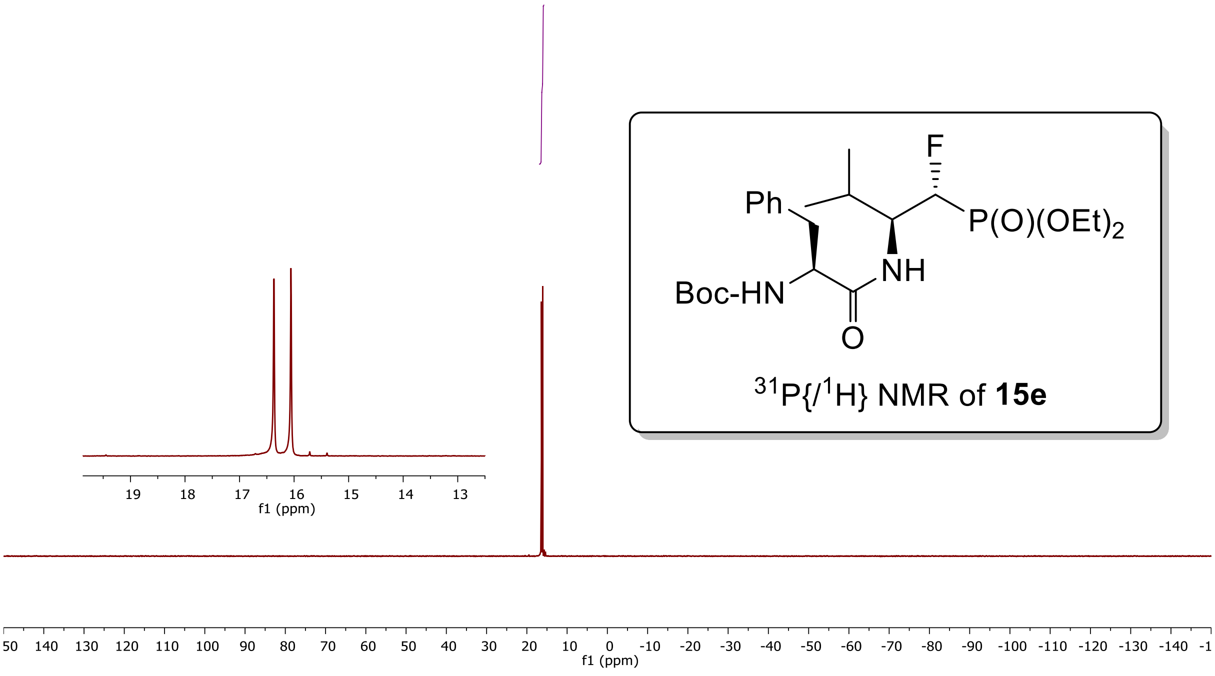
Ph
Boc-HN
NH
O
F
P(O)(OEt)2
31P{/1H} NMR of 15e
19 18 17 16 15 14 13
f1 (ppm)
f1 (ppm)

4. Crystallographic data

Table S2. Selected geometrical data.

	**14c**
P1-O2	1.466(2)
P1-O3	1.566(2)
P1-O6	1.560(2)
P1-C9	1.816(3)
C10-N10	1.499(3)
O2-P1-O3	115.86(12)
O2-P1-O6	116.95(13)
O2-P1-C9	109.40(13)
O3-P1-O6	103.00(11)
O3-P1-C9	107.32(12)
O6-P1-C9	103.20(13)
P1-O3-C4-C5	-166.9(2)
P1-O6-C7-C8	150.6(3)
P1-C9-C10-N10	160.01(18)
P1-C9-C10-C11	-77.3(3)
O2-P1-C9-C10	-52.1(2)
O2-P1-C9-F9	-177.03(18)

Table S3. Hydrogen bond data (Å, °) with s.u.'s in parentheses.

D	H	A	D-H	H···A	D···A	D-H···A
N10	H10A	O12A^{i}	0.91	1.94	2.798(3)	157
N10	H10B	O11A^{ii}	0.91	1.95	2.839(3)	166

N10	H10C	O2^{ii}	0.91	1.84	2.749(3)	176
O21A	H21A	O1W	0.84	1.68	2.495(3)	165
O1W	D1A	O12A^{i}	0.85	1.96	2.783(3)	164
O1W	D1B	O11A^{iii}	0.85	1.88	2.728(3)	178

Symmetry codes: ^{i} 1-x,-1/2+y,1/2-z; ^{ii} -1+x,y,z; ^{iii} 2-x,-1/2+y,1/2-z;

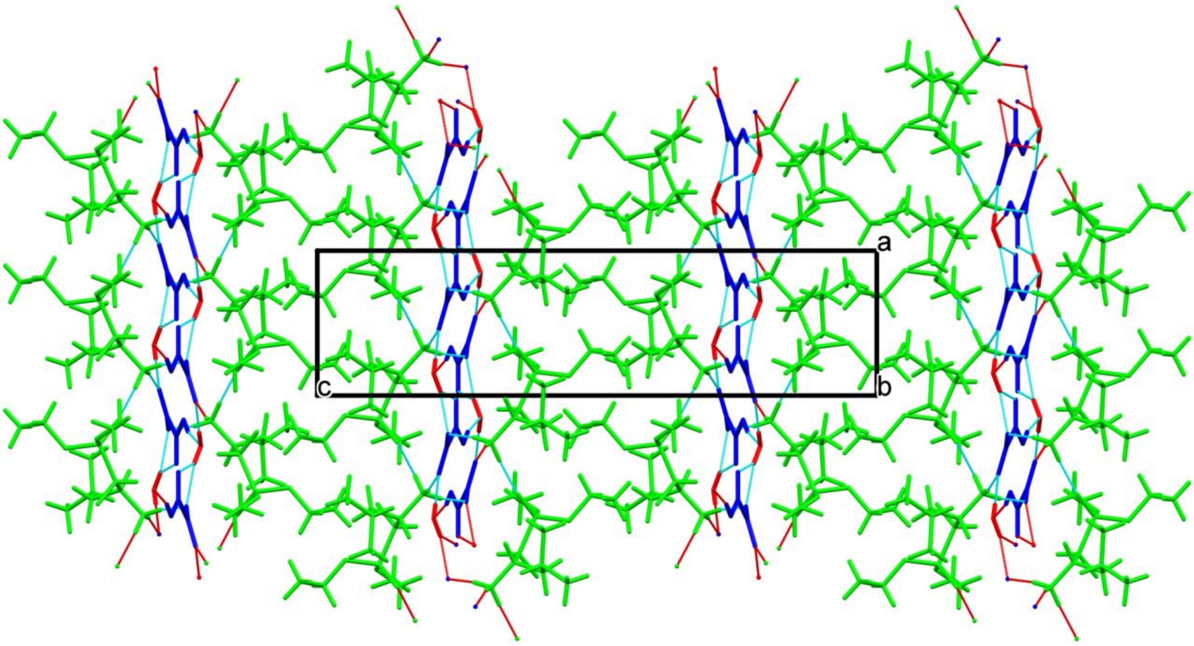

Figure S1. Crystal packing of **14c**; hydrogen bonds are shown as dashed blue lines.
